# Characterization and evolutionary insights into complete mitochondrial genome of *Sedum sarmentosum* within the family Crassulaceae

**DOI:** 10.3389/fpls.2026.1710625

**Published:** 2026-02-06

**Authors:** Meiling Qin, Peng Lü, Min Tang, Shaoshuai Yu, Xun Gong

**Affiliations:** 1 Department of Rheumatology & Immunology, Affiliated Hospital of Jiangsu University, Zhenjiang, Jiangsu, China; 2 School of Life Sciences, Jiangsu University, Zhenjiang, Jiangsu, China; 3 Department of Pharmacy, Affiliated People’s Hospital of Jiangsu University, Zhenjiang, Jiangsu, China

**Keywords:** mitogenome, phylogeny, ptgenome, RNA editing, *Sedum sarmentosum* bunge

## Abstract

*Sedum sarmentosum* Bunge is a perennial succulent plant of medicinal significance within the Crassulaceae family. To investigate its mitochondrial genome (mitogenome), structure, gene composition, and evolutionary implications, we assembled the complete mitogenome and plastid genome (ptgenome) of *S. sarmentosum* using high-fidelity sequencing data. The resulting mitogenome is a circular DNA molecule of 156,727 bp with a GC content of 45.30%, encoding 30 protein-coding genes (PCGs), eight tRNAs, and two rRNAs. Analyses identified 78 simple sequence repeats, two tandem repeats, and 30 dispersed repeats. A total of 617 potential RNA-editing sites were predicted, predominantly occurring at the second codon positions of mitochondrial PCGs. In addition, 18 mitochondrial plastid DNA transfer events were identified between the mitochondrial and chloroplast genomes, which included both tRNA and partial protein-coding gene segments. Moreover, the regional boundaries of chloroplasts of *S. sarmentosum* was identified, consisting of a large single-copy (LSC) region (81,798 bp), a small single-copy (SSC) region (16,671 bp), and two inverted repeat (IR) regions (25,778 bp each). Phylogenetic analyses based on mitogenomes of 26 species revealed that *S. sarmentosum* is closely related to members of the *Rhodiola* genus within Crassulaceae, providing new insights into evolutionary relationships among Saxifragales. Furthermore, codon usage bias, selection pressure analysis, and nucleotide diversity assessments uncovered lineage-specific patterns of molecular evolution, highlighting the balance between purifying and positive selection in shaping mitochondrial gene divergence. Altogether, this study contributes to our understanding of mitogenomic architecture, evolutionary adaptation, and phylogenetic placement of *S. sarmentosum*, and offers a valuable genomic resource for future studies in plant evolution, functional genomics, and molecular breeding.

## Introduction

*Sedum sarmentosum*, commonly known as “stringy stonecrop” or “golden moss,” is a perennial herbaceous plant belonging to the family Crassulaceae (Chen and Sun, 2021; Xu et al., 2015). It is widely distributed across East Asia, particularly in China, Korea, and Japan, where it grows in rocky, mountainous, and often disturbed habitats. Owing to its robust adaptability, rapid propagation, and tolerance to drought and poor soil conditions, *S. sarmentosum* has attracted attention in ecological restoration and urban greening. *S. sarmentosum* prefer sunny environment, but it can also survive in a semi-shade environment and grow in drought and barren soil. Furthermore, it has been used to treat hepatic disorders (e.g., hepatitis, jaundice) and gastrointestinal infections like dysentery (Chen and Sun, 2021; Yu et al., 2021). Its flavanones scavenge free radicals, mitigating oxidative stress and inflammation (Yu et al., 2021), while also suppressing tumor cell proliferation and inducing apoptosis (Bai et al., 2016).

Plant organelle genomes, particularly plastid and mitochondrial DNA, serve as vital molecular markers for phylogenetic and evolutionary studies, with plastid sequences being more commonly employed due to their generally conserved structure and uniparental inheritance. While the complete plastome of *S. sarmentosum* (NCBI accession: NC_023085) has been available since 2013, its mitogenome remains uncharacterized. There is a notable gap given the unique evolutionary insights offered by plant mitogenomes, which exhibit distinctive features including frequent recombination events, horizontal gene transfer, and RNA editing phenomena (Moller et al., 2021). Beyond their primary role in lipid metabolism, amino acids metabolism and nucleotides metabolism, it also influences the regulation of apoptosis and calcium signaling pathway (Arimura et al., 2024; Hammani and Giegé, 2014; Horvath and Daum, 2013; Van Aken, 2021). Therefore, the characterization of *S. sarmentosum*’s mitogenome is essential not only for resolving phylogenetic relationships within Saxifragaceae but also for understanding potential mitochondrial-nuclear interactions that may affect the production of its medicinally valuable secondary metabolites, particularly given the well-documented pharmacological importance of this species (Ali et al., 2024; Liberatore et al., 2016; Wang et al., 2024). This study addresses a critical genomic knowledge gap while establishing a foundation for future evolutionary and biotechnological investigations of this economically significant plant.

In this study, the complete assembly and comparative analysis of both mitochondrial and plastid genomes of *S. sarmentosum* were reported. The study identified characteristic RNA editing sites in mitochondrial protein-coding genes, delineated mitochondrial plastid sequences (MTPTs) and chloroplast boundaries, and reconstructed phylogenetic relationships among 26 species across four plant orders. Evolutionary analyses revealed signatures of selection pressure and genomic synteny within Saxifragales. These findings provide essential genomic resources for understanding the evolutionary history of *S. sarmentosum* and establish a foundation for future investigations into this medicinally important species.

## Materials and methods

### Collection of plant materials, library preparation, and sequencing

**Figure 1:**
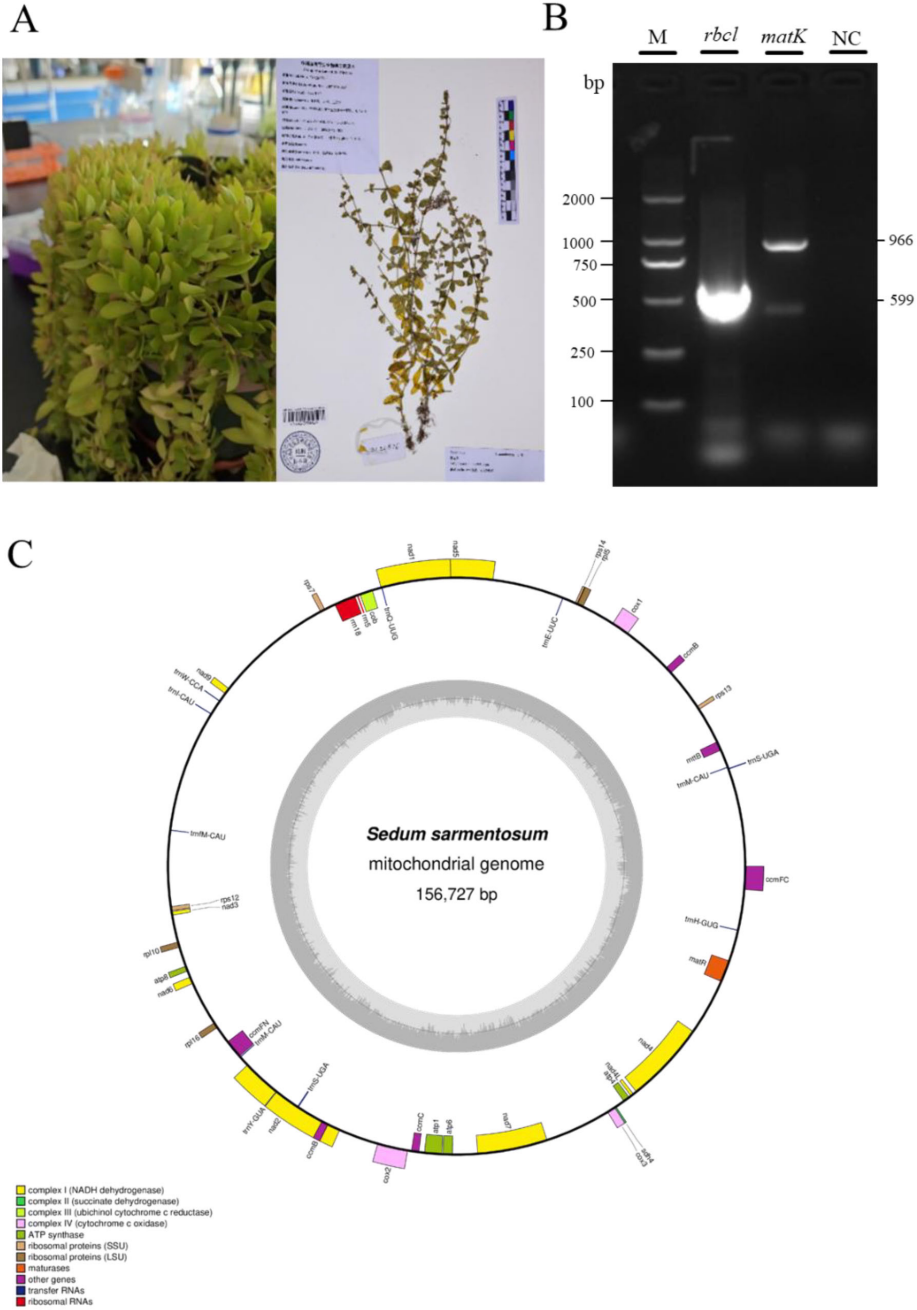
Species identification and mitogenome annotation of *S. sarmentosum*. **(A)** Morphological features of *S. sarmentosum*. The left image displays a living specimen collected in the field, while the right image shows a voucher specimen (KUN1569988) deposited in the Herbarium of the Kunming Institute of Botany, Chinese Academy of Sciences, as recorded in the National Plant Specimen Resource Center. **(B)** Verification of species identity using DNA barcoding. PCR amplification of the chloroplast genes *rbcl* and *matK* produced clear bands, confirming the taxonomic assignment of the specimen. Lane M: 2000 bp DNA marker; Lane NC: negative control. **(C)** Annotated circular map of the mitogenome of *S. sarmentosum*. Genes on the outer circle are transcribed clockwise, and those on the inner circle are transcribed counterclockwise. The innermost grey graph represents GC content variation across the genome. Color-coded gene categories include protein-coding genes, tRNAs, rRNAs, and ORFs.

Tender leaves of *S. sarmentosum* were obtained from Jiangsu University, located in Zhenjiang Province, China (coordinates: 32.11 N, 119.35 E). Before library construction, morphological characteristics of collected samples were compared with the specimen from the National Plant Specimen Resource Center of China Digital Herbarium, with the specimen number KUN1569988, to ensure accurate identification of the sample species and the physical specimen is stored at the Herbarium, Kunming Institute of Botany, Chinese Academy of Sciences (**Figure 1A**). The leaves are simple, arranged in whorls of three, fleshy, lanceolate to oblanceolate, with an entire margin and an acute apex. These observed morphological characteristics are consistent with the standard description of *S. sarmentosum*. After that, the leaves were thoroughly rinsed with DEPC-treated water and immediately stored at −80 °C until processing. Genomic DNA was isolated from *S. sarmentosum* using a modified CTAB protocol optimized for high molecular weight extraction. DNA integrity was verified through 0.75% agarose gel electrophoresis, while purity and concentration were determined using dual quantification methods: UV spectrophotometry (NanoDrop One, Thermo Fisher Scientific) and fluorescent dye-based detection (Qubit 3.0 Fluorometer, Life Technologies). For long-read sequencing, high-quality DNA was processed using the SMRTbell Express Template Preparation Kit 2.0 (Pacific Biosciences), with final library sequencing performed on the PacBio Sequel II system. Sequencing efforts included approximately 9 Gb of PacBio HiFi long-read data and 23 Gb of paired-end Illumina data. Second-generation sequencing provided a total of 70,152,666 clean reads, while third-generation sequencing yielded 256,990 clean reads.

### DNA extraction by optimized CTAB based protocol

Genomic DNA was extracted from the samples using an optimized cetyltrimethylammonium bromide (CTAB) protocol. Briefly, finely powdered frozen tissue was mixed with pre-heated extraction buffer and Proteinase K in a 15 mL tube, vortexed thoroughly, and incubated at 65°C for 3 hours with gentle inversion every 10 minutes. The sample was then centrifuged at 4°C. For third-generation sequencing library preparation, the supernatant was transferred to a tube containing DNA extraction solution, gently mixed, and centrifuged again. The aqueous phase was collected, treated with RNase A at 37°C for 20 minutes, and mixed again with DNA extraction solution. After another centrifugation step, isopropyl alcohol was added to the supernatant to precipitate the DNA, followed by incubation at -20°C for 40 minutes. The DNA pellet was recovered by centrifugation, washed twice with 80% ethanol, air-dried, and finally resuspended in TE buffer.

### Application of DNA barcoding in species identification

The core plant barcode (*rbcl* + *matK*) was amplified with genomic DNA for high-accuracy species identification (**Figure 1B**). The specific primers and PCR conditions employed are outlined in **Table 1** (Liu et al., 2025; Zhou et al., 2025). As shown in the table, the primer pairs for *rbcl* amplification (rbcl-F and rbcl-R) and for *matK* (matK-F and matK-R) were set according to (Liu et al., 2016)). Then, the PCR products were submitted to Sangon Biotech (Shanghai) for bidirectional Sanger sequencing. The resulting sequences were subsequently aligned against reference barcodes in the Barcode of Life Data Systems (BOLD) database (https://www.boldsystems.org) for taxonomic validation (Ratnasingham and Hebert, 2007; Ratnasingham et al., 2024). Finally, the species was identified in accordance with alignment scores, sequence similarity, and E-values.

**Table 1:** Primer sequences and PCR conditions for target gene amplification.

Gene	Primers	Product length (bp)	Sequence 5’-3’	PCR procedure	Refs
*rbcl*	rbcl-F	599	ATGTCACCACAAACAGAGACTAAAGC	95°C 3 min; 94°C 15s, 53°C 30s, 72°C 1 min (35 cycles); 72°C 5 min	(Gong et al., 2024; Liu et al., 2016, Liu et al., 2025; Zhou et al., 2025)
	rbcl-R		GTAAAATCAAGTCCACCGCG		
*matK*	matK-F	966	CGTACAGTACTTTTGTGTTTACGAG		
	matK-R		TAATTTACGATCAATTCATTC		

### Organelle genome assembly and annotation

The mitogenome of *S. sarmentosum* was reconstructed through a two-step process. First, the mitogenome of *S. sarmentosum* was reconstructed using a dual-stage assembly strategy. Initial identification of mitochondrial-derived reads was performed using BLASTn (v2.13.0+) by aligning HiFi sequencing reads against conserved mitochondrial gene sequences from *Sedum plumbizincicola* (NC_069572.1) as reference genome (Chen et al., 2015; Ding et al., 2022). The parameters were set as: “-value 1e-5 -outfmt 6 -max_hsps 10 -word_size 7 -task BLASTn-short.” Then, the assembly was performed based on mitochondrial HiFi reads in Unicycler (v0.5.1), with default parameters, and finally obtain a master circle of the *S. sarmentosum* mitogenome was obtained (Wick et al., 2017). The complete mitogenome was annotated with GeSeq webset (https://chlorobox.mpimp-golm.mpg.de/geseq.html) (Tillich et al., 2017) based on two reference mitogenomes, *S. plumbizincicola* (NC_069572) and *Rhodiola rosea* (PP024540) (Zhao and Zhang, 2018). Annotion errors were corrected with GeSeq web server. The mitogenome maps of *S. sarmentosum* were visualized with OGDRAW (v1.3.1) (https://chlorobox.mpimp-golm.mpg.de/OGDraw.html) (Greiner et al., 2019).

### Repeats and codon usage analysis

The organelle genome is much smaller than the nuclear genome, and the repeat sequence is also simple, so the classification of repeats is not as complicated as the nuclear genome. The repetitive elements were classified into three distinct types according to their structural organization: simple sequence repeats (SSRs), tandem duplications, and interspersed repetitive sequences. The SSRs were identified with misa.pl (v2.1) (Thiel et al., 2003) and the thresholds of mono-, di-, tri-, tetra-, penta-, and hexa- were set respectively as 10, 5, 4, 3, 3, and 3. Additionally, a maximum length variation threshold of 100 bp was established for SSRs. Tandem repeat detection was performed using Tandem Repeats Finder (TRF v4.09) (Benson, 1999) based on parameters ‘2 7 7 80 10 50 2000 -f -d -m’. The dispersed repeats were identified by REPuter tool (https://bibiserv.cebitec.uni-bielefeld.de/reputer) (Kurtz et al., 2001) with Maxumum Computed Repeats set as 500 and Minimal Repeat Size set as 30. All repeats were visualized with Circos (v0.69-8) (Krzywinski et al., 2009).

The different frequency of codon use of amino acids is called codon use bias. The analysis of the code use preference in genes can promote cover the origin and evolution of these genes (Hershberg and Petrov, 2008). In addition, the frequency of codon use is also related to gene expression. If a gene preferentially utilizes codons that match more abundant tRNA species, the translation efficiency can be enhanced due to faster codon-anticodon pairing, thereby potentially increasing its expression level. Such codon usage bias may indicate the gene’s essential role in maintaining fundamental biological processes of the species (Gouy and Gautier, 1982). The relative synonymous codon usage (RSCU) values, which used to measure the phenomenon that among different synonymous codons encoding the same amino acid, some codons are used significantly more frequently than others, were calculated with the software Phylosuite (v1.2.3) and visualized with R package ‘ggplot2’ (Zhang et al., 2020).

### Assembly of chloroplast genome and identification of mitochondrial-plastid DNA transfer

The chloroplast genome of *S. sarmentosum* was assembled with GetOrganelle (v1.7.1) based on clean Illumina short reads, using mitogenome from *S. plumbizincicola* (NC_069572) as reference sequences (Jin et al., 2020). The geneome circle was annotated with the CPGAVAS2 web server (http://47.96.249.172:16019/analyzer/annotate) (Shi et al., 2019). The Visualization of the plastome maps for *S. sarmentosum* was achieved using OGDRAW (v1.3.1) (https://chlorobox.mpimp-golm.mpg.de/OGDraw.html) (Lohse et al., 2007).

Fragment communication between mitochondria and chloroplasts is very common in higher plants, and about 5% to 10% of the mitogenome of different species can be found in the chloroplast genome. What’s more, some of the repeated sequences found in mitochondrial genomes may be derived from multiple transfers of the same plastid fragment (Wang et al., 2018). MTPTs were identified by BLASTn (v2.13.0). This analysis is of great significance for exploring the mechanism of horizontal gene transfer in chloroplast genome and its role in plant evolution. The database was constructed with mitogenome and the plastome was used as the query sequence in BLASTn, with parameters set to ‘evalue 1e-6’. Finally, the MTPTs were visualized with TBtools (v2.010) (Chen et al., 2020) and annotated via the CPGAVAS2 website (http://47.96.249.172:16019/analyzer/annotate) and OGDRAW serves (https://chlorobox.mpimp-golm.mpg.de/OGDraw.html).

### RNA-editing site prediction and structure prediction of tRNAs

RNA editing is a widespread phenomenon in plant mitochondrial genomes, in which the base of RNA molecules is changed to regulate gene expression and protein function (Lukeš et al., 2021). The prediction of RNA-editing sites was performed using the Deepred-Mt (available at https://github.com/aedera/deepredmt) (Edera et al., 2021). For this analysis, the PCGs of *S. sarmentosum* were extracted and the prediction performed with the 40 nucleotides flanking a given cytidine. The alignment results were visualized by Jalview 2.11.4.1 (Waterhouse et al., 2009).

tRNA is not only involved in protein synthesis, but also plays a variety of important functions in cells. It is crucial for understanding the genetic information transmission process of cells to understand the structure and characteristics of tRNA. For reconstruction of cloverleaf structures of tRNAs, tRNAscan-SE 2.0 was utilized and it can only predict the secondary structure of tRNAs (Chan et al., 2021). The alignment results were visualized by ESPript 3.0 (https://espript.ibcp.fr/ESPript/ESPript/index.php) (Robert and Gouet, 2014).

### Selective pressure analysis and nucleotide diversity analysis

Selective pressure represents the external pressure driving species adaptation to environmental conditions through differential survival and reproduction. In genetics, ω= Ka/Ks indicates the ratio between non-synonymous mutations (Ka) and synonymous mutations (Ks). It is generally believed that synonymous mutations do not change the encoded amino acid and therefore have less effect on fitness, whereas non-synonymous mutations may affect phenotypes and thus be regulated by natural selection. The Ka/Ks value of 26 PCGs in 25 relatives was calculated by KaKs_calculator 2.0 (Wang et al., 2010).

Pi value is an important indicator to measure the level of genetic variation in a population. The higher the Pi value, the greater the difference in nucleotide sequence in the population, thus reflecting a higher level of genetic diversity, which may indicate greater potential of the population to adapt to environmental changes. The Pi value of 23 PCGs in Saxifragales and Caryophyllales was calculated via DnaSp tool (Rozas et al., 2017).

### Phylogenetic and collinear analysis of *S. sarmentosum* mitogenome

A total of 25 species from four orders (Caryophyllales, Rosids, Santalales and Saxifragales) closely related to *S. sarmentosum* were selected, and *Beta macrocarpa* from Caryophyllales were set as the outgroup for phylogenetic analysis (**Supplementary Table S11**). All GenBank files were read using Phylosuite (v1.2.3) (Zhang et al., 2020), and the PCGs were standardized and extracted. Sequence alignments were generated with MAFFT (v7.313) (Katoh and Standley, 2013), followed by maximum likelihood phylogenetic reconstruction using IQ-TREE2 (v2.1.4) with default parameters and branch support was assessed with 1000 bootstrap replicates (Minh et al., 2020). Final tree visualization and optimization were performed in iTOL (Letunic and Bork, 2007).

Four Saxifragales mitochondrial genomes served as references for colinear analysis. Using BLASTn (v2.13.0; e-value ≤1e-6), we identified homologous sequences >500bp as conserved syntenic blocks. Genome synteny was visualized using NGenomeSyn (v1.41) and Mauve (Darling et al., 2004; He et al., 2023).

## Results

### Genetic identification of collected specimens

The two pairs of PCR primers successfully achieved specific amplification on the target genes *matK* and *rbcl*, with fragment sizes aligning with expectations (**Table 1**). The database match for the *rbcl* amplification product showed that our collected samples are highly homologous to *S. sarmentosum* (Confidence = 93.63, Similarity = 99.64, **Supplementary Table S1**). Although the *matK* amplification product also ranked *S. sarmentosum* as the top match, it displayed high homology as well (Confidence = 80.77, Similarity = 98.53, **Supplementary Table S2**).

### Genomic features and structure of *S. sarmentosum* mitogenome

The complete mitochondrial genome sequence of *S. sarmentosum* has been deposited in the NCBI GenBank database under accession number PV608516. (https://www.ncbi.nlm.nih.gov/). The resulting mitogenome assembly is a complete circular DNA structure spanning 156,727 bp (**Figure 1C**).

The mitogenome of *S. sarmentosum* contains a total of 40 annotated genes, including 23 core genes, seven ribosomal protein genes, eight transfer RNAs (tRNAs), and two ribosomal RNAs (rRNAs) (**Table 2**). The PCGs are distributed across various functional categories: four genes related to ATP synthase (*atp1, atp4, atp6, atp8*), nine genes associated with NADH dehydrogenase (*nad1, nad2, nad3, nad4, nad4L, nad5, nad6, nad7, nad9*), and one gene encoding ubiquinol cytochrome c reductase (*cob*). Additionally, there are five cytochrome c biogenesis genes (*ccmB*(x2)*, ccmC, ccmFC, ccmFN*), three cytochrome c oxidase genes (*cox1, cox2, cox3*), a single maturase gene (*matR*). Other genes include a transport membrane protein genes (*mttB*), three large subunit ribosomal protein genes (*rpl5*, *rpl10*, *rpl16*), and four small subunit ribosomal protein genes (*rps7, rps12, rps13, rps14*).

**Table 2:** Encoding genes of *S. sarmentosum* mitogenome.

Group of genes		Name of genes
Core genes	ATP synthase	*atp1, atp4, atp6, atp8*
	NADH dehydrogenase	*nad1, nad2, nad3, nad4, nad4L, nad5, nad6, nad7, nad9*
	Ubichinol cytochrome c reductase	*cob*
	Cytochrome c biogenesis	*ccmB*(x2)*, ccmC, ccmFC, ccmFN*
	Cytochrome c oxidase	*cox1, cox2, cox3*,
	Maturases	*matR*
	Transport membrane protein	*mttB*
Ribosomal protein genes	Large subunit of ribosome	*rpl5, rpl10, rpl16*
	Small subunit of ribosome	*rps7, rps12, rps13, rps14*
rRNA genes	Ribosome RNA	*rrn5, rrn18*
tRNA genes	Transfer RNA	*trnE-UUC, trnH-GUG, trnI-CAU, trnfM-CAU, trnM-CAU*(x2)*, trnQ-UUG, trnW-CCA, trnY-GUA*

Numbers in parentheses indicate gene copy counts.

**Figure 2:**
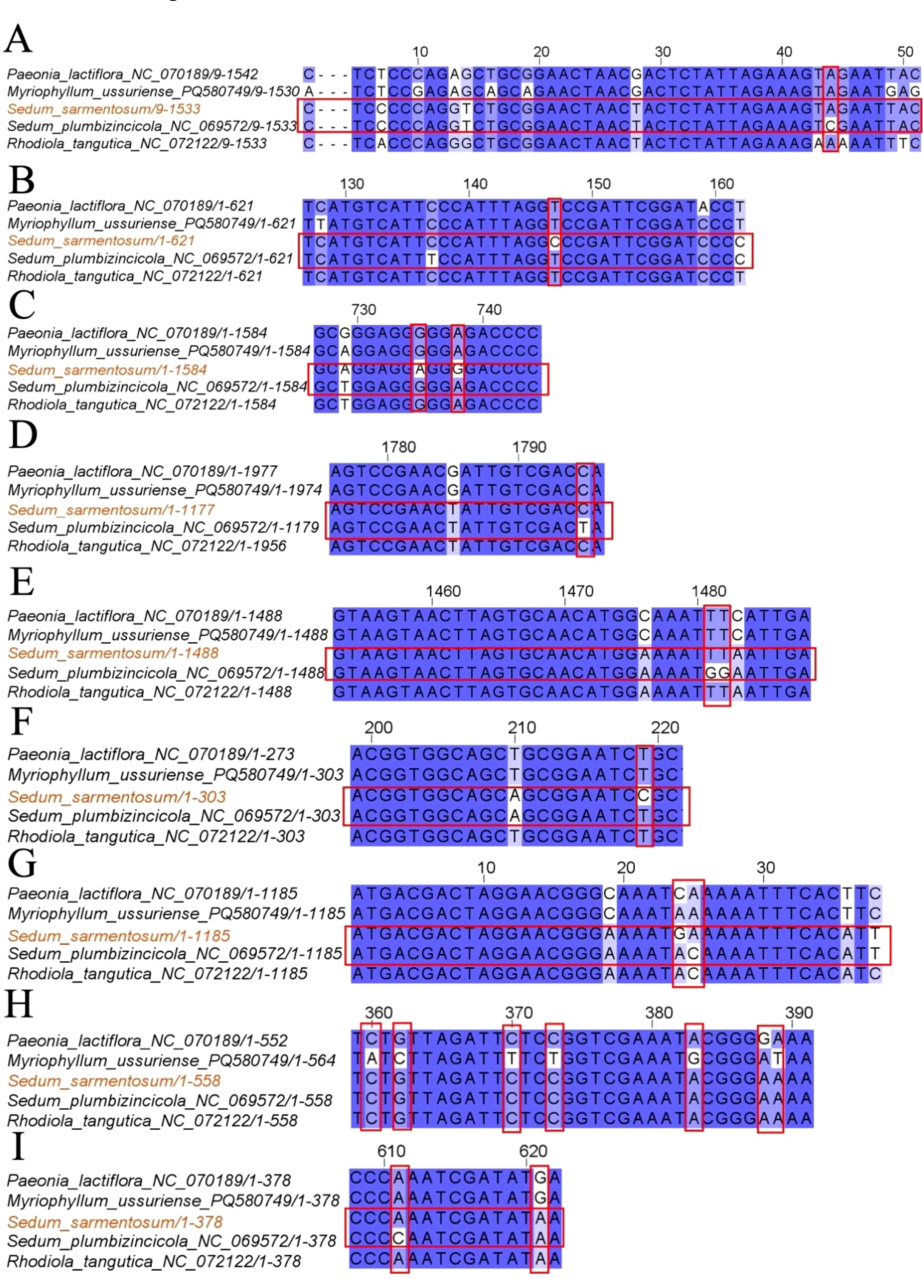
Comparative sequence alignment of mitochondrial PCGs among five Saxifragales species. Multiple sequence alignments of selected mitochondrial PCGs were performed using MAFFT and visualized with ESPript 3.0 to assess nucleotide variation and evolutionary conservation. Panels **(A–I)** display alignment regions for *atp1***(A)**, *ccmB***(B)**, *cox1***(C)**, *matR***(D)**, *nad4***(E)**, *nad4L***(F)**, *nad7***(G)**, *rpl5***(H)**, and *rps12***(I)**. Conserved nucleotides are highlighted in blue, while sequence variations and substitutions are outlined in red boxes. The alignments reveal both highly conserved regions and lineage-specific mutations, providing insight into the molecular evolution of mitochondrial genes in Crassulaceae and related taxa.

Comparative sequence analysis was conducted for eight conserved mitochondrial protein-coding genes (*atp1, ccmB, cox1, matR, nad4, nad4L, nad7, rpl5*, and *rps12*) across five angiosperm mitogenomes: *Paeonia lactiflora* (NC_070189), *Myriophyllum ussuriense* (PQ580749), *S. sarmentosum* (this study), *S. plumbizincicola* (NC_069572), and *R. tangutica* (NC_072122). Alignment results (**Figure 2**) demonstrated strong evolutionary conservation, with most genomic regions showing >90% sequence identity. However, distinct nucleotide and amino acid substitutions (not in blue regions) revealed lineage-specific divergence patterns. Notably, despite shared taxonomic classification within Crassulaceae, *S. sarmentosum* exhibited significant sequence variations compared to *S. plumbizincicola*. These genomic differences may reflect either technical artifacts from divergent sequencing methodologies, or authentic biological variation arising from distinct evolutionary trajectories or ecological adaptations. These insights not only highlight the extent of intraspecific diversity but also pave the way for future studies to elucidate the functional significance of these variations in different ecological contexts.

### Extensive mitogenome rearrangement reveals structural plasticity in saxifragales

**Figure 3:**
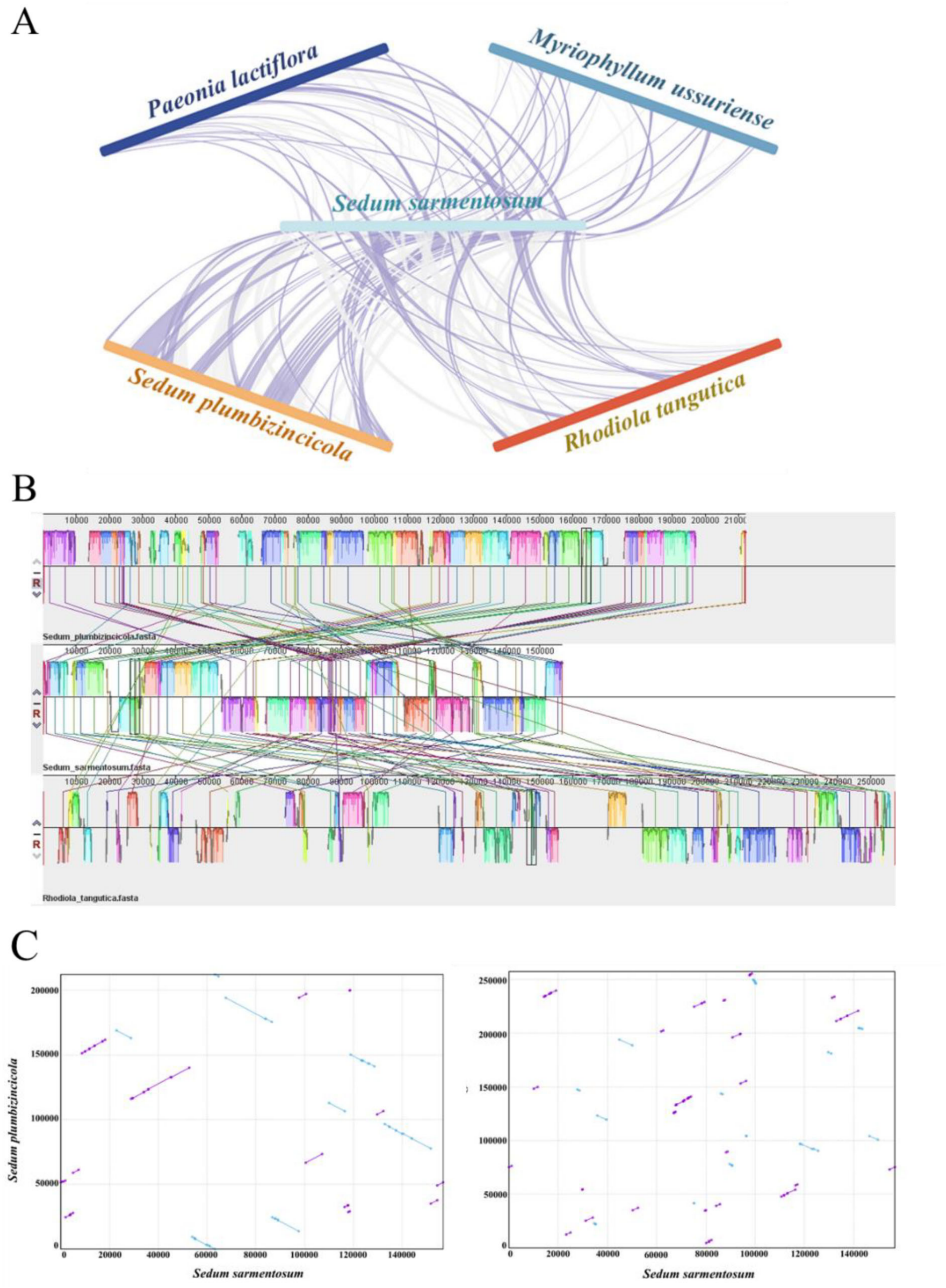
Comparative genomic analysis of mitochondrial genomes among *S. sarmentosum* and related Saxifragales species. **(A)** Synteny visualization of five mitogenomes—*P. lactiflora*, *M. ussuriense*, *S. sarmentosum*, *S. plumbizincicola*, and *R. tangutica*—highlighting homologous collinear blocks. Grey areas indicate covariant blocks in the same direction, while purple areas indicate covariant blocks where inversion occurs. **(B)** Genome-wide alignment of mitochondrial gene regions among *S. sarmentosum*, *S. plumbizincicola*, and *R. tangutica*, illustrating local syntenic relationships and gene rearrangements. Color-coded blocks represent homologous regions across the genomes. **(C)** Dot plot analysis of pairwise sequence similarity between *S. sarmentosum* and the mitochondrial genomes of *S. plumbizincicola* (left) and *R. tangutica* (right). Diagonal lines indicate conserved syntenic regions, while off-diagonal patterns reflect genomic inversions or rearrangements.

Synteny analysis revealed extensive mitogenome rearrangements among *S. sarmentosum* and four related Saxifragales species—*P. lactiflora*, *M. ussuriense*, *S. plumbizincicola*, and *R. tangutica* (**Supplementary Table S9**, **Figure 3A**). The synteny visualization highlights homologous collinear blocks, with purple ribbons representing co-directional alignments and grey indicating inversions. Although numerous homologous regions were detected, they were relatively short and interspersed with structural rearrangements, reflecting a lack of large conserved blocks. Most of the collinear fragments were distributed in discordant orientations and positions across species, suggesting widespread genome reshuffling. Genome-wide alignment (**Figure 3B**) further confirmed this complexity, with fragmented syntenic blocks and limited conservation of gene order across *S. sarmentosum*, *S. plumbizincicola*, and *R. tangutica*. This pattern is indicative of high genomic plasticity and frequent recombination events, likely driven by the abundance of repeats and mobile elements in plant mitogenomes. Dot plot comparisons (**Figure 3C**) supported these findings, showing scattered diagonals and off-axis alignments that reflect numerous inversions and translocations. Overall, these results demonstrate that the mitochondrial genome of *S. sarmentosum* has undergone substantial structural rearrangement and lacks the syntenic stability typically observed within closely related Saxifragales lineages.

### Codon usage analysis

**Figure 4:**
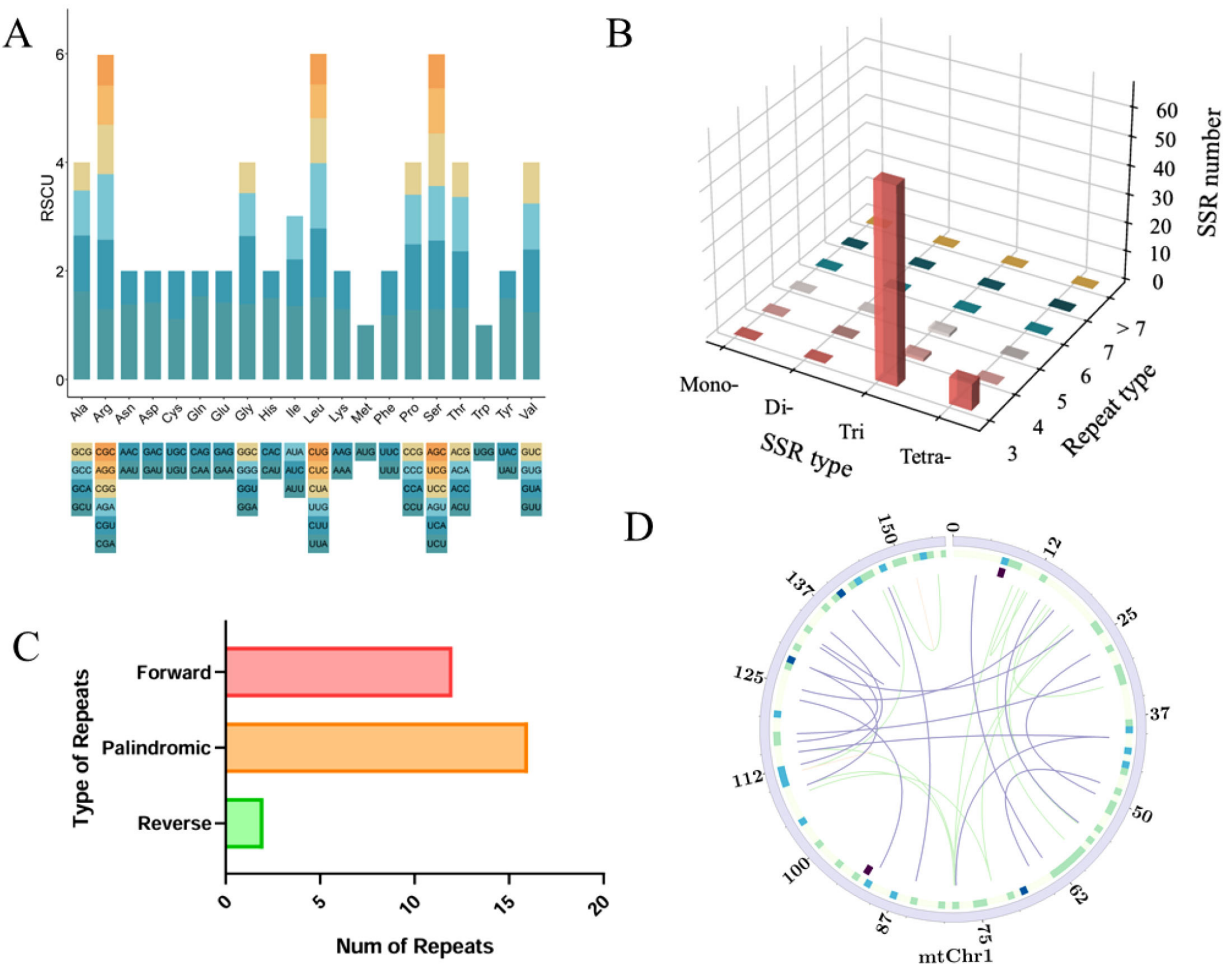
Analysis of codon usage bias and repetitive elements in mitogenome of *S. sarmentosum*. **(A)** RSCU values for each codon in *S. sarmentosum*, illustrating codon preference in protein-coding genes. Codons with RSCU > 1 are preferentially used, indicating a bias in synonymous codon selection. **(B)** Distribution of SSRs by motif type and repeat unit length. Mononucleotide repeats are the most abundant, followed by di- and trinucleotide repeats. **(C)** Classification and frequency of dispersed repeat types. Palindromic repeats are the most prevalent, followed by forward and reverse repeats. **(D)** Circular genome map depicting the spatial distribution of repetitive elements. From outer to inner rings: SSRs, tandem repeats, and dispersed repeats. Connecting lines within the circle represent dispersed repeats—green for forward, purple for palindromic, and red for reverse repeats—highlighting recombinational hotspots and genome plasticity.

Codon use bias can vary greatly among organisms. The observed codon usage bias likely enhances translational efficiency, as preferred codons typically match the most abundant tRNA species within the organism. This tRNA abundance-translation rate correlation occurs because highly expressed tRNAs facilitate faster ribosome translocation during protein synthesis. The mitochondrial PCGs of *S. sarmentosum* had 41 type of codons to encode 20 essential amino acids (**Figure 4A**; **Supplementary Table S3**). Codons with RSCU values greater than 1 were considered to have usage preference. In the mitogenome of *S. sarmentosum*, GCU (Ala), AAU (Asn), GAU (Asp), CAA (Gln), UGU (Cys), GAA (Glu), GGA (Gly), CAU (His), AUU (Ile), UUA (Veilleux et al.), ACU (Thr) and UAU (Tyr), were the most common codons with RSCU values greater than 1.3, while GCG (Ala), CGC (Arg), AGG (Arg), CGG (Arg), AAC (Asn), GAC (Asp), CAG (Gln), GAG (Glu), GGC(Gly), CAC (His), CUG (Veilleux et al.), CUC ((Veilleux et al.), CCG (Waterhouse et al.), AGC (Ser), ACG (Thr) and UAC (Tyr) were the least common codons (less than 0.7). In addition, codons encoded by mitochondrial PCGs in *S. sarmentosum* show a slight preference for A/T at the third codon position. Stop codons show no preference for their use.

### Repeat elements analysis

A total of 89 SSRs, ranging from 9 to 87 base pairs in size, were identified in the mitogenome. These SSRs primarily consisted of trimeric (accounting for 77.53%), tetrameric (accounting for 10.11%) and compound (accounting for 12.36%) types (**Figure 4B**; **Supplementary Table S4**). We identified 30 dispersed repeats, including 12 forward, 16 palindromic, and two reverse repeats (**Figure 4C**; **Supplementary Table S5**), ranging primarily from 19–83 bp. **Figure 4D** displays their genomic distribution in a circular diagram: SSRs (outermost circle), tandem repeats (middle), and dispersed repeats (innermost connections color-coded as green for forward, purple for palindromic, and red for reverse repeats).

### Mitochondrial-plastid DNA transfer and genome characterization

**Figure 5:**
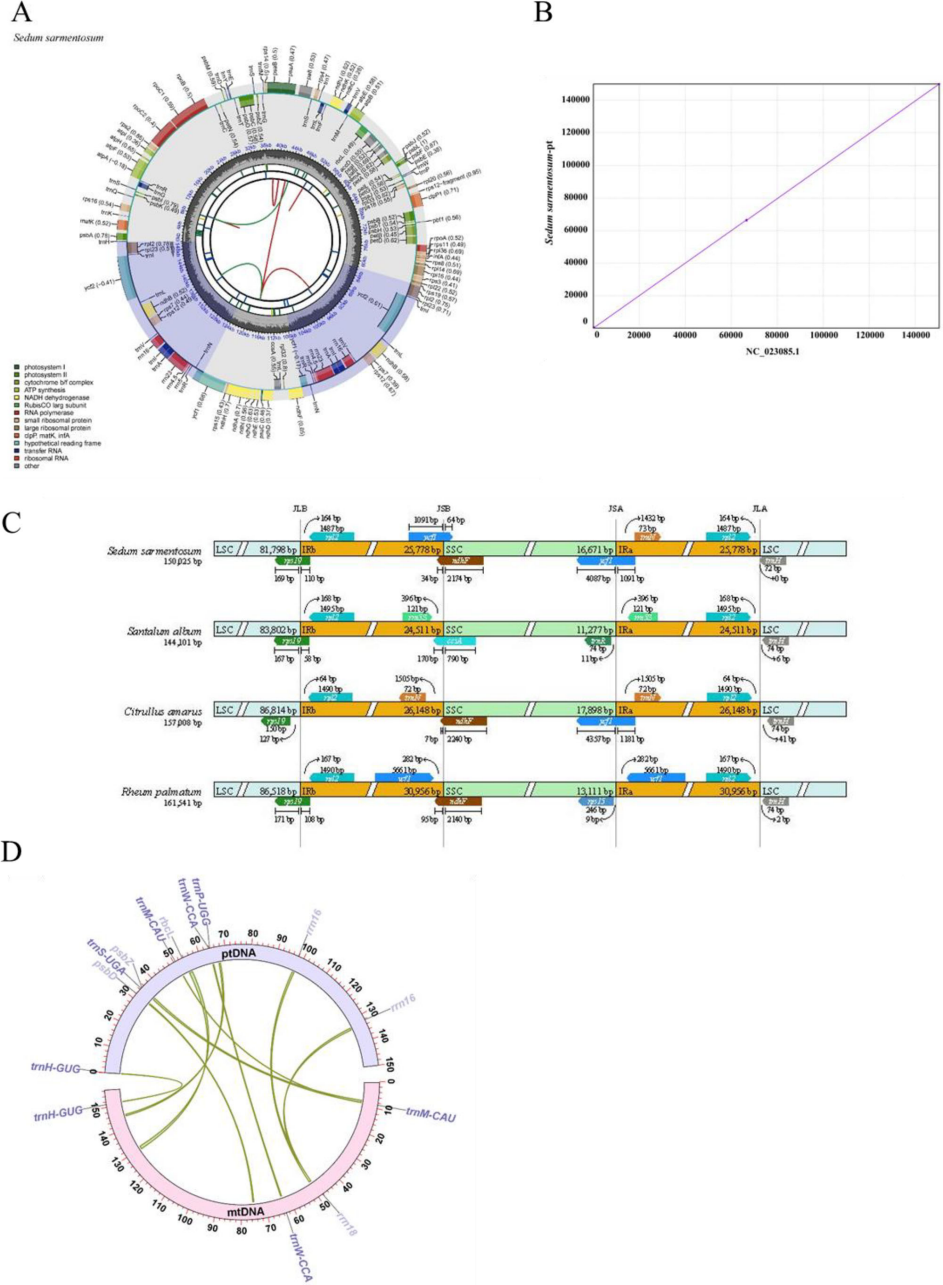
Structural features of chloroplast genome and interorganellar DNA transfer in *S. sarmentosum*.**(A)** Annotated circular map of the complete chloroplast genome of *S. sarmentosum*. Gene categories are color-coded, and the inner circles display GC content and relative sequence depth. Genes located inside and outside the circle represent transcription in opposite directions. **(B)** Comparative analysis between newly assembled genome and the previously published one (NC_023085.1). **(C)** Comparative analysis of junction boundaries among four species reveals structural variation at the borders of IRs, LSC, and SSC regions, highlighting cpDNA diversity in Crassulaceae. **(D)** Visualization of MTPTs between chloroplast genome (cpDNA) and mitogenome (mtDNA) of *S. sarmentosum*. Green ribbons indicate transferred tRNA and partial PCG sequences shared between organelles.

The complete chloroplast genome of *S. sarmentosum* was assembled and annotated, yielding a circular genome of 150,025 bp (**Figure 5A**). To ensure data consistency and assembly completeness, we performed a comparative analysis between our newly assembled genome and the previously published one (NC_023085.1). It can be seen that our new assembly is highly consistent with it, verifying its accuracy (**Figure 5B**). The genome annotation revealed conserved photosynthetic genes typical of angiosperm chloroplasts, including the photosystem II cluster (*psbA-C*), photosystem I core subunits (*psaA-B*), the Calvin cycle enzyme *rbcl*, and electron transport components (*petB, petD*) (Li et al., 2023; Vijayan et al., 2024; Williams-Carrier et al., 2025; Yan et al., 2025; Zhou et al., 2024). Other genes such as *rpoA, rpoB* and *rpoC1/C2* are the key genes involved in chloroplast function (Cho et al., 2024; Xu et al., 2024). The chloroplast genome exhibits the typical quadripartite structure, consisting of a large single-copy (LSC) region (81,798 bp), a small single-copy (SSC) region (16,671 bp), and two inverted repeat (IR) regions (25,778 bp each) (**Figure 5C**).

Comparative analysis revealed nine shared MTPTs between the chloroplast and mitogenomes, suggesting possible interorganellar gene transfer events. (**Supplementary Table S6**; **Figure 5D**). The total length of these MTPTs was 5523 bp, which accounted for 3.52% of mitogenome and 3.68% of chloroplast genome, respectively. These MTPTs range in length from 73 to 1,247 bp and have been labeled MTPT1 to MTPT9. Notably, MTPT5 and MTPT6 contained partial rRNA gene sequences, with rrn16 (plastid-derived) and rrn18 (mitochondrial homolog) respectively. In addition to rRNA fragments, four MTPTs contained complete tRNA genes: MTPT2 (*trnS-UGA*), MTPT7 (*trnW-CCA, trnP-UGG*), MTPT8 (*trnH-GUG*), and MTPT9 (*trnM-CAU*). Meanwhile, three MTPTs harbored partial plastid-derived PCGs: MTPT1 (*rbcl*), MTPT2 (*psbZ*), and MTPT4 (*psbD*). The results indicate that while tRNA transfer events were complete—potentially preserving functionality—the fragmented nature of PCGs suggests they are nonfunctional relics of historical DNA transfer.

### Variation and prediction analysis of RNA-editing sites

**Figure 6:**
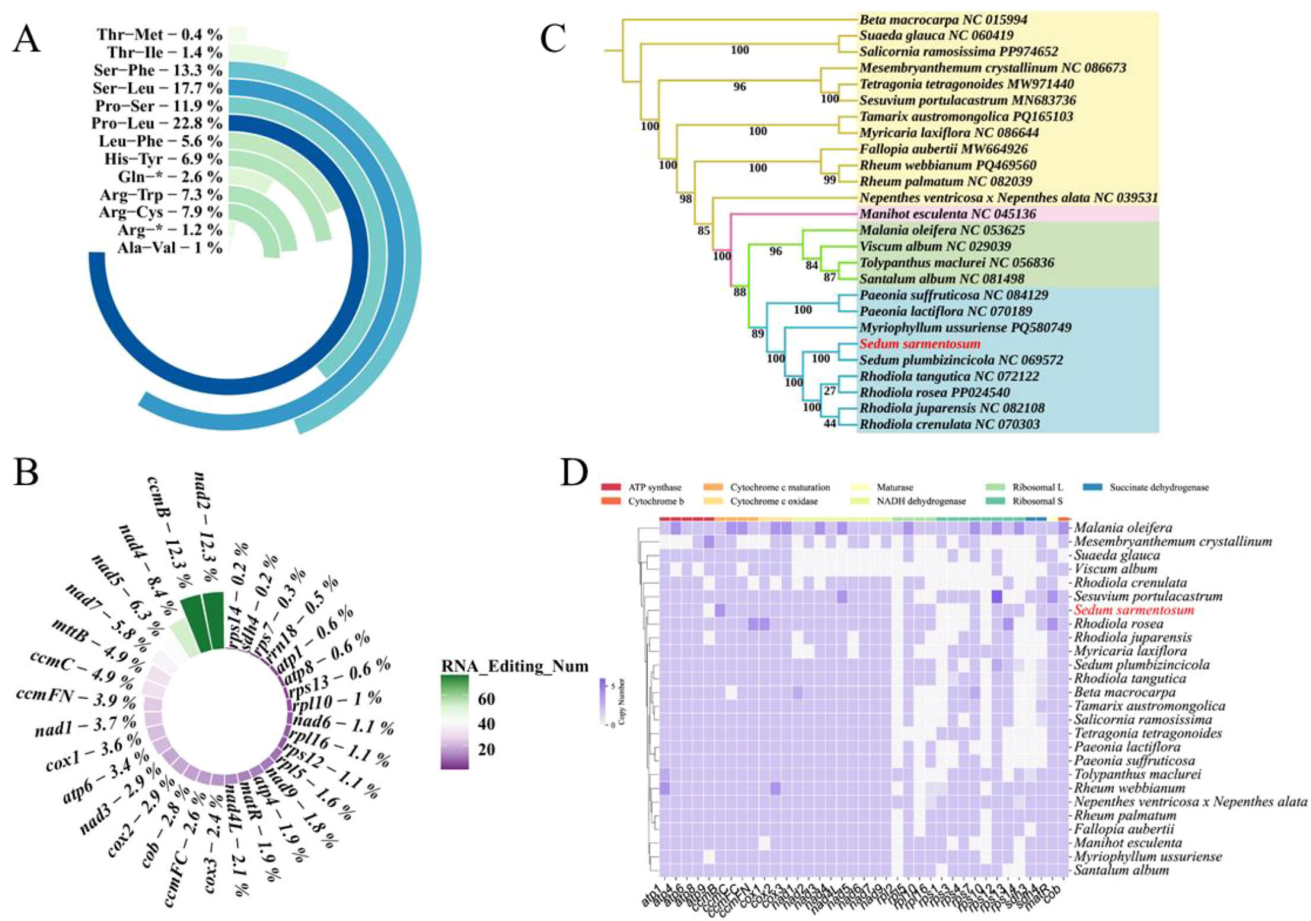
Amino acid changes, RNA editing patterns, and phylogenetic relationships based on mitochondrial PCGs. **(A)** Distribution of amino acid substitutions resulting from RNA editing in *S. sarmentosum* mitochondrial PCGs. The most frequent changes include Pro to Leu, Ser to Leu, and Ser to Phe, with varying degrees of hydrophobicity shifts. **(B)** Gene-specific counts of predicted RNA-editing events across mitochondrial PCGs in *S. sarmentosum*, with *ccmB*, *nad4*, and *ccmFN* showing the highest editing frequencies. **(C)** Maximum likelihood phylogenetic tree constructed using mitochondrial PCGs from 26 plant species, with bootstrap values shown at each node. Species are grouped by taxonomic order, highlighting the placement of *S. sarmentosum* within Crassulaceae. **(D)** Presence and absence matrix of 26 mitochondrial PCGs across related species, illustrating lineage-specific gene retention or loss. Circle size denotes the number of genes present, and color intensity reflects gene copy number.

Comprehensive analysis revealed 617 RNA-editing events in mitochondrial PCGs, of which all were C-to-U conversions (**Supplementary Table S7**; **Figure 6A, B**). Among the 24 PCGs, the *nad2 and ccmB* gene exhibited the highest number of RNA-editing sites, each 76. This was followed by the *nad4* and *nad5* genes, with 52 and 39 RNA-editing sites, respectively. Conversely, the *rps14, sdh4, rps7* and *rrn18* genes each exhibited only one, one, two and three RNA-editing events, of which the first base position account for 35.98% (222/617), the second base position account for 45.54% (281/617), the third base position account for 18.48% (114/617). Additionally, the majority of these RNA-editing events resulted in changes from hydrophilic to hydrophobic amino acids. Among these animo changes, the change from Pro to Leu exhibited the highest number, totaling 113. This was followed by the change (Ser to Leu) and Ser to Phe change, with 88 and 66 animo changes, respectively. Besides, the animo change Gln and Arg to stop codon exhibited 13 and six events.

### Evolutionary relationships among closely related species

Phylogenetic reconstruction using mitochondrial PCGs from 26 species (with *B. macrocarpa*, NC_015994, as outgroup) strongly supported a sister relationship between *S. sarmentosum* and *S. plumbizincicola* (Crassulaceae), in which the bootstrap values of the phylogenetic nodes between *S. sarmentosum* and *S. plumbizincicola* is 100 (**Figure 6C**; **Supplementary Table S8**). In addition, species of the same order are all classified together on the evolutionary tree, and there is no confusion between orders. The ML tree topology aligned with APG (Angiosperm Phylogeny Group classification) classification system (Group, T. A. P, 2016), while comparative analysis revealed lineage-specific mitochondrial PCG losses among relatives (**Figure 6D**).

### Selective pressure and nucleotide diversity of mitochondrial PCGs

**Figure 7:**
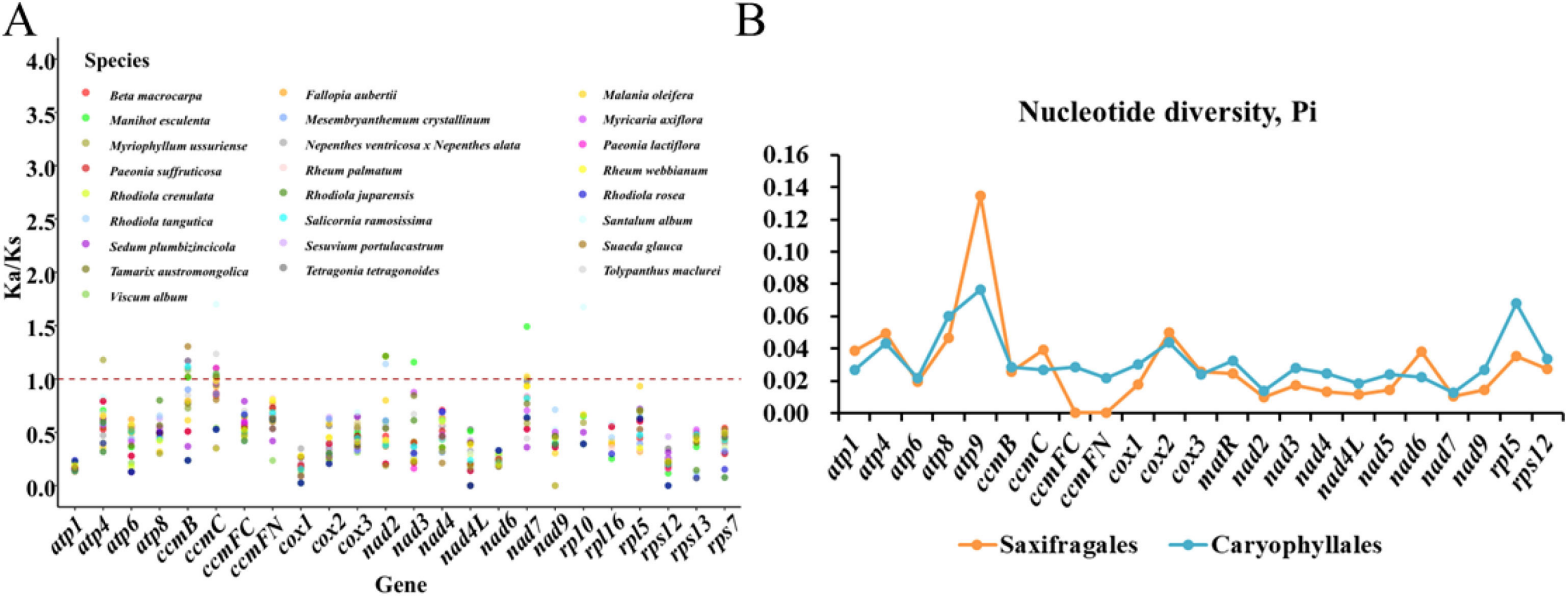
Selective pressure and nucleotide diversity analysis of mitochondrial protein-coding genes across plant lineages. **(A)** Ka/Ks ratio analysis of 24 mitochondrial protein-coding genes across 26 plant species reveals patterns of evolutionary selection. Most genes exhibit Ka/Ks values below 1, indicating purifying selection, while several genes (e.g., *ccmB, ccmC*) in specific species show Ka/Ks > 1, suggesting positive selection. **(B)** Nucleotide diversity (Pi) values of 23 genes in Saxifragales and Caryophyllales lineages. *Atp9* shows the highest variation in both orders, while genes like *ccmFC* and nad7 exhibit the lowest diversity, indicating gene-specific evolutionary constraints.

Phylogenetic analysis was performed using 25 relatives, with evolutionary selective pressures assessed through Ka/Ks ratio calculations for 24 PCGs in the *S. sarmentosum* mitogenome. The results indicated that not all of the 26 species had 24 PCGs (**Figure 7A**), such as *Viscum album, Mesembryanthemum crystallinum, S. plumbizincicola, Suaeda glauca, R. crenulata*, and so on. Among them, *Viscum album* missed 15 PCGs, followed by *Mesembryanthemum crystallinum* and *S. plumbizincicola*, with 13 and 7 missed PCGs. Of note, within the 24 PCGs of *S. plumbizincicola*, the value of Ka/Ks=0 in *nad4L* and *nad9*, while the remaining genes had Ka/Ks<1, which means these genes are more affected by negative selection during evolution, and show a conservative tendency of synonymy substitution. Additionally, *rps12* in *R. tangutica*, *nad4L* and *rps12* in *R. rosea*, *nad4L* and *rps12* in *R. juparensis* had Ka/Ks=0. In contrast, *atp4, ccmB, ccmC nad2, nad3, nad7* and *rpl10* had Ka/Ks>1 in many speices, such as *Myriophyllum ussuriense*, *Sesuvium portulacastrum, Tetragonia tetragonoides, Viscum album, B. macrocarpa, Nepenthes ventricosa x Nepenthes alata, Malania oleifera, S. glauca*, and *Santalum album*). These results indicate distinct selection patterns among the 26 examined plant species. While the majority of mitochondrial protein-coding genes exhibit strong purifying selection (Ka/Ks << 1), maintaining conserved functional domains, several genes demonstrate significant positive selection (Ka/Ks > 1). These rapidly evolving genes likely contribute to adaptive evolution in specific lineages, potentially influencing critical physiological adaptations.

To further explore the selective pressure of genes in the mitochondria of Saxifragales based on nine species and Caryophyllales based on 12 species (these two orders are closer to *S. sarmentosum*), nucleotide diversity analysis were performed based on 23 PCGs (shared genes in most 21 species). Among these 23 PCGs, the range of Pi values is from 0.00052 to 0.13483, and the gene *atp9* has the highest value both in Saxifragales and Caryophyllales (Pi=0.13483), while *ccmFC* in Saxifragales (Pi=0.00052) and *nad7* in Caryophyllales (Pi=0.01292) have the lowest (**Figure 7B**). At the same time, all of them (except *ccmFC, ccmFN* and *nad2* in Saxifragales) have a value greater than 0.01 in Saxifragales and Caryophyllales order, with a much larger variation range than those genes with polymorphism less than 0.01, reflecting the high genetic diversity and the strong adaptability and survival ability of the population.

### Secondary structures of mitochondrial tRNAs

**Figure 8:**
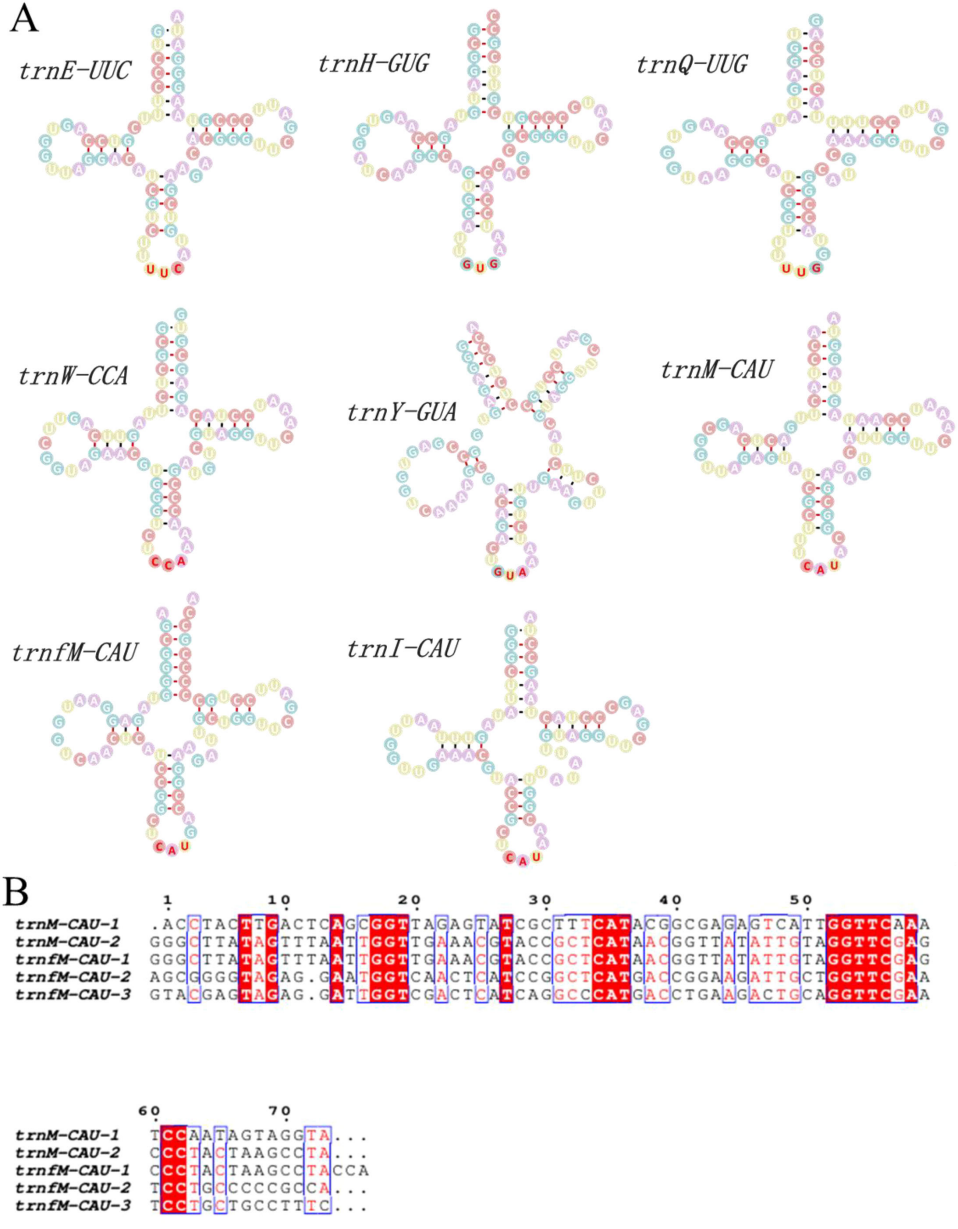
Structural and sequence analysis of mitochondrial tRNAs in *S. sarmentosum*. **(A)** Predicted secondary structures of eight mitochondrial tRNAs, including *trnE-UUC*, *trnH-GUG*, *trnQ-UUG*, *trnW-CCA*, *trnY-GUA*, *trnM-CAU*, *trnfM-CAU* and *trnI-CAU*. All exhibit canonical cloverleaf structures characteristic of functional tRNAs, with distinct loop and stem regions. **(B)** Multiple sequence alignment of five copies of the *trnM-CAU* and *trnfM-CAU* genes reveals conserved nucleotide regions and sequence variations. Red shading highlights highly conserved bases, indicating potential functional importance across gene copies.

All mitochondrial tRNAs exhibited canonical cloverleaf secondary structures, consistent with their expected functional configurations (**Figure 8A**). Each structure clearly shows the typical acceptor stem, D-loop, anticodon arm, variable loop, and TΨC loop, supporting the structural integrity necessary for proper tRNA function during mitochondrial translation. Notably, both *trnM-CAU* and *trnfM-CAU* showed genomic redundancy, with two and three native copies, respectively, identified in the mitogenomes of *S. sarmentosum* and *S. plumbizincicola* (**Figure 8B**). This copy number variation may reflect functional specialization or compensatory redundancy in mitochondrial methionine initiation or elongation processes. Multiple sequence alignment of these gene copies revealed high sequence conservation across most nucleotide positions, with several base substitutions and indels concentrated in non-anticodon regions. Red-shaded nucleotides highlight strictly conserved bases, while variable sites suggest possible lineage-specific mutations or evolutionary relaxation. The conserved anticodon motifs and stem-loop integrity across all copies imply that these duplicated tRNAs are likely functional rather than pseudogenes. The presence of multiple functional copies of methionine tRNAs may also enhance translational efficiency or reflect structural constraints associated with mitogenome evolution in Crassulaceae.

## Discussion

This study presents the successful *de novo* assembly and integrative analysis of both the mitochondrial and chloroplast genomes of *S. sarmentosum*, a representative species of Crassulaceae with important medicinal properties. By combining PacBio HiFi and Illumina sequencing technologies, we overcame the challenges posed by complex repeat structures and recombination-prone regions inherent to plant mitogenomes, generating high-contiguity, circular assemblies of both organellar genomes. These newly resolved sequences form the first complete organellar references for *S. sarmentosum*, laying a solid foundation for future research in comparative genomics, evolutionary biology, and plant systematics within Saxifragales.

This study assembled the mitogenome of *S. sarmentosum* into a circular chromosome rich in repetitive elements. This architecture aligns with findings in other Crassulaceae species (e.g., *R. tangutica, R. rosea*), which also exhibit a dynamic circular structure where conformational variation is primarily driven by repeat-mediated homologous recombination. This pattern underscores a key paradox in plant mitochondrial evolution: although the nucleotide substitution rate in angiosperm mitogenomes is remarkably low—reportedly 3–9 times lower than in plastid genomes and 15–20 times lower than in nuclear genomes—structural rearrangements occur with extraordinary speed and frequency, even among closely related lineages. Thus, mitochondrial genome rearrangement represents a highly labile process in plants, largely facilitated by recombination across repeats. This highlights the necessity of considering structural evolution alongside sequence divergence when studying organellar genome dynamics and points to the importance of identifying the nuclear-encoded factors that modulate this recombination machinery across plant lineages. (Cole, 2018 #115).

In the *S. sarmentosum* mitogenome, we identified 89 highly polymorphic SSRs in the *S. sarmentosum* mitogenome, which may be useful for species identification, genetic diversity assessment (Qi et al., 2015), comparative genomics (Behura and Severson, 2012), and linkage mapping (Miao et al., 2005). Additionally, 30 pairs of dispersed repeats were detected, which are crucial for sequence rearrangement and may influence evolution, gene expression, and phenotypic variation (Lisch, 2013). These repeats also hold potential for developing mutant populations (Veilleux et al., 2012).

Plant mitochondrial genomes frequently acquire exogenous DNA sequences through intracellular gene transfer, resulting in chimeric architectures that incorporate plastid-derived (MTPTs), nuclear-derived (NUMTs), and, in some cases, horizontally transferred sequences. This promiscuous integration of foreign DNA is a key driver of the structural complexity and evolutionary dynamism of plant mitogenomes. Among these, MTPTs are particularly common in higher plants and serve as direct evidence of ongoing evolutionary interplay between organelles (Wei et al., 2022). In *S. sarmentosum*, the mitogenome harbors 9 MTPTs, spanning 5523 bp and accounting for 3.52% of the mitogenome and 3.68% of the chloroplast genome. Their GC content (47.3%) is consistent with the neutral evolution observed in angiosperm MTPTs (Sloan and Wu, 2014; Sun et al., 2024; Wang et al., 2007; Zhu et al., 2023). Although PCGs within MTPTs are usually fragmented and nonfunctional, the frequently intact tRNA sequences may have biological significance. Emerging evidence hints at their potential roles in promoting evolutionary efficiency, possibly by supplementing mitochondrial tRNA pools or engaging in post-transcriptional RNA processing mechanisms (Wang et al., 2012). Functionally, transferred sequences—particularly intact tRNA genes—can compensate for deficits in the mitochondrial tRNA pool, thereby potentially enhancing translational capacity. Furthermore, this genetic supplementation might give rise to novel chimeric open reading frames, which could encode proteins that foster functional diversity.

The comparative analysis revealed the commonalities and specificities of MTPTs within the Sedum genus. In the plant *S. plumbizincicola*, 4 MTPTs were found, among which there was a rare fragment of 8.4 kb that maintained the complete plastid gene sequence, but its PCGs were pseudogenized due to mutations, and the tRNA genes remained intact (Ding, 2022 #116). The most significant difference between the two lies in the scale and completeness of the MTPTs: *S. sarmentosum* exhibits multiple small-scale, fragmented transfers, while *S. plumbizincicola* captures a single large-scale, structurally complete DNA fragment. This suggests that the two may be at different evolutionary time points - the transfer event of *S. plumbizincicola* may be more recent and has not yet been disrupted by genome recombination; the fragmented pattern of *S. sarmentosum* may reflect a more ancient transfer history or a more active recombination activity. However, a key common point is that both have completely retained the transferred tRNA genes. This supports an evolutionary hypothesis: regardless of the scale and timing of the transfer event, tRNA genes are more likely to be selectively retained in the mitochondrial genome of the Crassulaceae genus due to their potential functional value (such as supplementing the mitochondrial translation system). This interspecies comparison highlights the dynamic and complex nature of intracellular DNA transfer. The initial transfer mechanism, the strength of post-transfer recombination, and the selective pressure for functionality all jointly shape the significantly different MTPT profiles among different species (Wang, 2018 #117) (Warren, 2020 #118).

Furthermore, these MTPTs can act as disruptive architectural elements, providing substrates for recombination that catalyze genome rearrangements and thereby drive structural diversification of the mitochondrial genome. Over time, the accumulation of such sequences creates expansive ‘plasticity zones’, which may serve as reservoirs for evolutionary innovation. Critically, this inter-organellar gene transfer establishes a direct genetic conduit between the two energy-converting organelles, enabling a unique form of cytoplasmic coevolution. This linkage facilitates more rapid and coordinated adaptation to physiological and environmental pressures. Consequently, MTPTs should not be regarded as neutral genomic fossils but as active agents that shape mitochondrial genetics, function, and ultimately, the organism’s adaptive trajectory (Nhat Nam, 2024 #119) (Park, 2020 #120) (Allen, 2015 #121).

In vascular plants, RNA editing predominantly targets organellar transcripts, with the highest frequency observed in mitochondrial mRNAs and, to a lesser extent, chloroplast genomes. This post-transcriptional modification mechanism is mediated by nucleus-encoded PPR proteins that recognize specific cis-elements in organellar RNAs (Qiu et al., 2025), with the most common event being C-to-U transitions in mitochondrial transcripts (Edera et al., 2018; Giegé and Brennicke, 1999; Sun et al., 2020). This process plays essential roles in RNA splicing, evolutionary adaptation, and development. Given the importance of mitochondria in plant growth (He et al., 2018), extensive research has been conducted on mitochondrial RNA editing, with applications in plant breeding (Yang et al., 2022). In *S. sarmentosum*, we identified 617 C-to-U editing sites in mitochondrial PCGs. Notably, 40.12% of these edits converted hydrophilic amino acids to hydrophobic, while only 11.90% exhibited the reverse trend (**Supplementary Table S10**), indicating a net increase in protein hydrophobicity, a pattern consistent with previous findings in plant mitochondria (Kaila et al., 2019; Rehman et al., 2022; Wu and Chaw, 2022).

Generally, non-synonymous substitutions alter amino acid sequences, potentially affecting protein conformation and function. These changes may lead to adaptive advantages or disadvantages, influencing natural selection. In contrast, synonymous substitutions do not modify the protein sequence and are thus largely neutral to selection pressure. As a result, Ks reflects the background mutation rate of evolution. By comparing Ka to Ks (Ka/Ks ratio), we can infer the selective pressures acting on a gene (Song et al., 2023). Selective pressure analysis (Ka/Ks) of 26 protein-coding genes in 26 gramineous plants showed that most of the genes were affected by purification selection (Ka/Ks<1) and showed a conservative evolutionary trend. Positive selection (Ka/Ks > 1) is observed in only a limited number of mitochondrial genes across plants—such as *atp4, ccmB, ccmC, nad2, nad3, nad7*, and *rpl10*—likely reflecting adaptive responses to environmental changes. Analysis further reveals that in *S. sarmentosum*, genes associated with ATP synthase, cytochrome c biogenesis, NADH dehydrogenase, and the small ribosomal subunit exhibit stronger positive selection compared to other species. These substitutions may confer novel or enhanced protein functions, increased catalytic activity, or other adaptive advantages, thereby promoting their retention and spread over evolutionary time. Such positive selection likely drives functional adaptation in these genes, enabling organisms to better meet physiological or ecological demands—for instance, by improving stress tolerance, fine-tuning host–pathogen interactions, or facilitating species-specific evolution. Additionally, Ka/Ks > 1 indicates a relatively accelerated evolutionary rate in these genes, characterized by the accumulation of numerous non−synonymous mutations within relatively short periods. This rapid evolution may contribute to functional innovation or species differentiation, supporting the emergence of new traits or adaptive strategies (Hurst, 2002 #122) (Camps, 2007 #123). Although synonymous substitutions are generally more common in plant mitochondrial genomes, purifying selection strongly constrains variation in these essential genes (Mower et al., 2007). Comparative assessment of selection pressures through Ka/Ks ratios thus helps uncover functional divergence among orthologous protein−coding genes across taxa, clarifies evolutionary trajectories, and can contribute to the construction of more accurate phylogenetic frameworks (Li, 2023 #124).

Phylogenetic analysis of the complete mitogenomes of *S. sarmentosum* and related Crassulaceae species provides key insights into their genetic diversity and evolutionary relationships. Although *S. sarmentosum* shows close phylogenetic ties to other Crassulaceae members, its distinct genomic features—likely shaped by geographic isolation, ecological adaptation, and genetic drift—highlight its evolutionary divergence (Chen et al., 2019; Liu et al., 2021). These findings, combined with its unique cpDNA characteristics, enhance our understanding of speciation and genomic evolution in this family. This comparative genomic approach not only clarifies *S. sarmentosum*’s genetic distinctiveness but also sheds light on broader evolutionary dynamics within Crassulaceae. By revealing the interplay of genetic and environmental factors in diversification, the study advances plant genomics research. Furthermore, it establishes a foundation for future investigations into genome evolution, adaptive mechanisms, and potential applications in conservation, cultivation, and biotechnology.

In conclusion, the observed genomic divergence between *S. sarmentosum* and relatives highlights the combined influence of evolutionary dynamics, ecological adaptation, and habitat-specific selection pressures in shaping their genetic differentiation. These findings provide valuable insights into the mechanisms shaping genetic diversity in the Crassulaceae family. By elucidating interspecific genetic relationships and adaptations, this study establishes a framework for future research on their evolutionary pathways and ecological specialization.

## Data Availability

The datasets presented in this study can be found in online repositories. The names of the repository/repositories and accession number(s) can be found in the article/**Supplementary Material**.
