# Supplementary material for: Characterization and evolutionary insights into complete mitochondrial genome of Sedum sarmentosum within the family Crassulaceae
Source: Front Plant Sci. 2026 Feb 6;17:1710625. doi: 10.3389/fpls.2026.1710625 (PMC12920544; doi:10.3389/fpls.2026.1710625)
Supplement: Supplementary file 1 [file Table1.docx]

**Table S1 | Application of DNA Barcoding of *rbcl* in *Sedum sarmentosum*.**

| **PID [BIN]** | **Phylum** | **Class** | **Order** | **Family** | **Subfamily** | **Genus** | **Species** | **Indels** | **ID%** |
| --- | --- | --- | --- | --- | --- | --- | --- | --- | --- |
| GBVJ1727-11 | Tracheophyta | Magnoliopsida | Saxifragales | Crassulaceae | Sempervivoideae | Sedum | *Sedum sarmentosum* | 4 | 99.64 |
| GBVJ1728-11 | Tracheophyta | Magnoliopsida | Saxifragales | Crassulaceae | Sempervivoideae | Sedum | *Sedum sarmentosum* | 4 | 99.64 |
| GBVJ1703-11 | Tracheophyta | Magnoliopsida | Saxifragales | Crassulaceae | Sempervivoideae | Sedum | *Sedum emarginatum* | 4 | 99.27 |
| GBGHM599-24 | Tracheophyta | Magnoliopsida | Saxifragales | Crassulaceae | Sempervivoideae | Sedum | *Sedum bulbiferum* | 0 | 99.24 |
| GRDTX020-21 | Tracheophyta | Magnoliopsida | Saxifragales | Crassulaceae | Sempervivoideae | Sedum | *Sedum cockerellii* | 2 | 99.07 |
| GBVS1038-13 | Tracheophyta | Magnoliopsida | Saxifragales | Crassulaceae | Sempervivoideae | Sedum | *Sedum sexangulare* | 0 | 99.03 |
| GBVS1039-13 | Tracheophyta | Magnoliopsida | Saxifragales | Crassulaceae | Sempervivoideae | Sedum | *Sedum sexangulare* | 0 | 99.03 |
| GBVS1040-13 | Tracheophyta | Magnoliopsida | Saxifragales | Crassulaceae | Sempervivoideae | Sedum | *Sedum sexangulare* | 0 | 99.03 |
| GBVP1961-14 | Tracheophyta | Magnoliopsida | Saxifragales | Crassulaceae | Echeverioideae | Echeveria | *Echeveria elegans* | 2 | 98.72 |
| GBVX5712-15 | Tracheophyta | Magnoliopsida | Saxifragales | Crassulaceae | Sempervivoideae | Sedum | *Sedum anglicum* | 2 | 98.72 |
| GBVJ1724-11 | Tracheophyta | Magnoliopsida | Saxifragales | Crassulaceae | Sempervivoideae | Sedum | *Sedum rubrotinctum* | 2 | 98.72 |
| FPUK613-14 | Tracheophyta | Magnoliopsida | Saxifragales | Crassulaceae | Sempervivoideae | Sedum | *Sedum acre* | 4 | 98.68 |
| POWNA1504-12 | Tracheophyta | Magnoliopsida | Saxifragales | Crassulaceae | Sempervivoideae | Sedum | *Sedum acre* | 4 | 98.68 |
| POWNA2778-12 | Tracheophyta | Magnoliopsida | Saxifragales | Crassulaceae | Sempervivoideae | Sedum | *Sedum acre* | 4 | 98.68 |
| POWNA668-10 | Tracheophyta | Magnoliopsida | Saxifragales | Crassulaceae | Sempervivoideae | Sedum | *Sedum acre* | 4 | 98.68 |
| FPUK173-14 | Tracheophyta | Magnoliopsida | Saxifragales | Crassulaceae | Sempervivoideae | Sedum | *Sedum anglicum* | 2 | 98.67 |
| POWNA1779-12 | Tracheophyta | Magnoliopsida | Saxifragales | Crassulaceae | Sempervivoideae | Sedum | *Sedum anglicum* | 2 | 98.67 |
| POWNA669-10 | Tracheophyta | Magnoliopsida | Saxifragales | Crassulaceae | Sempervivoideae | Sedum | *Sedum anglicum* | 2 | 98.67 |
| POWNA1510-12 | Tracheophyta | Magnoliopsida | Saxifragales | Crassulaceae | Sempervivoideae | Sedum | *Sedum acre* | 4 | 98.66 |
| PNG182-18 | Tracheophyta | Magnoliopsida | Saxifragales | Crassulaceae |  | Kalanchoe | *Kalanchoe lanceolata* | 4 | 98.52 |
| PNG225-18 | Tracheophyta | Magnoliopsida | Saxifragales | Crassulaceae |  | Kalanchoe | *Kalanchoe lanceolata* | 4 | 98.52 |
| EDTOL723-21 | Tracheophyta | Magnoliopsida | Saxifragales | Crassulaceae | Sempervivoideae | Sedum | *Sedum anglicum* | 0 | 98.47 |
| EDTOL1342-23 | Tracheophyta | Magnoliopsida | Saxifragales | Crassulaceae | Sempervivoideae | Sedum | *Sedum acre* | 4 | 98.37 |
| GBVJ1713-11 | Tracheophyta | Magnoliopsida | Saxifragales | Crassulaceae | Sempervivoideae | Sedum | *Sedum lineare* | 4 | 98.37 |
| GENG341-14 | Tracheophyta | Magnoliopsida | Saxifragales | Crassulaceae |  | Bryophyllum | *Bryophyllum pinnatum* | 4 | 98.36 |
| TRIOP423-13 | Tracheophyta | Magnoliopsida | Saxifragales | Crassulaceae |  | Jovibarba |  | 4 | 98.34 |
| TRIOP424-13 | Tracheophyta | Magnoliopsida | Saxifragales | Crassulaceae |  | Jovibarba |  | 4 | 98.34 |
| TRIOP425-13 | Tracheophyta | Magnoliopsida | Saxifragales | Crassulaceae |  | Jovibarba |  | 4 | 98.34 |
| TRIOP426-13 | Tracheophyta | Magnoliopsida | Saxifragales | Crassulaceae |  | Jovibarba |  | 4 | 98.34 |
| GRDTX268-21 | Tracheophyta | Magnoliopsida | Saxifragales | Crassulaceae | Sedoideae | Lenophyllum | *Lenophyllum texanum* | 2 | 98.32 |
| GENG498-14 | Tracheophyta | Magnoliopsida | Saxifragales | Crassulaceae |  | Bryophyllum |  | 1 | 98.30 |
| GENG529-14 | Tracheophyta | Magnoliopsida | Saxifragales | Crassulaceae |  | Kalanchoe | *Kalanchoe pinnata* | 1 | 98.29 |
| GENG128-13 | Tracheophyta | Magnoliopsida | Saxifragales | Crassulaceae |  | Kalanchoe | *Kalanchoe pinnata* | 0 | 98.28 |
| GBVJ1579-11 | Tracheophyta | Magnoliopsida | Saxifragales | Crassulaceae |  | Cotyledon | *Cotyledon orbiculata* | 4 | 98.19 |
| GBVJ1636-11 | Tracheophyta | Magnoliopsida | Saxifragales | Crassulaceae |  | Kalanchoe | *Kalanchoe daigremontiana* | 4 | 98.19 |
| GBVJ1637-11 | Tracheophyta | Magnoliopsida | Saxifragales | Crassulaceae |  | Kalanchoe | *Kalanchoe delagoensis* | 2 | 98.15 |
| GRDMO096-21 | Tracheophyta | Magnoliopsida | Saxifragales | Crassulaceae |  | Kalanchoe | *Kalanchoe sp.* | 4 | 98.15 |
| GRDMO311-21 | Tracheophyta | Magnoliopsida | Saxifragales | Crassulaceae | Echeverioideae | Dudleya | *Dudleya farinosa* | 4 | 97.97 |
| GBVY3222-14 | Tracheophyta | Magnoliopsida | Saxifragales | Crassulaceae | Sempervivoideae | Sedum | *Sedum album* | 4 | 97.96 |
| POWNA1508-12 | Tracheophyta | Magnoliopsida | Saxifragales | Crassulaceae | Sempervivoideae | Sedum | *Sedum album* | 4 | 97.93 |
| POWNB107-10 | Tracheophyta | Magnoliopsida | Saxifragales | Crassulaceae | Sempervivoideae | Sedum | *Sedum album* | 4 | 97.93 |
| POWNA1629-12 | Tracheophyta | Magnoliopsida | Saxifragales | Crassulaceae |  | Umbilicus | *Umbilicus rupestris* | 4 | 97.93 |
| POWNA1633-12 | Tracheophyta | Magnoliopsida | Saxifragales | Crassulaceae |  | Umbilicus | *Umbilicus rupestris* | 4 | 97.93 |
| POWNA1634-12 | Tracheophyta | Magnoliopsida | Saxifragales | Crassulaceae |  | Umbilicus | *Umbilicus rupestris* | 4 | 97.93 |
| POWNA2423-12 | Tracheophyta | Magnoliopsida | Saxifragales | Crassulaceae |  | Umbilicus | *Umbilicus rupestris* | 4 | 97.93 |
| POWNB029-10 | Tracheophyta | Magnoliopsida | Saxifragales | Crassulaceae |  | Umbilicus | *Umbilicus rupestris* | 4 | 97.93 |
| GBVP6570-15 | Tracheophyta | Magnoliopsida | Saxifragales | Crassulaceae |  | Rhodiola | *Rhodiola alsia* | 0 | 97.86 |
| GBVP6807-15 | Tracheophyta | Magnoliopsida | Saxifragales | Crassulaceae |  | Rhodiola | *Rhodiola coccinea* | 0 | 97.86 |
| GBVP6808-15 | Tracheophyta | Magnoliopsida | Saxifragales | Crassulaceae |  | Rhodiola | *Rhodiola coccinea* | 0 | 97.86 |
| GBVP6586-15 | Tracheophyta | Magnoliopsida | Saxifragales | Crassulaceae |  | Rhodiola | *Rhodiola coccinea subsp. scabrida* | 0 | 97.86 |
| GBVP6699-15 | Tracheophyta | Magnoliopsida | Saxifragales | Crassulaceae |  | Rhodiola | *Rhodiola dumulosa* | 0 | 97.86 |
| GBVP6812-15 | Tracheophyta | Magnoliopsida | Saxifragales | Crassulaceae |  | Rhodiola | *Rhodiola fastigiata* | 0 | 97.86 |
| GBVP6813-15 | Tracheophyta | Magnoliopsida | Saxifragales | Crassulaceae |  | Rhodiola | *Rhodiola fastigiata* | 0 | 97.86 |
| GBVP6595-15 | Tracheophyta | Magnoliopsida | Saxifragales | Crassulaceae |  | Rhodiola | *Rhodiola gannanica* | 0 | 97.86 |
| GBVP6818-15 | Tracheophyta | Magnoliopsida | Saxifragales | Crassulaceae |  | Rhodiola | *Rhodiola kirilowii* | 0 | 97.86 |
| GBVP6702-15 | Tracheophyta | Magnoliopsida | Saxifragales | Crassulaceae |  | Rhodiola | *Rhodiola rosea* | 0 | 97.86 |
| GBVP6603-15 | Tracheophyta | Magnoliopsida | Saxifragales | Crassulaceae |  | Rhodiola | *Rhodiola smithii* | 0 | 97.86 |
| GBVP6604-15 | Tracheophyta | Magnoliopsida | Saxifragales | Crassulaceae |  | Rhodiola | *Rhodiola tangutica* | 0 | 97.86 |
| EDTOL1299-23 | Tracheophyta | Magnoliopsida | Saxifragales | Crassulaceae | Sempervivoideae | Sedum | *Sedum album* | 4 | 97.82 |
| GBVX5519-15 | Tracheophyta | Magnoliopsida | Saxifragales | Crassulaceae |  | Sempervivum | *Sempervivum tectorum* | 4 | 97.82 |
| GBVX5644-15 | Tracheophyta | Magnoliopsida | Saxifragales | Crassulaceae |  | Sempervivum | *Sempervivum tectorum* | 4 | 97.82 |
| EDTOL736-21 | Tracheophyta | Magnoliopsida | Saxifragales | Crassulaceae |  | Umbilicus | *Umbilicus rupestris* | 4 | 97.82 |
| GBVP6809-15 | Tracheophyta | Magnoliopsida | Saxifragales | Crassulaceae |  | Rhodiola | *Rhodiola crenulata* | 0 | 97.67 |
| GBVP6865-15 | Tracheophyta | Magnoliopsida | Saxifragales | Crassulaceae |  | Rhodiola | *Rhodiola rosea* | 0 | 97.67 |
| GBVP6822-15 | Tracheophyta | Magnoliopsida | Saxifragales | Crassulaceae |  | Rhodiola | *Rhodiola yunnanensis* | 0 | 97.67 |
| GBVP6823-15 | Tracheophyta | Magnoliopsida | Saxifragales | Crassulaceae |  | Rhodiola | *Rhodiola yunnanensis* | 0 | 97.67 |
| GBVP6814-15 | Tracheophyta | Magnoliopsida | Saxifragales | Crassulaceae |  | Rhodiola | *Rhodiola fastigiata* | 0 | 97.66 |
| GBVP6816-15 | Tracheophyta | Magnoliopsida | Saxifragales | Crassulaceae |  | Rhodiola | *Rhodiola heterodonta* | 0 | 97.66 |
| GBVP6958-15 | Tracheophyta | Magnoliopsida | Saxifragales | Crassulaceae |  | Rhodiola | *Rhodiola rosea* | 0 | 97.66 |
| GBVP6703-15 | Tracheophyta | Magnoliopsida | Saxifragales | Crassulaceae |  | Rhodiola | *Rhodiola sachalinensis* | 0 | 97.66 |
| GBVY2319-14 | Tracheophyta | Magnoliopsida | Saxifragales | Crassulaceae |  | Greenovia | *Greenovia aurea* | 4 | 97.64 |
| GBVY2321-14 | Tracheophyta | Magnoliopsida | Saxifragales | Crassulaceae |  | Greenovia | *Greenovia diplocycla* | 4 | 97.64 |
| GBVY2322-14 | Tracheophyta | Magnoliopsida | Saxifragales | Crassulaceae |  | Greenovia | *Greenovia diplocycla* | 4 | 97.64 |
| GBVY2324-14 | Tracheophyta | Magnoliopsida | Saxifragales | Crassulaceae |  | Greenovia | *Greenovia diplocycla* | 4 | 97.64 |
| GBVY2325-14 | Tracheophyta | Magnoliopsida | Saxifragales | Crassulaceae |  | Greenovia | *Greenovia diplocycla* | 4 | 97.64 |
| EDTOL1858-24 | Tracheophyta | Magnoliopsida | Saxifragales | Crassulaceae | Sempervivoideae | Petrosedum | *Petrosedum forsterianum* | 4 | 97.64 |
| EDTOL1077-22 | Tracheophyta | Magnoliopsida | Saxifragales | Crassulaceae |  | Rhodiola | *Rhodiola rosea* | 4 | 97.64 |
| GBVJ1749-11 | Tracheophyta | Magnoliopsida | Saxifragales | Crassulaceae |  | Umbilicus | *Umbilicus horizontalis* | 2 | 97.60 |
| GRDMO172-21 | Tracheophyta | Magnoliopsida | Saxifragales | Crassulaceae |  | Kalanchoe | *Kalanchoe beharensis* | 4 | 97.60 |
| GBVX5631-15 | Tracheophyta | Magnoliopsida | Saxifragales | Crassulaceae | Sempervivoideae | Sedum | *Sedum forsterianum* | 0 | 97.55 |
| FPUK172-14 | Tracheophyta | Magnoliopsida | Saxifragales | Crassulaceae | Sempervivoideae | Petrosedum | *Petrosedum forsterianum* | 4 | 97.55 |
| FPUK280-14 | Tracheophyta | Magnoliopsida | Saxifragales | Crassulaceae | Sempervivoideae | Petrosedum | *Petrosedum forsterianum* | 4 | 97.55 |
| POWNA1630-12 | Tracheophyta | Magnoliopsida | Saxifragales | Crassulaceae | Sempervivoideae | Petrosedum | *Petrosedum forsterianum* | 4 | 97.55 |
| POWNA1631-12 | Tracheophyta | Magnoliopsida | Saxifragales | Crassulaceae | Sempervivoideae | Petrosedum | *Petrosedum forsterianum* | 4 | 97.55 |
| POWNA670-10 | Tracheophyta | Magnoliopsida | Saxifragales | Crassulaceae | Sempervivoideae | Petrosedum | *Petrosedum forsterianum* | 4 | 97.55 |
| POWNA671-10 | Tracheophyta | Magnoliopsida | Saxifragales | Crassulaceae |  | Rhodiola | *Rhodiola rosea* | 4 | 97.55 |
| GRDMO433-21 | Tracheophyta | Magnoliopsida | Saxifragales | Crassulaceae |  | Aeonium | *Aeonium percarneum* | 4 | 97.42 |
| YNPBP150-21 | Tracheophyta | Magnoliopsida | Saxifragales | Crassulaceae | Sempervivoideae | Sedum | *Sedum lanceolatum* | 4 | 97.42 |
| YNPBP151-21 | Tracheophyta | Magnoliopsida | Saxifragales | Crassulaceae | Sempervivoideae | Sedum | *Sedum lanceolatum* | 4 | 97.42 |
| YNPBP152-21 | Tracheophyta | Magnoliopsida | Saxifragales | Crassulaceae | Sempervivoideae | Sedum | *Sedum lanceolatum* | 4 | 97.42 |
| POWNA1632-12 | Tracheophyta | Magnoliopsida | Saxifragales | Crassulaceae |  | Rhodiola | *Rhodiola rosea* | 4 | 97.36 |
| FPUK281-14 | Tracheophyta | Magnoliopsida | Saxifragales | Crassulaceae | Sempervivoideae | Sedum | *Sedum villosum* | 2 | 97.34 |
| FPUK282-14 | Tracheophyta | Magnoliopsida | Saxifragales | Crassulaceae | Sempervivoideae | Sedum | *Sedum villosum* | 2 | 97.34 |
| FPUK293-14 | Tracheophyta | Magnoliopsida | Saxifragales | Crassulaceae | Sempervivoideae | Sedum | *Sedum villosum* | 2 | 97.34 |
| FPUK453-14 | Tracheophyta | Magnoliopsida | Saxifragales | Crassulaceae | Sempervivoideae | Sedum | *Sedum villosum* | 2 | 97.34 |
| YNPBP487-21 | Tracheophyta | Magnoliopsida | Saxifragales | Crassulaceae | Sempervivoideae | Sedum | *Sedum lanceolatum* | 0 | 97.31 |
| GBVW2823-13 | Tracheophyta | Magnoliopsida | Saxifragales | Crassulaceae | Sempervivoideae | Sedum | *Sedum lanceolatum* | 4 | 97.28 |
| EDTOL1967-24 | Tracheophyta | Magnoliopsida | Saxifragales | Crassulaceae | Sempervivoideae | Sedum | *Sedum villosum* | 2 | 97.25 |
| GBVJ1578-11 | Tracheophyta | Magnoliopsida | Saxifragales | Crassulaceae |  | Aichryson | *Aichryson villosum* | 2 | 97.23 |
| GBVJ1597-11 | Tracheophyta | Magnoliopsida | Saxifragales | Crassulaceae |  | Crassula | *Crassula multicava* | 2 | 96.49 |
