# Supplementary material for: Characterization and evolutionary insights into complete mitochondrial genome of Sedum sarmentosum within the family Crassulaceae
Source: Front Plant Sci. 2026 Feb 6;17:1710625. doi: 10.3389/fpls.2026.1710625 (PMC12920544; doi:10.3389/fpls.2026.1710625)
Supplement: Supplementary file 2 [file Table2.docx]

**Table S2 | Application of DNA Barcoding of *matK* in *Sedum sarmentosum*.**

| **PID [BIN]** | **Phylum** | **Class** | **Order** | **Family** | **Subfamily** | **Genus** | **Species** | **Indels** | **ID%** |
| --- | --- | --- | --- | --- | --- | --- | --- | --- | --- |
| GBVJ1726-11 | Tracheophyta | Magnoliopsida | Saxifragales | Crassulaceae | Sempervivoideae | Sedum | *Sedum sarmentosum* | 13 | 98.53 |
| GBVJ1720-11 | Tracheophyta | Magnoliopsida | Saxifragales | Crassulaceae | Sempervivoideae | Sedum | *Sedum oaxacanum* | 2 | 97.48 |
| GBVJ1742-11 | Tracheophyta | Magnoliopsida | Saxifragales | Crassulaceae |  | Tacitus | *Tacitus bellus* | 0 | 97.32 |
| GBVJ1704-11 | Tracheophyta | Magnoliopsida | Saxifragales | Crassulaceae | Sempervivoideae | Sedum | *Sedum farinosum* | 1 | 97.14 |
| GBVJ1717-11 | Tracheophyta | Magnoliopsida | Saxifragales | Crassulaceae | Sempervivoideae | Sedum | *Sedum morrisonense* | 2 | 95.58 |
| GBVJ1732-11 | Tracheophyta | Magnoliopsida | Saxifragales | Crassulaceae | Sempervivoideae | Sedum | *Sedum urvillei* | 0 | 95.55 |
| GBVJ1695-11 | Tracheophyta | Magnoliopsida | Saxifragales | Crassulaceae | Sempervivoideae | Sedum | *Sedum alfredii* | 0 | 95.49 |
| GBVJ1692-11 | Tracheophyta | Magnoliopsida | Saxifragales | Crassulaceae | Sempervivoideae | Sedum | *Sedum alfredii* | 3 | 95.47 |
| GBVJ1696-11 | Tracheophyta | Magnoliopsida | Saxifragales | Crassulaceae | Sempervivoideae | Sedum | *Sedum alfredii* | 3 | 95.34 |
| GBVJ1688-11 | Tracheophyta | Magnoliopsida | Saxifragales | Crassulaceae | Sempervivoideae | Sedum | *Sedum alfredii* | 0 | 94.83 |
| GBVJ1689-11 | Tracheophyta | Magnoliopsida | Saxifragales | Crassulaceae | Sempervivoideae | Sedum | *Sedum alfredii* | 0 | 94.83 |
| GBVJ1698-11 | Tracheophyta | Magnoliopsida | Saxifragales | Crassulaceae | Sempervivoideae | Sedum | *Sedum bulbiferum* | 0 | 94.68 |
| GBVJ1718-11 | Tracheophyta | Magnoliopsida | Saxifragales | Crassulaceae | Sempervivoideae | Sedum | *Sedum multiceps* | 0 | 94.68 |
| GBVJ1694-11 | Tracheophyta | Magnoliopsida | Saxifragales | Crassulaceae | Sempervivoideae | Sedum | *Sedum alfredii* | 0 | 94.56 |
| GBVJ1697-11 | Tracheophyta | Magnoliopsida | Saxifragales | Crassulaceae | Sempervivoideae | Sedum | *Sedum baileyi* | 1 | 94.56 |
| GBVJ1693-11 | Tracheophyta | Magnoliopsida | Saxifragales | Crassulaceae | Sempervivoideae | Sedum | *Sedum alfredii* | 0 | 94.43 |
| NNOR1921-16 | Tracheophyta | Magnoliopsida | Saxifragales | Crassulaceae | Sempervivoideae | Sedum | *Sedum annuum* | 0 | 94.29 |
| PNOR1667-18 | Tracheophyta | Magnoliopsida | Saxifragales | Crassulaceae | Sempervivoideae | Sedum | *Sedum annuum* | 0 | 94.29 |
| GBVJ1691-11 | Tracheophyta | Magnoliopsida | Saxifragales | Crassulaceae | Sempervivoideae | Sedum | *Sedum alfredii* | 1 | 94.29 |
| MKCRA043-08 | Tracheophyta | Magnoliopsida | Saxifragales | Crassulaceae | Echeverioideae | Echeveria | *Echeveria kimnachii* | 0 | 94.12 |
| MKCRA092-08 | Tracheophyta | Magnoliopsida | Saxifragales | Crassulaceae | Echeverioideae | Thompsonella | *Thompsonella spathulata* | 0 | 94.12 |
| LEDCL027-17 | Tracheophyta | Magnoliopsida | Saxifragales | Crassulaceae | Echeverioideae | Echeveria | *Echeveria lilacina* | 0 | 94.01 |
| LEDCL036-17 | Tracheophyta | Magnoliopsida | Saxifragales | Crassulaceae | Echeverioideae | Echeveria | *Echeveria munizii* | 0 | 94.01 |
| LEDCL039-17 | Tracheophyta | Magnoliopsida | Saxifragales | Crassulaceae | Echeverioideae | Echeveria | *Echeveria novogaliciana* | 0 | 94.01 |
| LEDCL075-17 | Tracheophyta | Magnoliopsida | Saxifragales | Crassulaceae | Echeverioideae | Echeveria | *Echeveria sp.* | 0 | 94.01 |
| MKCRA070-08 | Tracheophyta | Magnoliopsida | Saxifragales | Crassulaceae | Echeverioideae | Pachyphytum | *Pachyphytum machucae* | 0 | 94.01 |
| LEDCL011-17 | Tracheophyta | Magnoliopsida | Saxifragales | Crassulaceae | Echeverioideae | Echeveria | *Echeveria chihuahuensis* | 0 | 94.00 |
| LEDCL051-17 | Tracheophyta | Magnoliopsida | Saxifragales | Crassulaceae | Echeverioideae | Echeveria | *Echeveria rulfiana* | 0 | 94.00 |
| MKCRA063-08 | Tracheophyta | Magnoliopsida | Saxifragales | Crassulaceae | Echeverioideae | Pachyphytum | *Pachyphytum longifolium* | 0 | 94.00 |
| LEDCL059-17 | Tracheophyta | Magnoliopsida | Saxifragales | Crassulaceae | Echeverioideae | Echeveria | *Echeveria sp.* | 0 | 93.99 |
| MKCRA058-08 | Tracheophyta | Magnoliopsida | Saxifragales | Crassulaceae | Echeverioideae | Pachyphytum | *Pachyphytum bracteosum* | 0 | 93.99 |
| MKCRA059-08 | Tracheophyta | Magnoliopsida | Saxifragales | Crassulaceae | Echeverioideae | Pachyphytum | *Pachyphytum caesium* | 0 | 93.99 |
| MKCRA072-08 | Tracheophyta | Magnoliopsida | Saxifragales | Crassulaceae | Echeverioideae | Pachyphytum | *Pachyphytum fittkaui* | 0 | 93.99 |
| MKCRA067-08 | Tracheophyta | Magnoliopsida | Saxifragales | Crassulaceae | Echeverioideae | Pachyphytum | *Pachyphytum rzedowskii* | 0 | 93.99 |
| MKCRA068-08 | Tracheophyta | Magnoliopsida | Saxifragales | Crassulaceae | Echeverioideae | Pachyphytum | *Pachyphytum sp.* | 0 | 93.99 |
| MKCRA061-08 | Tracheophyta | Magnoliopsida | Saxifragales | Crassulaceae | Echeverioideae | Pachyphytum | *Pachyphytum werdermannii* | 0 | 93.99 |
| LEDCL076-17 | Tracheophyta | Magnoliopsida | Saxifragales | Crassulaceae | Echeverioideae | Echeveria | *Echeveria sp.* | 0 | 93.96 |
| MKCRA066-08 | Tracheophyta | Magnoliopsida | Saxifragales | Crassulaceae | Echeverioideae | Pachyphytum | *Pachyphytum garciae* | 0 | 93.96 |
| LEDCL047-17 | Tracheophyta | Magnoliopsida | Saxifragales | Crassulaceae | Echeverioideae | Echeveria | *Echeveria purhepecha* | 0 | 93.95 |
| LEDCL001-17 | Tracheophyta | Magnoliopsida | Saxifragales | Crassulaceae | Echeverioideae | Echeveria | *Echeveria acutifolia* | 0 | 93.88 |
| LEDCL004-17 | Tracheophyta | Magnoliopsida | Saxifragales | Crassulaceae | Echeverioideae | Echeveria | *Echeveria fimbriata* | 0 | 93.88 |
| LEDCL031-17 | Tracheophyta | Magnoliopsida | Saxifragales | Crassulaceae | Echeverioideae | Echeveria | *Echeveria marianae* | 0 | 93.88 |
| LEDCL041-17 | Tracheophyta | Magnoliopsida | Saxifragales | Crassulaceae | Echeverioideae | Echeveria | *Echeveria patriotica* | 0 | 93.88 |
| LEDCL078-17 | Tracheophyta | Magnoliopsida | Saxifragales | Crassulaceae | Echeverioideae | Echeveria | *Echeveria sp.* | 0 | 93.88 |
| LEDCL086-17 | Tracheophyta | Magnoliopsida | Saxifragales | Crassulaceae | Echeverioideae | Echeveria | *Echeveria tencho* | 0 | 93.88 |
| MKCRA069-08 | Tracheophyta | Magnoliopsida | Saxifragales | Crassulaceae | Echeverioideae | Pachyphytum | *Pachyphytum brachetii* | 0 | 93.87 |
| MKCRA073-08 | Tracheophyta | Magnoliopsida | Saxifragales | Crassulaceae | Echeverioideae | Pachyphytum | *Pachyphytum brevifolium* | 0 | 93.87 |
| LEDCL014-17 | Tracheophyta | Magnoliopsida | Saxifragales | Crassulaceae | Echeverioideae | Echeveria | *Echeveria cuspidata var. zaragozae* | 0 | 93.86 |
| LEDCL016-17 | Tracheophyta | Magnoliopsida | Saxifragales | Crassulaceae | Echeverioideae | Echeveria | *Echeveria diffractens* | 0 | 93.75 |
| LEDCL040-17 | Tracheophyta | Magnoliopsida | Saxifragales | Crassulaceae | Echeverioideae | Echeveria | *Echeveria olivacea* | 0 | 93.75 |
| LEDCL063-17 | Tracheophyta | Magnoliopsida | Saxifragales | Crassulaceae | Echeverioideae | Echeveria | *Echeveria sp.* | 0 | 93.75 |
| LEDCL088-17 | Tracheophyta | Magnoliopsida | Saxifragales | Crassulaceae | Echeverioideae | Echeveria | *Echeveria tolimanensis* | 0 | 93.75 |
| LEDCL089-17 | Tracheophyta | Magnoliopsida | Saxifragales | Crassulaceae | Echeverioideae | Echeveria | *Echeveria trianthina* | 0 | 93.75 |
| LEDCL092-17 | Tracheophyta | Magnoliopsida | Saxifragales | Crassulaceae | Echeverioideae | Echeveria | *Echeveria unguiculata* | 0 | 93.75 |
| MKCRA080-08 | Tracheophyta | Magnoliopsida | Saxifragales | Crassulaceae | Echeverioideae | Cremnophila | *Cremnophila sp.* | 0 | 93.74 |
| MKCRA044-08 | Tracheophyta | Magnoliopsida | Saxifragales | Crassulaceae | Echeverioideae | Echeveria | *Echeveria amoena* | 0 | 93.74 |
| MKCRA021-08 | Tracheophyta | Magnoliopsida | Saxifragales | Crassulaceae | Echeverioideae | Echeveria | *Echeveria mucronata* | 0 | 93.74 |
| MKCRA012-08 | Tracheophyta | Magnoliopsida | Saxifragales | Crassulaceae | Echeverioideae | Echeveria | *Echeveria penduliflora* | 0 | 93.74 |
| MKCRA032-08 | Tracheophyta | Magnoliopsida | Saxifragales | Crassulaceae | Echeverioideae | Echeveria | *Echeveria heterosepala* | 0 | 93.73 |
| MKCRA071-08 | Tracheophyta | Magnoliopsida | Saxifragales | Crassulaceae | Echeverioideae | Pachyphytum | *Pachyphytum glutinicaule* | 0 | 93.73 |
| MKCRA093-08 | Tracheophyta | Magnoliopsida | Saxifragales | Crassulaceae | Echeverioideae | Thompsonella | *Thompsonella mixtecana* | 0 | 93.73 |
| MKCRA036-08 | Tracheophyta | Magnoliopsida | Saxifragales | Crassulaceae | Echeverioideae | Echeveria | *Echeveria bifida* | 0 | 93.62 |
| MKCRA030-08 | Tracheophyta | Magnoliopsida | Saxifragales | Crassulaceae | Echeverioideae | Echeveria | *Echeveria calderoniae* | 0 | 93.62 |
| MKCRA002-08 | Tracheophyta | Magnoliopsida | Saxifragales | Crassulaceae | Echeverioideae | Echeveria | *Echeveria carminea* | 0 | 93.62 |
| LEDCL013-17 | Tracheophyta | Magnoliopsida | Saxifragales | Crassulaceae | Echeverioideae | Echeveria | *Echeveria crenulata* | 0 | 93.62 |
| LEDCL015-17 | Tracheophyta | Magnoliopsida | Saxifragales | Crassulaceae | Echeverioideae | Echeveria | *Echeveria derenbergii* | 0 | 93.62 |
| LEDCL018-17 | Tracheophyta | Magnoliopsida | Saxifragales | Crassulaceae | Echeverioideae | Echeveria | *Echeveria globuliflora* | 0 | 93.62 |
| LEDCL022-17 | Tracheophyta | Magnoliopsida | Saxifragales | Crassulaceae | Echeverioideae | Echeveria | *Echeveria grisea* | 0 | 93.62 |
| LEDCL024-17 | Tracheophyta | Magnoliopsida | Saxifragales | Crassulaceae | Echeverioideae | Echeveria | *Echeveria halbingeri var. halbingeri* | 0 | 93.62 |
| MKCRA033-08 | Tracheophyta | Magnoliopsida | Saxifragales | Crassulaceae | Echeverioideae | Echeveria | *Echeveria laui* | 0 | 93.62 |
| MKCRA025-08 | Tracheophyta | Magnoliopsida | Saxifragales | Crassulaceae | Echeverioideae | Echeveria | *Echeveria longissima var. nov.* | 0 | 93.62 |
| MKCRA045-08 | Tracheophyta | Magnoliopsida | Saxifragales | Crassulaceae | Echeverioideae | Echeveria | *Echeveria microcalyx* | 0 | 93.62 |
| LEDCL042-17 | Tracheophyta | Magnoliopsida | Saxifragales | Crassulaceae | Echeverioideae | Echeveria | *Echeveria paniculata var. maculata* | 0 | 93.62 |
| MKCRA022-08 | Tracheophyta | Magnoliopsida | Saxifragales | Crassulaceae | Echeverioideae | Echeveria | *Echeveria platyphylla* | 0 | 93.62 |
| LEDCL048-17 | Tracheophyta | Magnoliopsida | Saxifragales | Crassulaceae | Echeverioideae | Echeveria | *Echeveria rodolfi* | 0 | 93.62 |
| LEDCL053-17 | Tracheophyta | Magnoliopsida | Saxifragales | Crassulaceae | Echeverioideae | Echeveria | *Echeveria semivestita var. floresiana* | 0 | 93.62 |
| LEDCL054-17 | Tracheophyta | Magnoliopsida | Saxifragales | Crassulaceae | Echeverioideae | Echeveria | *Echeveria sessiliflora* | 0 | 93.62 |
| MKCRA013-08 | Tracheophyta | Magnoliopsida | Saxifragales | Crassulaceae | Echeverioideae | Echeveria | *Echeveria setosa* | 0 | 93.62 |
| LEDCL056-17 | Tracheophyta | Magnoliopsida | Saxifragales | Crassulaceae | Echeverioideae | Echeveria | *Echeveria setosa var. deminuta* | 0 | 93.62 |
| LEDCL058-17 | Tracheophyta | Magnoliopsida | Saxifragales | Crassulaceae | Echeverioideae | Echeveria | *Echeveria simulans* | 0 | 93.62 |
| LEDCL091-17 | Tracheophyta | Magnoliopsida | Saxifragales | Crassulaceae | Echeverioideae | Echeveria | *Echeveria uhlii* | 0 | 93.62 |
| MKCRA094-08 | Tracheophyta | Magnoliopsida | Saxifragales | Crassulaceae | Echeverioideae | Thompsonella | *Thompsonella xochipalensis* | 0 | 93.62 |
| MKCRA029-08 | Tracheophyta | Magnoliopsida | Saxifragales | Crassulaceae | Echeverioideae | Echeveria | *Echeveria minima* | 0 | 93.61 |
| MKCRA077-08 | Tracheophyta | Magnoliopsida | Saxifragales | Crassulaceae | Echeverioideae | Cremnophila | *Cremnophila nutans* | 0 | 93.49 |
| MKCRA005-08 | Tracheophyta | Magnoliopsida | Saxifragales | Crassulaceae | Echeverioideae | Echeveria | *Echeveria chapalensis* | 0 | 93.49 |
| MKCRA023-08 | Tracheophyta | Magnoliopsida | Saxifragales | Crassulaceae | Echeverioideae | Echeveria | *Echeveria longissima var. longissima* | 0 | 93.49 |
| MKCRA041-08 | Tracheophyta | Magnoliopsida | Saxifragales | Crassulaceae | Echeverioideae | Echeveria | *Echeveria affinis* | 0 | 93.37 |
| MKCRA037-08 | Tracheophyta | Magnoliopsida | Saxifragales | Crassulaceae | Echeverioideae | Echeveria | *Echeveria lutea* | 0 | 93.37 |
| MKCRA031-08 | Tracheophyta | Magnoliopsida | Saxifragales | Crassulaceae | Echeverioideae | Echeveria | *Echeveria sp.* | 0 | 93.37 |
| MKCRA035-08 | Tracheophyta | Magnoliopsida | Saxifragales | Crassulaceae | Echeverioideae | Echeveria | *Echeveria subsessilis* | 0 | 93.37 |
| MKCRA081-08 | Tracheophyta | Magnoliopsida | Saxifragales | Crassulaceae | Sedoideae | Villadia | *Villadia albiflora* | 0 | 93.37 |
| MKCRA001-08 | Tracheophyta | Magnoliopsida | Saxifragales | Crassulaceae | Echeverioideae | Echeveria | *Echeveria coccinea* | 0 | 93.24 |
| NNOR1917-16 | Tracheophyta | Magnoliopsida | Saxifragales | Crassulaceae | Sempervivoideae | Sedum | *Sedum acre* | 2 | 92.13 |
| PNOR1665-18 | Tracheophyta | Magnoliopsida | Saxifragales | Crassulaceae | Sempervivoideae | Sedum | *Sedum acre* | 2 | 92.13 |
| PNOR1666-18 | Tracheophyta | Magnoliopsida | Saxifragales | Crassulaceae | Sempervivoideae | Sedum | *Sedum anglicum* | 0 | 91.93 |
| FPUK173-14 | Tracheophyta | Magnoliopsida | Saxifragales | Crassulaceae | Sempervivoideae | Sedum | *Sedum anglicum* | 0 | 91.45 |
| POWNA1779-12 | Tracheophyta | Magnoliopsida | Saxifragales | Crassulaceae | Sempervivoideae | Sedum | *Sedum anglicum* | 0 | 91.43 |
| PNOR1668-18 | Tracheophyta | Magnoliopsida | Saxifragales | Crassulaceae | Sempervivoideae | Sedum | *Sedum forsterianum* | 8 | 91.27 |
| GBVJ1629-11 | Tracheophyta | Magnoliopsida | Saxifragales | Crassulaceae |  | Hylotelephium | *Hylotelephium spectabile* | 8 | 90.73 |
| PNOR886-17 | Tracheophyta | Magnoliopsida | Saxifragales | Crassulaceae |  | Rhodiola | *Rhodiola quadrifida* | 10 | 90.66 |
