# Supplementary material for: Characterization and evolutionary insights into complete mitochondrial genome of Sedum sarmentosum within the family Crassulaceae
Source: Front Plant Sci. 2026 Feb 6;17:1710625. doi: 10.3389/fpls.2026.1710625 (PMC12920544; doi:10.3389/fpls.2026.1710625)
Supplement: Supplementary file 3 [file Table3.docx]

**Table S3 | Relative synonymous codon usage (RSCU) of the mitochondrial genome of *Sedum sarmentosum.***

| **Amino** | **Codon 1 RSCU** | **Codon 2 RSCU** | **Codon 3 RSCU** | **Codon 4 RSCU** | **Codon 5 RSCU** | **Codon 6 RSCU** |
| --- | --- | --- | --- | --- | --- | --- |
| Ala | GCG(0.52) | GCC(0.83) | GCA(1.03) | GCU(1.62) |  |  |
| Arg | CGC(0.57) | AGG(0.72) | CGG(0.57) | CGU(1.28) | CGA(1.29) | AGA(1.21) |
| Asn | AAC(0.62) | AAU(1.38) |  |  |  |  |
| Asp | GAC(0.59) | GAU(1.41) |  |  |  |  |
| Cys | UGC(0.89) | UGU(1.11) |  |  |  |  |
| Gln | CAG(0.47) | CAA(1.53) |  |  |  |  |
| Glu | GAG(0.59) | GAA(1.41) |  |  |  |  |
| Gly | GGC(0.57) | GGG(0.79) | GGU(1.21) | GGA(1.39) |  |  |
| His | CAC(0.5) | CAU(1.50) |  |  |  |  |
| Ile | AUC(0.86) | AUA(0.8) | AUU(1.35) |  |  |  |
| Leu | CUG(0.57) | CUC(0.62) | CUA(0.83) | UUG(1.20) | CUU(1.27) | UUA(1.51) |
| Lys | AAG(0.71) | AAA(1.29) |  |  |  |  |
| Met | AUG(1.00) |  |  |  |  |  |
| Phe | UUC(0.82) | UUU(1.18) |  |  |  |  |
| Pro | CCG(0.6) | CCC(0.91) | CCA(1.21) | CCU(1.28) |  |  |
| Ser | AGC(0.63) | UCG(0.83) | UCC(0.97) | AGU(1.00) | UCA(1.27) | UCU(1.29) |
| Thr | ACG(0.64) | ACC(1.04) | ACA(1.00) | ACU(1.32) |  |  |
| Trp | UGG(1.00) |  |  |  |  |  |
| Tyr | UAC(0.51) | UAU(1.49) |  |  |  |  |
| Val | GUC(0.76) | GUG(0.85) | GUA(1.15) | GUU(1.24) |  |  |
| End | UAG(1.19) | UGA(0.65) | UAA(1.16) |  |  |  |
