# Supplementary material for: Characterization and evolutionary insights into complete mitochondrial genome of Sedum sarmentosum within the family Crassulaceae
Source: Front Plant Sci. 2026 Feb 6;17:1710625. doi: 10.3389/fpls.2026.1710625 (PMC12920544; doi:10.3389/fpls.2026.1710625)
Supplement: Supplementary file 4 [file Table4.docx]

**Table S4 | MISA in the mitochondrial genome of *Sedum sarmentosum.***

| **Chr** | **SSR type** | **SSR** | **size** | **start** | **end** |
| --- | --- | --- | --- | --- | --- |
| mtChr1 | p3 | (TTC)3 | 9 | 7091 | 7099 |
| mtChr1 | c | (TAT)3ataggcatctaatagaataaatgaaactctttttatttcacattcaataattc(TAT)3atattaa(TAT)3 | 87 | 7649 | 7735 |
| mtChr1 | p3 | (CAA)3 | 9 | 8246 | 8254 |
| mtChr1 | p3 | (TTA)3 | 9 | 9499 | 9507 |
| mtChr1 | p3 | (TAT)3 | 9 | 13162 | 13170 |
| mtChr1 | p3 | (TCT)3 | 9 | 23166 | 23174 |
| mtChr1 | p3 | (AGG)3 | 9 | 24036 | 24044 |
| mtChr1 | p3 | (ACT)3 | 9 | 27254 | 27262 |
| mtChr1 | p3 | (ATC)3 | 9 | 30423 | 30431 |
| mtChr1 | p4 | (CTGC)3 | 12 | 31297 | 31308 |
| mtChr1 | p3 | (TGT)3 | 9 | 32990 | 32998 |
| mtChr1 | p3 | (GGA)3 | 9 | 38635 | 38643 |
| mtChr1 | p4 | (GCCG)3 | 12 | 39072 | 39083 |
| mtChr1 | p3 | (CTG)3 | 9 | 39698 | 39706 |
| mtChr1 | p4 | (TTTC)3 | 12 | 42212 | 42223 |
| mtChr1 | p3 | (CAG)3 | 9 | 42334 | 42342 |
| mtChr1 | p3 | (TCT)3 | 9 | 44073 | 44081 |
| mtChr1 | p3 | (TTG)3 | 9 | 44539 | 44547 |
| mtChr1 | p3 | (TTC)3 | 9 | 45910 | 45918 |
| mtChr1 | p3 | (CGT)3 | 9 | 48708 | 48716 |
| mtChr1 | p3 | (TTC)3 | 9 | 50720 | 50728 |
| mtChr1 | p3 | (TCC)3 | 9 | 51546 | 51554 |
| mtChr1 | p4 | (AAGT)3 | 12 | 54833 | 54844 |
| mtChr1 | p3 | (CCT)3 | 9 | 56769 | 56777 |
| mtChr1 | p3 | (TAA)5 | 15 | 58871 | 58885 |
| mtChr1 | p3 | (AAG)3 | 9 | 59586 | 59594 |
| mtChr1 | p3 | (GAG)3 | 9 | 60077 | 60085 |
| mtChr1 | p4 | (AAAG)3 | 12 | 61562 | 61573 |
| mtChr1 | p4 | (ATAA)3 | 12 | 62804 | 62815 |
| mtChr1 | p3 | (TAG)3 | 9 | 63339 | 63347 |
| mtChr1 | p3 | (ACG)3 | 9 | 68007 | 68015 |
| mtChr1 | p3 | (TAA)3 | 9 | 68713 | 68721 |
| mtChr1 | p3 | (GAT)3 | 9 | 68955 | 68963 |
| mtChr1 | c | (ATA)3agtcaagtcaag(AAT)3 | 30 | 70637 | 70666 |
| mtChr1 | p3 | (GAA)3 | 9 | 74581 | 74589 |
| mtChr1 | p4 | (ACTC)3 | 12 | 75357 | 75368 |
| mtChr1 | p3 | (TTA)3 | 9 | 78290 | 78298 |
| mtChr1 | p3 | (CAA)3 | 9 | 80927 | 80935 |
| mtChr1 | p3 | (TCC)3 | 9 | 84787 | 84795 |
| mtChr1 | p3 | (TCT)3 | 9 | 87231 | 87239 |
| mtChr1 | p3 | (CCT)3 | 9 | 87869 | 87877 |
| mtChr1 | p4 | (CATT)3 | 12 | 91292 | 91303 |
| mtChr1 | p3 | (AGA)3 | 9 | 91431 | 91439 |
| mtChr1 | p3 | (TGG)3 | 9 | 93661 | 93669 |
| mtChr1 | p3 | (ACT)3 | 9 | 96498 | 96506 |
| mtChr1 | p3 | (TTG)3 | 9 | 100787 | 100795 |
| mtChr1 | p3 | (CTA)3 | 9 | 104508 | 104516 |
| mtChr1 | p3 | (TTA)4 | 12 | 104783 | 104794 |
| mtChr1 | p3 | (TCT)3 | 9 | 110288 | 110296 |
| mtChr1 | p3 | (GTA)3 | 9 | 110952 | 110960 |
| mtChr1 | p3 | (TTG)3 | 9 | 111209 | 111217 |
| mtChr1 | p3 | (AGT)3 | 9 | 111404 | 111412 |
| mtChr1 | p3 | (TAT)3 | 9 | 112090 | 112098 |
| mtChr1 | p3 | (AAG)3 | 9 | 112405 | 112413 |
| mtChr1 | p3 | (TCC)3 | 9 | 116986 | 116994 |
| mtChr1 | p3 | (ATT)3 | 9 | 117381 | 117389 |
| mtChr1 | p3 | (GAA)3 | 9 | 120411 | 120419 |
| mtChr1 | p3 | (CAC)3 | 9 | 120850 | 120858 |
| mtChr1 | c | (CTT)3atttac(TGT)3 | 24 | 127877 | 127900 |
| mtChr1 | p3 | (TTC)3 | 9 | 128065 | 128073 |
| mtChr1 | p3 | (TTC)3 | 9 | 128535 | 128543 |
| mtChr1 | c | (CTT)3tag(GGAC)3 | 24 | 128807 | 128830 |
| mtChr1 | p3 | (GCT)3 | 9 | 130843 | 130851 |
| mtChr1 | p3 | (GTT)3 | 9 | 133277 | 133285 |
| mtChr1 | p3 | (GAA)3 | 9 | 137019 | 137027 |
| mtChr1 | p3 | (GCC)3 | 9 | 139160 | 139168 |
| mtChr1 | p3 | (CTA)3 | 9 | 140195 | 140203 |
| mtChr1 | p3 | (CTG)3 | 9 | 140404 | 140412 |
| mtChr1 | p3 | (ACG)3 | 9 | 140964 | 140972 |
| mtChr1 | c | (TCT)3ttcacggggagaagcagcaacgattcatattttttgtctcattttt(TTA)3 | 64 | 142246 | 142309 |
| mtChr1 | p4 | (CTTC)3 | 12 | 143210 | 143221 |
| mtChr1 | p3 | (GAA)3 | 9 | 143910 | 143918 |
| mtChr1 | p3 | (CAA)3 | 9 | 144635 | 144643 |
| mtChr1 | p3 | (TTC)3 | 9 | 145385 | 145393 |
| mtChr1 | p3 | (TTG)3 | 9 | 147397 | 147405 |
| mtChr1 | p3 | (ATT)3 | 9 | 147668 | 147676 |
| mtChr1 | p3 | (CAC)3 | 9 | 149524 | 149532 |
| mtChr1 | p3 | (CTA)3 | 9 | 150037 | 150045 |
| mtChr1 | p3 | (TAT)3 | 9 | 152005 | 152013 |
| mtChr1 | p3 | (ATT)3 | 9 | 153286 | 153294 |
| mtChr1 | p3 | (TGA)3 | 9 | 153872 | 153880 |
| mtChr1 | p3 | (GCT)3 | 9 | 154003 | 154011 |
| mtChr1 | p3 | (GTT)3 | 9 | 156453 | 156461 |
