# Supplementary material for: Characterization and evolutionary insights into complete mitochondrial genome of Sedum sarmentosum within the family Crassulaceae
Source: Front Plant Sci. 2026 Feb 6;17:1710625. doi: 10.3389/fpls.2026.1710625 (PMC12920544; doi:10.3389/fpls.2026.1710625)
Supplement: Supplementary file 5 [file Table5.docx]

**Table S5 | Dispersed repeats in the mitochondrial genome of *Sedum sarmentosum*.**

| **The Chr that the first part located** | **The repeat length of the first part** | **The start site of the first part** | **Type** | **The Chr that the second part located** | **The repeat length of the second part** | **The start site of the second part** | **Interval distance of repeats** | **E-value** |
| --- | --- | --- | --- | --- | --- | --- | --- | --- |
| mtChr1 | 116306 | 116389 | P | mtChr1 | 132125 | 132208 | 0 | 7.39E-41 |
| mtChr1 | 40714 | 40764 | P | mtChr1 | 114633 | 114683 | 0 | 5.45E-21 |
| mtChr1 | 16118 | 16154 | F | mtChr1 | 32501 | 32537 | 0 | 1.46E-12 |
| mtChr1 | 40681 | 40713 | P | mtChr1 | 78378 | 78410 | 0 | 3.75E-10 |
| mtChr1 | 16940 | 16971 | F | mtChr1 | 55626 | 55657 | 0 | 1.50E-09 |
| mtChr1 | 91044 | 91075 | P | mtChr1 | 123018 | 123049 | 0 | 1.50E-09 |
| mtChr1 | 147826 | 147854 | F | mtChr1 | 155294 | 155322 | 0 | 9.59E-08 |
| mtChr1 | 78369 | 78395 | F | mtChr1 | 114675 | 114701 | 0 | 1.53E-06 |
| mtChr1 | 112636 | 112662 | P | mtChr1 | 135163 | 135189 | 0 | 1.53E-06 |
| mtChr1 | 140142 | 140168 | P | mtChr1 | 140142 | 140168 | 0 | 1.53E-06 |
| mtChr1 | 10971 | 10996 | F | mtChr1 | 12410 | 12435 | 0 | 6.14E-06 |
| mtChr1 | 22044 | 22068 | P | mtChr1 | 128544 | 128568 | 0 | 2.45E-05 |
| mtChr1 | 109166 | 109190 | P | mtChr1 | 128544 | 128568 | 0 | 2.45E-05 |
| mtChr1 | 3699 | 3722 | P | mtChr1 | 63627 | 63650 | 0 | 9.82E-05 |
| mtChr1 | 14745 | 14768 | P | mtChr1 | 55434 | 55457 | 0 | 9.82E-05 |
| mtChr1 | 22035 | 22058 | F | mtChr1 | 78680 | 78703 | 0 | 9.82E-05 |
| mtChr1 | 78680 | 78703 | F | mtChr1 | 109157 | 109180 | 0 | 9.82E-05 |
| mtChr1 | 30755 | 30777 | P | mtChr1 | 117625 | 117647 | 0 | 3.93E-04 |
| mtChr1 | 84895 | 84916 | P | mtChr1 | 147028 | 147049 | 0 | 1.57E-03 |
| mtChr1 | 9815 | 9835 | F | mtChr1 | 78640 | 78660 | 0 | 6.28E-03 |
| mtChr1 | 111678 | 111698 | F | mtChr1 | 145979 | 145999 | 0 | 6.28E-03 |
| mtChr1 | 132795 | 132815 | P | mtChr1 | 132795 | 132815 | 0 | 6.28E-03 |
| mtChr1 | 11953 | 11972 | F | mtChr1 | 58997 | 59016 | 0 | 2.51E-02 |
| mtChr1 | 17578 | 17597 | P | mtChr1 | 124739 | 124758 | 0 | 2.51E-02 |
| mtChr1 | 21051 | 21070 | F | mtChr1 | 72627 | 72646 | 0 | 2.51E-02 |
| mtChr1 | 26845 | 26864 | P | mtChr1 | 50084 | 50103 | 0 | 2.51E-02 |
| mtChr1 | 48150 | 48169 | P | mtChr1 | 66149 | 66168 | 0 | 2.51E-02 |
| mtChr1 | 72627 | 72646 | F | mtChr1 | 108173 | 108192 | 0 | 2.51E-02 |
| mtChr1 | 111629 | 111648 | R | mtChr1 | 111629 | 111648 | 0 | 2.51E-02 |
| mtChr1 | 151817 | 151836 | R | mtChr1 | 151817 | 151836 | 0 | 2.51E-02 |
