# Supplementary material for: Characterization and evolutionary insights into complete mitochondrial genome of Sedum sarmentosum within the family Crassulaceae
Source: Front Plant Sci. 2026 Feb 6;17:1710625. doi: 10.3389/fpls.2026.1710625 (PMC12920544; doi:10.3389/fpls.2026.1710625)
Supplement: Supplementary file 6 [file Table6.docx]

**Table S6 | Mitochondrial-Plastid DNA Transfer of *Sedum sarmentosum.***

| **query** | **database** | **identity (%)** | **alignment** | **mismatch** | **gap** | **q. start** | **q. end** | **d. start** | **d. end** | **e-value** | **score** |
| --- | --- | --- | --- | --- | --- | --- | --- | --- | --- | --- | --- |
| ptDNA | mtDNA | 96.231 | 1247 | 21 | 6 | 53664 | 54910 | 129658 | 130878 | 0 | 2019 |
| ptDNA | mtDNA | 93.155 | 862 | 42 | 8 | 34537 | 35386 | 8659 | 7803 | 0 | 1249 |
| ptDNA | mtDNA | 88.047 | 686 | 49 | 16 | 66666 | 67339 | 145021 | 145685 | 0 | 782 |
| ptDNA | mtDNA | 91.262 | 412 | 32 | 1 | 31903 | 32314 | 75186 | 74779 | 1.42E-158 | 558 |
| ptDNA | mtDNA | 73.596 | 890 | 178 | 39 | 98150 | 99013 | 49793 | 48935 | 2.06E-77 | 289 |
| ptDNA | mtDNA | 73.596 | 890 | 178 | 39 | 132811 | 133674 | 48935 | 49793 | 2.06E-77 | 289 |
| ptDNA | mtDNA | 77.892 | 389 | 51 | 20 | 64062 | 64431 | 63231 | 62859 | 1.65E-53 | 209 |
| ptDNA | mtDNA | 95.946 | 74 | 3 | 0 | 1 | 74 | 151100 | 151173 | 7.95E-27 | 121 |
| ptDNA | mtDNA | 93.151 | 73 | 5 | 0 | 50675 | 50747 | 8767 | 8695 | 6.19E-23 | 108 |
