# Supplementary material for: Characterization and evolutionary insights into complete mitochondrial genome of Sedum sarmentosum within the family Crassulaceae
Source: Front Plant Sci. 2026 Feb 6;17:1710625. doi: 10.3389/fpls.2026.1710625 (PMC12920544; doi:10.3389/fpls.2026.1710625)
Supplement: Supplementary file 7 [file Table7.docx]

**Table S7 | RNA editing sites prediction in the mitochondrial genome of *Sedum sarmentosum.***

| **Gene** | **Base** | **Aa** | **Triplet pos.** | **Codon** | **Aa change** | **Left motifs** | **Right motifs** | **Probability** |
| --- | --- | --- | --- | --- | --- | --- | --- | --- |
| *nad6* | 26 | 9 | 2 | CCA->CUA | P->L | ACTTTCTGTTTTGTCCAGCC | AGCTTTGGTCTCTGGTTTGA | 1 |
| *nad6* | 83 | 28 | 2 | UCG->UUG | S->L | AAATCCAGTACATTCCGTTT | GTTTTTTATCCTCGTTTTTC | 0.997 |
| *nad6* | 103 | 35 | 1 | CGC->UGC | R->C | CGTTTTTTATCCTCGTTTTT | GCGACACTTCAGGTTTACTT | 0.952 |
| *nad6* | 161 | 54 | 2 | CCA->CUA | P->L | CTTCTTCGCTATGATCTTTC | AGTAGTTCATATAGGAGCTA | 1 |
| *nad6* | 169 | 57 | 1 | CAU->UAU | H->Y | CTATGATCTTTCCAGTAGTT | ATATAGGAGCTATAGCTGTT | 0.995 |
| *nad6* | 191 | 64 | 2 | UCA->UUA | S->L | TATAGGAGCTATAGCTGTTT | ATTCCTATTCGTTGTTATGA | 0.994 |
| *nad6* | 569 | 190 | 2 | UCU->UUU | S->F | TCGACGAAATGCTATGGATT | TAGGAGGACCATAATGAGGA | 0.998 |
| *rpl5* | 35 | 12 | 2 | UCA->UUA | S->L | TTTTCATTACGAAGATGTAT | ACGTCAGGATCCGTTGCTCA | 0.99 |
| *rpl5* | 47 | 16 | 2 | CCG->CUG | P->L | AGATGTATCACGTCAGGATC | GTTGCTCAAACCGAATCACG | 0.998 |
| *rpl5* | 59 | 20 | 2 | CCG->CUG | P->L | TCAGGATCCGTTGCTCAAAC | GAATCACGCCAACGTTATGG | 0.998 |
| *rpl5* | 64 | 22 | 1 | CAC->UAC | H->Y | ATCCGTTGCTCAAACCGAAT | ACGCCAACGTTATGGAAGTT | 0.94 |
| *rpl5* | 92 | 31 | 2 | UCG->UUG | S->L | CGTTATGGAAGTTCCTGGAT | GTGTGAAATAAGAGTAGTAC | 0.999 |
| *rpl5* | 317 | 106 | 2 | UCG->UUG | S->L | GCATGGAATGTCTAATTTTT | GGTCAGAATCTCGACAGTAA | 0.999 |
| *rpl5* | 329 | 110 | 2 | UCG->UUG | S->L | TAATTTTTCGGTCAGAATCT | GACAGTAATGTCTCTGTTAG | 0.963 |
| *rpl5* | 417 | 139 | 3 | UCC->UCU | S->S | ACGGAGTTTTGCGAATTCTC | CCAGAACTTGAAGATCATTT | 0.914 |
| *rpl5* | 512 | 171 | 2 | CCA->CUA | P->L | CACACAAGGTGAGACTTTAC | ACCGTGGAGCGGCTTTTTGC | 0.987 |
| *rpl5* | 515 | 172 | 2 | CCG->CUG | P->L | ACAAGGTGAGACTTTACCAC | GTGGAGCGGCTTTTTGCAAA | 0.98 |
| *nad7* | 44 | 15 | 2 | UCC->UUC | S->F | GAAAAATTTCACATTGAATT | CGGACCTCAACATCCTGCTG | 0.988 |
| *nad7* | 45 | 15 | 3 | UCC->UCU | S->S | AAAAATTTCACATTGAATTC | GGACCTCAACATCCTGCTGC | 0.99 |
| *nad7* | 77 | 26 | 2 | UCA->UUA | S->L | TCCTGCTGCTCATGGTGTTT | ACGATTAGTATTGGAAATGA | 0.999 |
| *nad7* | 137 | 46 | 2 | UCA->UUA | S->L | TGCGGAACCACATATTGGAT | ACTCCAGTGCGGCACGAAGC | 0.997 |
| *nad7* | 924 | 308 | 3 | GUC->GUU | V->V | AGCTTCCGAGGTCGAGCAGT | TAAATTTCACTGGAGGATTT | 0.942 |
| *nad7* | 1384 | 462 | 1 | CCC->UCC | P->S | GAGGCTAGGGCTGCCTCCTT | CCACCGCGTCCTTCCTTGTG | 0.903 |
| *nad7* | 2175 | 725 | 3 | CUC->CUU | L->L | CACCCAATCTTCCTTTTGCT | TTCCTTCGGTTTGCCTCCAT | 0.911 |
| *nad7* | 2601 | 867 | 3 | CUC->CUU | L->L | CGATGGCCCAAGAACACGCT | ATTCTTCAGCCGTAGAGATA | 0.999 |
| *nad7* | 2608 | 870 | 1 | CAG->UAG | Q->* | CCAAGAACACGCTCATTCTT | AGCCGTAGAGATACTTTTGA | 0.993 |
| *nad7* | 2673 | 891 | 3 | UCC->UCU | S->S | AATATATACGAGTGTTATTC | GTGAAATAACTCGAATTTCA | 0.997 |
| *nad7* | 2692 | 898 | 1 | CAA->UAA | Q->* | CCGTGAAATAACTCGAATTT | AAATCATTCACTTGCTTTAA | 0.999 |
| *nad7* | 2701 | 901 | 1 | CAC->UAC | H->Y | AACTCGAATTTCAAATCATT | ACTTGCTTTAACTACTCATG | 0.999 |
| *nad7* | 2740 | 914 | 1 | CAA->UAA | Q->* | TGCTATGGATGTGGGAGCAT | AACTCCGTTCCTGTGGGCTT | 1 |
| *nad7* | 2890 | 964 | 1 | CCA->UCA | P->S | ATGTCGAGATATTGATTCCT | CACACAACAATTTGCTTCTC | 0.998 |
| *nad7* | 3036 | 1012 | 3 | GUC->GUU | V->V | GTGGTGTAATGTTAAGAGGT | GTGCGACATGAAGACATTGA | 0.972 |
| *nad7* | 3425 | 1142 | 2 | UCU->UUU | S->F | ATGGAACATGGTAAGCCTAT | TTTCTCCATATGGAAGTGCT | 0.933 |
| *nad7* | 4058 | 1353 | 2 | UCA->UUA | S->L | GAGCAGCACCTTACGATGTT | ATGACCAATCGGATCCTGAC | 1 |
| *nad7* | 4068 | 1356 | 3 | AUC->AUU | I->I | TTACGATGTTCATGACCAAT | GGATCCTGACGTACCAGTAG | 1 |
| *nad7* | 4073 | 1358 | 2 | UCC->UUC | S->F | ATGTTCATGACCAATCGGAT | CTGACGTACCAGTAGGTACC | 0.999 |
| *nad7* | 4074 | 1358 | 3 | UCC->UCU | S->S | TGTTCATGACCAATCGGATC | TGACGTACCAGTAGGTACCA | 0.999 |
| *nad7* | 4103 | 1368 | 2 | UCG->UUG | S->L | CAGTAGGTACCAGAGGAGAT | GCTATGATCGTTACTGTATC | 1 |
| *nad7* | 4123 | 1375 | 1 | CCG->UCG | P->S | CGCTATGATCGTTACTGTAT | CGTATCGAAGAGATGCGACA | 0.922 |
| *nad7* | 4170 | 1390 | 3 | UCC->UCU | S->S | TCGGATCATTGTGCAATGTC | TAATCAAATGCCTAGTGGCA | 0.999 |
| *nad7* | 4413 | 1471 | 3 | UUC->UUU | F->F | ACCCCAATTTTTTATTTCTT | GGAGCCGTTTCTTTTCCCGT | 0.978 |
| *nad7* | 5483 | 1828 | 2 | CCA->CUA | P->L | GGAAGCGGGAGTAATAAAAC | AATAATCTCTTTCTTGTCCT | 0.974 |
| *nad7* | 5896 | 1966 | 1 | CUC->UUC | L->F | GGATCATCGGTCTACTCTAC | TCAATTCACCATTTCGAACC | 0.933 |
| *nad7* | 5916 | 1972 | 3 | ACC->ACU | T->T | CTCAATTCACCATTTCGAAC | TTATACAGAAGGTTTTTCCG | 0.999 |
| *nad7* | 5945 | 1982 | 2 | UCC->UUC | S->F | AAGGTTTTTCCGTACCAGCT | CTTCTACCTATACCGCAGTT | 0.999 |
| *nad7* | 6022 | 2008 | 1 | CUA->UUA | L->L | AGTAATGGAAGCAATCGTCC | TACCGTCGTAAAATAAGAGC | 0.966 |
| *nad7* | 6029 | 2010 | 2 | UCG->UUG | S->L | GAAGCAATCGTCCCTACCGT | GTAAAATAAGAGCACCTGGC | 0.998 |
| *nad7* | 6051 | 2017 | 3 | CUC->CUU | L->L | TAAAATAAGAGCACCTGGCT | TGCCCATTCACAAGGGCTCG | 0.999 |
| *nad7* | 6060 | 2020 | 3 | UUC->UUU | F->F | AGCACCTGGCTCTGCCCATT | ACAAGGGCTCGATTCTATGT | 0.98 |
| *nad7* | 6075 | 2025 | 3 | UUC->UUU | F->F | CCATTCACAAGGGCTCGATT | TATGTCCAAACATCACATGC | 0.992 |
| *nad7* | 6096 | 2032 | 3 | GCC->GCU | A->A | TATGTCCAAACATCACATGC | AGCAGATGTGGTCACCATCA | 1 |
| *nad7* | 6109 | 2037 | 1 | CAC->UAC | H->Y | CACATGCCAGCAGATGTGGT | ACCATCATAGGTACTCAAGA | 0.941 |
| *nad7* | 6138 | 2046 | 3 | GUC->GUU | V->V | AGGTACTCAAGATATTGTGT | TGGAGAGGTGGATAGATAGN | 1 |
| *nad9* | 92 | 31 | 2 | CCU->CUU | P->L | ATCGGAACATGGGAATAGAC | TGAGACTAATACGGACTACC | 0.937 |
| *nad9* | 113 | 38 | 2 | CCA->CUA | P->L | TGAGACTAATACGGACTACC | ATTTCCATTGTTGTGCTTTC | 0.999 |
| *nad9* | 167 | 56 | 2 | UCG->UUG | S->L | CTATACAAGGGTTCAAGTTT | GATCGATATTTGCGGAGTTG | 0.998 |
| *nad9* | 190 | 64 | 1 | CAU->UAU | H->Y | TCGATATTTGCGGAGTTGAT | ATCCCTCTCGAAAACGAAGA | 1 |
| *nad9* | 298 | 100 | 1 | CCG->UCG | P->S | ACGAAGTAACACGAATATCC | CGGTAGTAAGCCCATTTCCA | 0.984 |
| *nad9* | 311 | 104 | 2 | CCA->CUA | P->L | AATATCCCCGGTAGTAAGCC | ATTTCCATCAGCCGGCCGGT | 0.998 |
| *nad9* | 328 | 110 | 1 | CGG->UGG | R->W | GCCCATTTCCATCAGCCGGC | GGTGGGAGCGAGAAGTTTGG | 1 |
| *nad9* | 368 | 123 | 2 | UCC->UUC | S->F | GGATATGTTTGGTGTTTCTT | CATCAATCATCCGGATCTAC | 1 |
| *nad9* | 398 | 133 | 2 | UCA->UUA | S->L | TCCGGATCTACGCCGTATAT | AACAGATTATGGTTTCGAGG | 0.998 |
| *nad9* | 439 | 147 | 1 | CUU->UUU | L->F | GTCATCCATTACGAAAAGAC | TTCCTCTGAGTGGATATGTG | 0.998 |
| *nad9* | 539 | 180 | 2 | UCU->UUU | S->F | AGAATTTCGCTATTTCGATT | TGCTAGTCCTTGGGAACAGC | 1 |
| *nad2* | 24 | 8 | 3 | ACC->ACU | T->T | TTGAAAGGTTTGTAGTTTAC | CAGTAGTTTTCGGAGTTTGG | 0.985 |
| *nad2* | 350 | 117 | 2 | CCC->CUC | P->L | GTTAGGATTGGGAGGCTTCC | CTCCCATTTAAGTATCAGTA | 0.952 |
| *nad2* | 366 | 122 | 3 | AUC->AUU | I->I | TTCCCCTCCCATTTAAGTAT | AGTAAAGACTATGCCAACTC | 0.996 |
| *nad2* | 555 | 185 | 3 | AUC->AUU | I->I | GTTTCATTCTTGAAATGTAT | GGTTTTTTTGAAAAAATAAA | 0.913 |
| *nad2* | 771 | 257 | 3 | GUC->GUU | V->V | TGCTTTCAACGACCTTAAGT | GGGGCCTAATTCCTCGTCTT | 0.931 |
| *nad2* | 1229 | 410 | 2 | CCG->CUG | P->L | TTAAGGAAAGGAAGAATTCC | GGGTGCCTTAACTCGATTTG | 0.991 |
| *nad2* | 1623 | 541 | 3 | ACC->ACU | T->T | TCTATTGATTCGAACCGAAC | GCATAACCATTGCTTTGGAA | 0.957 |
| *nad2* | 2768 | 923 | 2 | UCC->UUC | S->F | ATGCAGAAGTCAATTATTTT | CAATTGGAAATATGTTCTAA | 0.958 |
| *nad2* | 3093 | 1031 | 3 | CCC->CCU | P->P | GCCTCGGCATCTGTCTTGCC | GTCTGAATGATGTCCCAATG | 0.903 |
| *nad2* | 3462 | 1154 | 3 | CUC->CUU | L->L | CGTAGGTTCGAATCCTGCCT | TCCCACTTGTTTGTTGTTGA | 0.93 |
| *nad2* | 3735 | 1245 | 3 | UUC->UUU | F->F | GACAGACTGCCTCCTTGGTT | GGAACGAAGCCAATACATGC | 0.984 |
| *nad2* | 4116 | 1372 | 3 | UUC->UUU | F->F | AGATCACTGCGGTTCCTTTT | GGGCGGCTGTAGGACGGACG | 0.962 |
| *nad2* | 4686 | 1562 | 3 | CCC->CCU | P->P | CTTTAGTACCGTACCCTACC | CCGACTGCCTTCGCGCCAAG | 0.908 |
| *nad2* | 5130 | 1710 | 3 | UUC->UUU | F->F | GAAAGAATGCCCGCCAAGTT | AGATAAGGTAAGAATGTTTC | 0.931 |
| *nad2* | 5532 | 1844 | 3 | AUC->AUU | I->I | TACAAAAGAAAAGAGAGAAT | CACTTCAGATAACCACGCCT | 0.904 |
| *nad2* | 5907 | 1969 | 3 | UUC->UUU | F->F | TGCGCCTAAAATTTCTATTT | TGCTAATATTTCACGTGTTT | 0.999 |
| *nad2* | 5919 | 1973 | 3 | UUC->UUU | F->F | TTCTATTTCTGCTAATATTT | ACGTGTTTCTATTTATGGTT | 0.981 |
| *nad2* | 5928 | 1976 | 3 | UUC->UUU | F->F | TGCTAATATTTCACGTGTTT | TATTTATGGTTCCTATGGAG | 0.998 |
| *nad2* | 6039 | 2013 | 3 | ACC->ACU | T->T | CCAAACGAAAGTAAAAAGAC | TCTAGCTCATAGTTCAATTG | 0.997 |
| *nad2* | 6047 | 2016 | 2 | UCA->UUA | S->L | AAGTAAAAAGACCTCTAGCT | ATAGTTCAATTGGACATGTA | 0.993 |
| *nad2* | 6077 | 2026 | 2 | UCG->UUG | S->L | TTGGACATGTAGGTTATATT | GTACTGGTTTATCATGTGGA | 0.999 |
| *nad2* | 6081 | 2027 | 3 | UAC->UAU | Y->Y | ACATGTAGGTTATATTCGTA | TGGTTTATCATGTGGAACCA | 0.991 |
| *nad2* | 6147 | 2049 | 3 | AUC->AUU | I->I | TGGTATCTTTATTTATGCAT | AATGACGATAAATGCATTCG | 0.996 |
| *nad2* | 6177 | 2059 | 3 | UUC->UUU | F->F | AAATGCATTCGCCATAGTTT | AGCATTACGGCAAACCCGTG | 0.996 |
| *nad2* | 6246 | 2082 | 3 | UUC->UUU | F->F | AGCCAAAACGAATCCTATTT | GGCTATTACCTTCTCAATTA | 0.992 |
| *nad2* | 6365 | 2122 | 2 | CCC->CUC | P->L | GTGGGGCTTACTTCCTAGCC | CAGTGGGAGTAGTGACTAGC | 0.995 |
| *nad2* | 7594 | 2532 | 1 | CGU->UGU | R->C | CCTAACCCAACCCCTTACTT | GTAGAGCCGTGTATTGTAAG | 0.924 |
| *nad2* | 7738 | 2580 | 1 | CGG->UGG | R->W | AGTTACTACCCCCGTGGTTT | GGGGTGGACCCTTTCACTCT | 0.964 |
| *nad2* | 7749 | 2583 | 3 | CCC->CCU | P->P | CCGTGGTTTCGGGGTGGACC | TTTCACTCTATTTTATTATA | 0.984 |
| *nad2* | 7779 | 2593 | 3 | AGC->AGU | S->S | ATTTTATTATATACGCTTAG | GAAAAGAATGTTTTTTGATA | 1 |
| *nad2* | 7881 | 2627 | 3 | UUC->UUU | F->F | GACTTCCTCTTTCATTACTT | ATCCTTTCCATATCCCTCTC | 0.99 |
| *nad2* | 7884 | 2628 | 3 | AUC->AUU | I->I | TTCCTCTTTCATTACTTCAT | CTTTCCATATCCCTCTCCCT | 0.99 |
| *nad2* | 7889 | 2630 | 2 | UCC->UUC | S->F | CTTTCATTACTTCATCCTTT | CATATCCCTCTCCCTTGTTC | 0.993 |
| *nad2* | 7890 | 2630 | 3 | UCC->UCU | S->S | TTTCATTACTTCATCCTTTC | ATATCCCTCTCCCTTGTTCT | 0.99 |
| *nad2* | 7897 | 2633 | 1 | CUC->UUC | L->F | ACTTCATCCTTTCCATATCC | TCTCCCTTGTTCTCAGTTAC | 0.955 |
| *nad2* | 7938 | 2646 | 3 | UUC->UUU | F->F | TCATCAAATGGCACTCAGTT | ATATCTTTAAGTTCGATCAT | 1 |
| *nad2* | 8139 | 2713 | 3 | AUC->AUU | I->I | GACTCTTTCTGGAACTATAT | ATAAACAGATCTTCCCCTCC | 0.989 |
| *nad2* | 8154 | 2718 | 3 | UCC->UCU | S->S | TATATCATAAACAGATCTTC | CCTCCACACCAATCACGAGT | 0.995 |
| *nad2* | 8182 | 2728 | 1 | CAU->UAU | H->Y | ACCAATCACGAGTTTTTCTC | ATTCCTCTCGTATATCGTCG | 0.992 |
| *nad2* | 8191 | 2731 | 1 | CGU->UGU | R->C | GAGTTTTTCTCCATTCCTCT | GTATATCGTCGTAACACCCT | 0.942 |
| *nad2* | 8198 | 2733 | 2 | UCG->UUG | S->L | TCTCCATTCCTCTCGTATAT | GTCGTAACACCCTTAATGCT | 0.979 |
| *nad2* | 8239 | 2747 | 1 | CAU->UAU | H->Y | AGGTTTTGAAAAAGACTTTT | ATGTCATTCCCATTTAGGCC | 0.988 |
| *nad2* | 8248 | 2750 | 1 | CCC->UCC | P->S | AAAAGACTTTTCATGTCATT | CCATTTAGGCCCGATTCGGA | 0.987 |
| *nad2* | 8259 | 2753 | 3 | GCC->GCU | A->A | CATGTCATTCCCATTTAGGC | CGATTCGGATCCCCCCGTTG | 0.94 |
| *nad2* | 8260 | 2754 | 1 | CGA->UGA | R->* | ATGTCATTCCCATTTAGGCC | GATTCGGATCCCCCCGTTGT | 0.984 |
| *nad2* | 8265 | 2755 | 3 | UUC->UUU | F->F | ATTCCCATTTAGGCCCGATT | GGATCCCCCCGTTGTTTCCT | 0.957 |
| *nad2* | 8271 | 2757 | 3 | UCC->UCU | S->S | ATTTAGGCCCGATTCGGATC | CCCCGTTGTTTCCTTTTCCT | 0.982 |
| *nad2* | 8275 | 2759 | 1 | CGU->UGU | R->C | AGGCCCGATTCGGATCCCCC | GTTGTTTCCTTTTCCTCCCG | 0.977 |
| *nad2* | 8283 | 2761 | 3 | UUC->UUU | F->F | TTCGGATCCCCCCGTTGTTT | CTTTTCCTCCCGCACCTTTT | 0.992 |
| *nad2* | 8290 | 2764 | 1 | CUC->UUC | L->F | CCCCCCGTTGTTTCCTTTTC | TCCCGCACCTTTTCCTCGAA | 0.966 |
| *nad2* | 8292 | 2764 | 3 | CUC->CUU | L->L | CCCCGTTGTTTCCTTTTCCT | CCGCACCTTTTCCTCGAAAT | 0.998 |
| *nad2* | 8304 | 2768 | 3 | UUC->UUU | F->F | CTTTTCCTCCCGCACCTTTT | CTCGAAATGATAAAGAAGAT | 0.91 |
| *nad2* | 8305 | 2769 | 1 | CUC->UUC | L->F | TTTTCCTCCCGCACCTTTTC | TCGAAATGATAAAGAAGATG | 0.987 |
| *nad2* | 8415 | 2805 | 3 | GUC->GUU | V->V | ACCGGATTATTCAAATAAGT | GTGTTTTCCGTGGTTTTCCC | 0.998 |
| *nad2* | 8424 | 2808 | 3 | UCC->UCU | S->S | TTCAAATAAGTCGTGTTTTC | GTGGTTTTCCCATCTTACAA | 0.997 |
| *nad2* | 8491 | 2831 | 1 | CAU->UAU | H->Y | AATGAATCGGTTAACCATTC | ATTAGGGAGCCCGGTATTGA | 0.965 |
| *nad2* | 8503 | 2835 | 1 | CGG->UGG | R->W | AACCATTCCATTAGGGAGCC | GGTATTGACTCTTCTGTGTG | 0.999 |
| *nad2* | 8535 | 2845 | 3 | CUC->CUU | L->L | TTCTGTGTGGTATTCACTCT | GTTCGGCTCTTGGAATCACA | 0.933 |
| *nad2* | 8539 | 2847 | 1 | CGG->UGG | R->W | GTGTGGTATTCACTCTCGTT | GGCTCTTGGAATCACATCCA | 0.991 |
| *nad2* | 8578 | 2860 | 1 | CGC->UGC | R->C | CAGCAGTGGTTGGAACAGCT | GCAAAATCCAAGCACTTCAC | 0.997 |
| *nad2* | 8586 | 2862 | 3 | AUC->AUU | I->I | GTTGGAACAGCTCGCAAAAT | CAAGCACTTCACCTACTTCA | 0.978 |
| *nad2* | 8587 | 2863 | 1 | CAA->UAA | Q->* | TTGGAACAGCTCGCAAAATC | AAGCACTTCACCTACTTCAT | 0.978 |
| *nad2* | 8596 | 2866 | 1 | CAC->UAC | H->Y | CTCGCAAAATCCAAGCACTT | ACCTACTTCATTGCCCCCAA | 0.99 |
| *nad2* | 8605 | 2869 | 1 | CAU->UAU | H->Y | TCCAAGCACTTCACCTACTT | ATTGCCCCCAACCGTTTCTC | 0.979 |
| *nad2* | 8613 | 2871 | 3 | CCC->CCU | P->P | CTTCACCTACTTCATTGCCC | CAACCGTTTCTCGTACCTCT | 0.926 |
| *nad2* | 8614 | 2872 | 1 | CAA->UAA | Q->* | TTCACCTACTTCATTGCCCC | AACCGTTTCTCGTACCTCTA | 0.992 |
| *nad2* | 8623 | 2875 | 1 | CUC->UUC | L->F | TTCATTGCCCCCAACCGTTT | TCGTACCTCTATTGAAACTG | 0.992 |
| *nad2* | 8625 | 2875 | 3 | CUC->CUU | L->L | CATTGCCCCCAACCGTTTCT | GTACCTCTATTGAAACTGAA | 0.976 |
| *nad2* | 8659 | 2887 | 1 | CUU->UUU | L->F | AACTGAATGGTTTCATGTTC | TTCATCGATTGGTTATTCTT | 0.999 |
| *nad2* | 8662 | 2888 | 1 | CAU->UAU | H->Y | TGAATGGTTTCATGTTCCTT | ATCGATTGGTTATTCTTCTC | 0.985 |
| *nad2* | 8665 | 2889 | 1 | CGA->UGA | R->* | ATGGTTTCATGTTCCTTCAT | GATTGGTTATTCTTCTCTGT | 0.963 |
| *nad2* | 8677 | 2893 | 1 | CUU->UUU | L->F | TCCTTCATCGATTGGTTATT | TTCTCTGTTCGTATCTCTTT | 0.997 |
| *nad2* | 8680 | 2894 | 1 | CUC->UUC | L->F | TTCATCGATTGGTTATTCTT | TCTGTTCGTATCTCTTTTTC | 0.952 |
| *nad2* | 8707 | 2903 | 1 | CGG->UGG | R->W | CGTATCTCTTTTTCCAATTT | GGTCTCGATTAGTTCACAAG | 0.995 |
| *nad2* | 9056 | 3019 | 2 | UCC->UUC | S->F | AACACTTAAATTTTACCTTT | CTAACTATTTGCAAATGCAG | 0.964 |
| *nad2* | 9079 | 3027 | 1 | CGG->UGG | R->W | AACTATTTGCAAATGCAGCT | GGCTAGCCTATCCCTAAGGC | 0.911 |
| *atp8* | 47 | 16 | 2 | UCA->UUA | S->L | TTTTACACAATTCTTCTGGT | ATGCCTCTTCTTCTTTACTT | 0.998 |
| *atp8* | 54 | 18 | 3 | CUC->CUU | L->L | CAATTCTTCTGGTCATGCCT | TTCTTCTTTACTTTCTATAT | 0.982 |
| *atp8* | 76 | 26 | 1 | CCC->UCC | P->S | TCTTCTTTACTTTCTATATT | CCATATGCAATGATGGAGAT | 0.997 |
| *atp8* | 452 | 151 | 2 | CCA->CUA | P->L | AATAATGCTAATCCATGTTC | ACACGGCCAAGGAAGCATCG | 1 |
| *rps14* | 198 | 66 | 3 | CUC->CUU | L->L | TGTATTTCCACAGGCCCCCT | TCTATAAGAAATGTATGANN | 0.943 |
| *nad3* | 5 | 2 | 2 | UCA->UUA | S->L | NNNNNNNNNNNNNNNNATGT | AGAATTTGCACCTATTTGTA | 0.966 |
| *nad3* | 44 | 15 | 2 | CCG->CUG | P->L | TATCTATTTAGTGATCAGTC | GCTAGTTTCTTTGATCCCAC | 1 |
| *nad3* | 61 | 21 | 1 | CCA->UCA | P->S | GTCCGCTAGTTTCTTTGATC | CACTCGGTGTTCCTTTTCCA | 0.948 |
| *nad3* | 62 | 21 | 2 | CCA->CUA | P->L | TCCGCTAGTTTCTTTGATCC | ACTCGGTGTTCCTTTTCCAT | 0.999 |
| *nad3* | 80 | 27 | 2 | CCA->CUA | P->L | CCCACTCGGTGTTCCTTTTC | ATTTGCTTCCAATAGTTCGA | 1 |
| *nad3* | 124 | 42 | 1 | CAC->UAC | H->Y | ATCCAGAAAAATTGTCGGCC | ACGAATGTGGTTCCGATCCT | 0.999 |
| *nad3* | 137 | 46 | 2 | UCC->UUC | S->F | GTCGGCCCACGAATGTGGTT | CGATCCTTCCGGTGATGCCA | 1 |
| *nad3* | 146 | 49 | 2 | UCC->UUC | S->F | CGAATGTGGTTCCGATCCTT | CGGTGATGCCAGAAGTCGTT | 1 |
| *nad3* | 208 | 70 | 1 | CCU->UCU | P->S | TTTCCATTTTATTTATTATT | CTGATCCGGAAGTAACCTTT | 0.998 |
| *nad3* | 209 | 70 | 2 | CCU->CUU | P->L | TTCCATTTTATTTATTATTC | TGATCCGGAAGTAACCTTTT | 0.999 |
| *nad3* | 215 | 72 | 2 | CCG->CUG | P->L | TTTATTTATTATTCCTGATC | GGAAGTAACCTTTTCCTTTC | 0.992 |
| *nad3* | 230 | 77 | 2 | UCC->UUC | S->F | TGATCCGGAAGTAACCTTTT | CTTTCCTTGGGCAGTACCTC | 0.999 |
| *nad3* | 247 | 83 | 1 | CCU->UCU | P->S | TTTCCTTTCCTTGGGCAGTA | CTCCCAACAAGATTGATCCG | 0.992 |
| *nad3* | 251 | 84 | 2 | CCC->CUC | P->L | CTTTCCTTGGGCAGTACCTC | CAACAAGATTGATCCGTTTG | 0.998 |
| *nad3* | 266 | 89 | 2 | CCG->CUG | P->L | ACCTCCCAACAAGATTGATC | GTTTGGATCTTGGTCCATGA | 1 |
| *nad3* | 275 | 92 | 2 | UCU->UUU | S->F | CAAGATTGATCCGTTTGGAT | TTGGTCCATGATGGCCTTTT | 0.998 |
| *nad3* | 344 | 115 | 2 | UCG->UUG | S->L | TGAATGGAAAAGGGGTGCTT | GGATCGGGAGTAANNNNNNN | 0.999 |
| *nad3* | 349 | 117 | 1 | CGG->UGG | R->W | GGAAAAGGGGTGCTTCGGAT | GGGAGTAANNNNNNNNNNNN | 0.999 |
| *rps13* | 26 | 9 | 2 | UCA->UUA | S->L | ATATATTTCAAGAGCAGGCT | AGTTGCCGATGAACAAGTAA | 0.928 |
| *rps13* | 56 | 19 | 2 | UCA->UUA | S->L | TGAACAAGTAAGAATTGCCT | AACAAAAATGGATGGAATTG | 0.992 |
| *rps13* | 100 | 34 | 1 | CGU->UGU | R->C | CTAAAAAAGCCATTCAGGTT | GTTATCGATTAGGTATCAGT | 0.993 |
| *rps13* | 287 | 96 | 2 | UCG->UUG | S->L | AATTCGTCATCAAGATGGAT | GCCCTTACGCGGTCAACGAA | 0.999 |
| *atp6* | 145 | 49 | 1 | CCA->UCA | P->S | TGTATTTCTCATTCACAAAT | CATCTTTGTTTATGCTGCTA | 1 |
| *atp6* | 224 | 75 | 2 | UCA->UUA | S->L | TAAAAAGGGAGGAGGAAACT | AGTACCAAATGCTTGGCAAT | 1 |
| *atp6* | 275 | 92 | 2 | CCG->CUG | P->L | GCTGATTTATGATTTCGTGC | GAACCTGGTAAACGAACAAA | 1 |
| *atp6* | 337 | 113 | 1 | CGC->UGC | R->C | TTAAACAAACGTTTTTCCCT | GCATCTCGGTAACTTTTACT | 0.956 |
| *atp6* | 344 | 115 | 2 | UCG->UUG | S->L | AACGTTTTTCCCTCGCATCT | GGTAACTTTTACTTTTTTGT | 0.929 |
| *atp6* | 370 | 124 | 1 | CGU->UGU | R->C | CTTTTACTTTTTTGTTATTT | GTAATCCCCAGGGTATGATA | 0.995 |
| *atp6* | 377 | 126 | 2 | CCC->CUC | P->L | TTTTTTGTTATTTCGTAATC | CCAGGGTATGATACCTTATA | 0.998 |
| *atp6* | 378 | 126 | 3 | CCC->CCU | P->P | TTTTTGTTATTTCGTAATCC | CAGGGTATGATACCTTATAG | 0.903 |
| *atp6* | 509 | 170 | 2 | UCA->UUA | S->L | GCTTTATTTTTTAAGCTTCT | ATTACCCGCTGGAGTCCCAC | 0.99 |
| *atp6* | 568 | 190 | 1 | CCU->UCU | P->S | TAGTACTACTTGAGCTAATC | CTCATTGTTTTCGCGCATTA | 0.999 |
| *atp6* | 571 | 191 | 1 | CAU->UAU | H->Y | TACTACTTGAGCTAATCCCT | ATTGTTTTCGCGCATTAAGC | 0.999 |
| *atp6* | 593 | 198 | 2 | UCA->UUA | S->L | TTGTTTTCGCGCATTAAGCT | AGGAATACGTTTATTCGCTA | 0.997 |
| *atp6* | 635 | 212 | 2 | UCA->UUA | S->L | TATGATGGCCGGTCATAGTT | AGTAAAGATTTTAAGTGGGT | 1 |
| *atp6* | 707 | 236 | 2 | CCU->CUU | P->L | TTTTTATTTCATAGGAGATC | TGGTCCTTTATTTATAGTTC | 0.997 |
| *atp6* | 743 | 248 | 2 | CCA->CUA | P->L | AGTTCTTGCATTAACCGGTC | AGAATTAGGTGTAGCTATAT | 0.998 |
| *atp6* | 764 | 255 | 2 | UCA->UUA | S->L | AGAATTAGGTGTAGCTATAT | ACAAGCTCATGTTTCTACGA | 0.997 |
| *atp6* | 772 | 258 | 1 | CAU->UAU | H->Y | GTGTAGCTATATCACAAGCT | ATGTTTCTACGATCTCAATC | 0.99 |
| *atp6* | 779 | 260 | 2 | UCU->UUU | S->F | TATATCACAAGCTCATGTTT | TACGATCTCAATCTGTATTT | 0.997 |
| *atp6* | 788 | 263 | 2 | UCA->UUA | S->L | AGCTCATGTTTCTACGATCT | AATCTGTATTTACTTGAATG | 1 |
| *atp6* | 815 | 272 | 2 | ACA->AUA | T->I | TATTTACTTGAATGATGCTA | AAATCTCCATCAAAGTAGTT | 1 |
| *atp6* | 826 | 276 | 1 | CAA->UAA | Q->* | ATGATGCTACAAATCTCCAT | AAAGTAGTTCTTTTTTTTTA | 0.976 |
| *nad4* | 30 | 10 | 3 | UUC->UUU | F->F | CATTTCTGTGAATGCTATTT | GATCTAAGTGGTCTTATTCC | 0.929 |
| *nad4* | 50 | 17 | 2 | CCG->CUG | P->L | CGATCTAAGTGGTCTTATTC | GTGTCCCGTGCTAGGAAGCA | 1 |
| *nad4* | 74 | 25 | 2 | ACU->AUU | T->I | TCCCGTGCTAGGAAGCATTA | TCCTCTTTTCATTCCCAATT | 0.991 |
| *nad4* | 77 | 26 | 2 | CCU->CUU | P->L | CGTGCTAGGAAGCATTACTC | TCTTTTCATTCCCAATTCAA | 0.99 |
| *nad4* | 107 | 36 | 2 | CCG->CUG | P->L | TCCCAATTCAAGAATACGAC | GATACGATTGATTGGTCTGT | 0.994 |
| *nad4* | 154 | 52 | 1 | CCC->UCC | P->S | CTCTTATTACTTTTTTGTAT | CCCCTGTTCCTCGGATACAA | 0.999 |
| *nad4* | 158 | 53 | 2 | CCU->CUU | P->L | TATTACTTTTTTGTATCCCC | TGTTCCTCGGATACAATTTG | 0.997 |
| *nad4* | 164 | 55 | 2 | CCU->CUU | P->L | TTTTTTGTATCCCCCTGTTC | TCGGATACAATTTGATCCTT | 0.992 |
| *nad4* | 166 | 56 | 1 | CGG->UGG | R->W | TTTTGTATCCCCCTGTTCCT | GGATACAATTTGATCCTTCT | 1 |
| *nad4* | 197 | 66 | 2 | UCU->UUU | S->F | TGATCCTTCTACGGCCAAAT | TCAATTTGTGGAAAGCCTTC | 0.998 |
| *nad4* | 271 | 91 | 1 | CUU->UUU | L->F | GGGGTATAGACGGTATCTCT | TTTTCTTCGTGATATTGACC | 0.979 |
| *nad4* | 317 | 106 | 2 | UCA->UUA | S->L | TCTGATCCCTATTTGCATTT | AGTGGGTTGGTCTGGTATGA | 0.999 |
| *nad4* | 362 | 121 | 2 | GCA->GUA | A->V | TTATGGGAAAGAGTATATTG | AGCATCTCTAATTCGTGAAT | 0.97 |
| *nad4* | 368 | 123 | 2 | UCU->UUU | S->F | GAAAGAGTATATTGCAGCAT | TCTAATTCGTGAATTTTTAA | 0.997 |
| *nad4* | 403 | 135 | 1 | CGC->UGC | R->C | TTTTAATGATCGCCGTGTTC | GCATGCTGGATCCTCTACTA | 0.999 |
| *nad4* | 416 | 139 | 2 | CCU->CUU | P->L | CGTGTTCCGCATGCTGGATC | TCTACTATTCTATGTTCTTC | 0.992 |
| *nad4* | 433 | 145 | 1 | CUU->UUU | L->F | ATCCTCTACTATTCTATGTT | TTCCCGAAAGCGTGTCAATC | 0.993 |
| *nad4* | 436 | 146 | 1 | CCC->UCC | P->S | CTCTACTATTCTATGTTCTT | CCGAAAGCGTGTCAATCCCT | 0.999 |
| *nad4* | 437 | 146 | 2 | CCC->CUC | P->L | TCTACTATTCTATGTTCTTC | CGAAAGCGTGTCAATCCCTA | 0.992 |
| *nad4* | 709 | 237 | 1 | CGU->UGU | R->C | ACTCTGAGTAGTGGGTACCT | GTAGGACCTCGACCCGCCTA | 0.925 |
| *nad4* | 997 | 333 | 1 | CUC->UUC | L->F | CGCAGCAGGCGGGTTGGTTC | TCTGTCGTCGCCGGGGGGCT | 0.927 |
| *nad4* | 1983 | 661 | 3 | AUC->AUU | I->I | AACCACCGATTTACAAATAT | ATTAACCACAGAATTTAGTG | 0.999 |
| *nad4* | 2034 | 678 | 3 | UUC->UUU | F->F | AATCTTTCTATGGATTGCTT | TTTCGCCTCTTTCGCCGTCA | 0.999 |
| *nad4* | 2142 | 714 | 3 | UCC->UCU | S->S | CGTCATCTTGGCAGGAATTC | TTTAAAATTGGGAACCTACG | 0.999 |
| *nad4* | 2211 | 737 | 3 | UUC->UUU | F->F | TCCCGAAGCGACACTTTGTT | CACTCCTTTCATTTATACTC | 0.995 |
| *nad4* | 2232 | 744 | 3 | UCC->UCU | S->S | CACTCCTTTCATTTATACTC | AAGCGCGATTGCAATAATAT | 0.973 |
| *nad4* | 2262 | 754 | 3 | CUC->CUU | L->L | TGCAATAATATATACTTCCT | GACCACTTCAAGACAGATCG | 0.997 |
| *nad4* | 2271 | 757 | 3 | UUC->UUU | F->F | ATATACTTCCTCGACCACTT | AAGACAGATCGATCTTAAGA | 0.998 |
| *nad4* | 3076 | 1026 | 1 | CAC->UAC | H->Y | TTCTCTCCCCGGCATCGTCT | ACACAGAAAGAGTGCGGAGC | 0.911 |
| *nad4* | 4295 | 1432 | 2 | UCA->UUA | S->L | TCGGAAAGCGCCTTATAAAT | ACCCCATTCATCCAAAAGAG | 0.984 |
| *nad4* | 4567 | 1523 | 1 | CGG->UGG | R->W | AAGAAAAATCCCAGTGGGGC | GGTGCCTTTCTTTTACGGCC | 0.949 |
| *nad4* | 5102 | 1701 | 2 | UCU->UUU | S->F | AGGGAATTGGAGGTAGCATT | TACCGATGTCAAGTCATGGA | 0.989 |
| *nad4* | 5106 | 1702 | 3 | ACC->ACU | T->T | AATTGGAGGTAGCATTCTAC | GATGTCAAGTCATGGACTGG | 1 |
| *nad4* | 5112 | 1704 | 3 | GUC->GUU | V->V | AGGTAGCATTCTACCGATGT | AAGTCATGGACTGGTTCCTT | 1 |
| *nad4* | 5129 | 1710 | 2 | UCC->UUC | S->F | TGTCAAGTCATGGACTGGTT | CTTCAGCCCTTTTTCTATGT | 0.999 |
| *nad4* | 5197 | 1733 | 1 | CGG->UGG | R->W | ACTCGACTTGTTAGGTATTA | GGAGGTTTAGTGAGCACCAT | 0.99 |
| *nad4* | 5225 | 1742 | 2 | UCU->UUU | S->F | TAGTGAGCACCATGCCGAAT | TCTCTACCATTTCCTTCTCT | 0.999 |
| *nad4* | 5238 | 1746 | 3 | UUC->UUU | F->F | GCCGAATCTCTCTACCATTT | CTTCTCTTCCACTTTGGCCA | 0.989 |
| *nad4* | 5244 | 1748 | 3 | CUC->CUU | L->L | TCTCTCTACCATTTCCTTCT | TTCCACTTTGGCCAATATGA | 0.998 |
| *nad4* | 5247 | 1749 | 3 | UUC->UUU | F->F | CTCTACCATTTCCTTCTCTT | CACTTTGGCCAATATGAGTT | 0.995 |
| *nad4* | 5248 | 1750 | 1 | CAC->UAC | H->Y | TCTACCATTTCCTTCTCTTC | ACTTTGGCCAATATGAGTTC | 0.923 |
| *nad4* | 5268 | 1756 | 3 | UUC->UUU | F->F | CACTTTGGCCAATATGAGTT | ACCTGGTACTAGCAGCTTTA | 1 |
| *nad4* | 5307 | 1769 | 3 | CUC->CUU | L->L | TATCGGGGAATTTCTCATCT | AGTAGGAGCTTTCCAAAGAA | 0.999 |
| *nad4* | 5403 | 1801 | 3 | UGC->UGU | C->C | CCTTTGGCTATATAATCGTG | GGTTTCTGGAAATTTAAAAC | 0.999 |
| *nad4* | 5451 | 1817 | 3 | UCC->UCU | S->S | CCTCCATAAATTCTCTGATC | AAATGGCAGAGAAGTTTCCA | 0.975 |
| *nad4* | 5469 | 1823 | 3 | UUC->UUU | F->F | TCCAAATGGCAGAGAAGTTT | CATATTTTTACCTTTTATTG | 0.997 |
| *nad4* | 5984 | 1995 | 2 | CCG->CUG | P->L | ATGGAAACCAGAAGCACGAC | GGGGTCTTCGTAGTCCGAGA | 0.904 |
| *nad4* | 6296 | 2099 | 2 | CCG->CUG | P->L | AGTAAAGATAACGAGCGCTC | GCTTTCTTAAAAGCAAGGAC | 0.975 |
| *nad4* | 7134 | 2378 | 3 | UUC->UUU | F->F | AACTATTGACCCTACTTGTT | GGATGGGTGTTCACCCCAAA | 0.999 |
| *nad4* | 7146 | 2382 | 3 | UUC->UUU | F->F | TACTTGTTCGGATGGGTGTT | ACCCCAAAGTGTTCCCGGAC | 0.998 |
| *nad4* | 7162 | 2388 | 1 | CGG->UGG | R->W | TGTTCACCCCAAAGTGTTCC | GGACCGCATGCATACATCCG | 0.999 |
| *nad4* | 7167 | 2389 | 3 | ACC->ACU | T->T | ACCCCAAAGTGTTCCCGGAC | GCATGCATACATCCGTAAGT | 0.991 |
| *nad5* | 271 | 91 | 1 | CGG->UGG | R->W | TGCTTTTTATGAAGTCGCAT | GGGAGCTAGTGCTTGCTATC | 1 |
| *nad5* | 1123 | 375 | 1 | CAC->UAC | H->Y | AATTAGAATGAGGGGTTCAC | ACATCATTGCATGCAAGGGG | 0.911 |
| *nad5* | 1192 | 398 | 1 | CCG->UCG | P->S | CTCGTAATTCACTTCTGACT | CGTGTTCGATAGCCCGACCG | 0.976 |
| *nad5* | 1207 | 403 | 1 | CGA->UGA | R->* | TGACTCCGTGTTCGATAGCC | GACCGTAGTGATGTTAATTG | 0.999 |
| *nad5* | 1237 | 413 | 1 | CCA->UCA | P->S | GATGTTAATTGTGGTTACAT | CATAAGTAGCTTGGTCCATC | 0.999 |
| *nad5* | 1280 | 427 | 2 | CCG->CUG | P->L | TATTCCATTTCATATATGTC | GAGGATCCGCATAGCCCTCG | 0.989 |
| *nad5* | 1323 | 441 | 3 | UUC->UUU | F->F | TTATGTGTTATTTATCCATT | CTACTTTTTTTATGCCAATG | 0.994 |
| *nad5* | 1324 | 442 | 1 | CUA->UUA | L->L | TATGTGTTATTTATCCATTC | TACTTTTTTTATGCCAATGT | 0.999 |
| *nad5* | 1339 | 447 | 1 | CAA->UAA | Q->* | CATTCCTACTTTTTTTATGC | AATGTTGGTGACTGGAGATA | 0.996 |
| *nad5* | 1363 | 455 | 1 | CUC->UUC | L->F | GTTGGTGACTGGAGATAACT | TCTTCAATTATTCCTGGGAT | 1 |
| *nad5* | 1504 | 502 | 1 | CUG->UUG | L->L | AGGTGATTTTGGATTAGCTC | TGGGATTTCGGGTCGTTTTA | 1 |
| *nad5* | 1513 | 505 | 1 | CGG->UGG | R->W | TGGATTAGCTCCTGGGATTT | GGGTCGTTTTACTCTTTTTC | 0.999 |
| *nad5* | 1518 | 506 | 3 | GUC->GUU | V->V | TAGCTCCTGGGATTTCGGGT | GTTTTACTCTTTTTCAAACT | 0.991 |
| *nad5* | 1563 | 521 | 3 | CUC->CUU | L->L | ACTTTTCAACCATTTTTGCT | GTGCTAGTGCCCCCATAAAT | 0.998 |
| *nad5* | 1573 | 525 | 1 | CCC->UCC | P->S | CATTTTTGCTCGTGCTAGTG | CCCCATAAATTCTTGGATTT | 0.98 |
| *nad5* | 1594 | 532 | 1 | CUU->UUU | L->F | CCCCATAAATTCTTGGATTT | TTGCAATATGAGATTGAATG | 0.996 |
| *nad5* | 1641 | 547 | 3 | UUC->UUU | F->F | CTCTTATTTGTATTTTACTT | TTATTGGTGCTGTTGGGAAA | 1 |
| *nad5* | 1678 | 560 | 1 | CGC->UGC | R->C | GAAATCCGCACAGATAGGAT | GCATACTTGGTCACCCGATG | 0.998 |
| *nad5* | 1690 | 564 | 1 | CAC->UAC | H->Y | GATAGGATCGCATACTTGGT | ACCCGATGCTATGGAGGGTC | 1 |
| *nad5* | 1800 | 600 | 3 | ACC->ACU | T->T | GCTCCCCTTTATTTGAATAC | CACCTACGGCTTTGATTGTT | 1 |
| *nad5* | 1828 | 610 | 1 | CUG->UUG | L->L | GGCTTTGATTGTTATTACTT | TGCAGGAGCTACGACGTCAT | 0.999 |
| *nad5* | 1840 | 614 | 1 | CGA->UGA | R->* | TATTACTTCTGCAGGAGCTA | GACGTCATTCCTTGCGGCAA | 1 |
| *nad5* | 2149 | 717 | 1 | CAA->UAA | Q->* | TCTAATTGGATTTCCTTTTC | AACTGGATTTTATTCTAAAG | 0.999 |
| *nad5* | 2275 | 759 | 1 | CAC->UAC | H->Y | TTCTTATTACTCTTTTCGTT | ACTTTTTTTAACATTTCTAG | 0.997 |
| *nad5* | 3036 | 1012 | 3 | CUC->CUU | L->L | TACTTGGAGACTTGGGATCT | TGCAGGAAATGCGAAGGTTG | 0.944 |
| *nad5* | 3105 | 1035 | 3 | UUC->UUU | F->F | GATAATCCACTAGTTTAGTT | TGATCTTAGTAGGCTCGTAA | 0.983 |
| *nad5* | 3426 | 1142 | 3 | AUC->AUU | I->I | CGAGCCATAGATGATAGCAT | GATAGCTATCTGTATGGTTC | 0.99 |
| *nad5* | 3566 | 1189 | 2 | ACG->AUG | T->M | TTAAGCGAACTCTTCTCTTA | GCAATTCCTGACTAGTCGTA | 0.905 |
| *nad5* | 3715 | 1239 | 1 | CCG->UCG | P->S | CTGAATTAGGTCGCCTTTCT | CGGAGTATTCTGATCTTTAC | 0.907 |
| *nad5* | 4274 | 1425 | 2 | CCU->CUU | P->L | CGGAAGTGAGGTTAATGCTC | TGTTGTTTGGTTAATGTTGT | 0.961 |
| *nad5* | 4300 | 1434 | 1 | CGG->UGG | R->W | TTTGGTTAATGTTGTTTGTT | GGTGCCCCATTCCATCTCTT | 0.951 |
| *nad5* | 4732 | 1578 | 1 | CUC->UUC | L->F | TTCGCGAAAAGGCCCCTTCC | TCAATATCATGATTGGGTCG | 0.907 |
| *nad5* | 4954 | 1652 | 1 | CGA->UGA | R->* | ACAACCTCTAGCAGATGGTT | GAAATTGATTCTAAAAGAAC | 1 |
| *nad5* | 5002 | 1668 | 1 | CCC->UCC | P->S | ACCAAGTAGTGCTAATTTCT | CCTTTTTAGAATGGCTCCAG | 1 |
| *nad5* | 5052 | 1684 | 3 | CUC->CUU | L->L | TTATGTTAAGTCTGGTAGCT | GGGCCGTTGTACCTTTTGAT | 0.998 |
| *nad5* | 5094 | 1698 | 3 | AUC->AUU | I->I | ATGGTATGGTATTGTCAGAT | TGAACATAGGACTACTTTAT | 0.998 |
| *nad5* | 5163 | 1721 | 3 | GUC->GUU | V->V | ATGGAATTATTATAGCAGGT | GGTCTAGTAATTAGGGGGCG | 0.971 |
| *nad5* | 5383 | 1795 | 1 | CUC->UUC | L->F | GTCATAAAAAAGAGATGTTT | TCTTCGCGCCTCAGCTCAAG | 0.913 |
| *nad5* | 6354 | 2118 | 3 | UCC->UCU | S->S | TGCGGAATTTTTTTTTCGTC | GGTGGATCTGATCTANNNNN | 0.988 |
| *nad1* | 48 | 16 | 3 | CUC->CUU | L->L | CTGAATTAGGTCGCCTTTCT | CGGAGTATTCTGATCTTTAC | 0.907 |
| *nad1* | 607 | 203 | 1 | CUG->UUG | L->L | CGGAAGTGAGGTTAATGCTC | TGTTGTTTGGTTAATGTTGT | 0.961 |
| *nad1* | 633 | 211 | 3 | UUC->UUU | F->F | TTTGGTTAATGTTGTTTGTT | GGTGCCCCATTCCATCTCTT | 0.951 |
| *nad1* | 1065 | 355 | 3 | CCC->CCU | P->P | TTCGCGAAAAGGCCCCTTCC | TCAATATCATGATTGGGTCG | 0.907 |
| *nad1* | 1287 | 429 | 3 | UUC->UUU | F->F | ACAACCTCTAGCAGATGGTT | GAAATTGATTCTAAAAGAAC | 1 |
| *nad1* | 1335 | 445 | 3 | CUC->CUU | L->L | ACCAAGTAGTGCTAATTTCT | CCTTTTTAGAATGGCTCCAG | 1 |
| *nad1* | 1385 | 462 | 2 | UCG->UUG | S->L | TTATGTTAAGTCTGGTAGCT | GGGCCGTTGTACCTTTTGAT | 0.998 |
| *nad1* | 1427 | 476 | 2 | UCU->UUU | S->F | ATGGTATGGTATTGTCAGAT | TGAACATAGGACTACTTTAT | 0.998 |
| *nad1* | 1496 | 499 | 2 | UCG->UUG | S->L | ATGGAATTATTATAGCAGGT | GGTCTAGTAATTAGGGGGCG | 0.971 |
| *nad1* | 1716 | 572 | 3 | UUC->UUU | F->F | GTCATAAAAAAGAGATGTTT | TCTTCGCGCCTCAGCTCAAG | 0.913 |
| *nad1* | 2687 | 896 | 2 | CCG->CUG | P->L | TGCGGAATTTTTTTTTCGTC | GGTGGATCTGATCTATAGTT | 0.966 |
| *nad1* | 2972 | 991 | 2 | UCG->UUG | S->L | TAGTTTATCCACTATTAAGT | GAGCTACTTCGTTCTTATCC | 0.961 |
| *nad1* | 3511 | 1171 | 1 | CAA->UAA | Q->* | TGTAACTGCAGCTCCAATTT | AACAGGTACTCTAGATGATT | 0.999 |
| *nad1* | 3589 | 1197 | 1 | CUA->UUA | L->L | ACTCTCAATGGCTTACGGAT | TATTTCTTTTATTTCTATCT | 0.921 |
| *nad1* | 3608 | 1203 | 2 | UCU->UUU | S->F | TCTATTTCTTTTATTTCTAT | TTTAAAAGCTGAGGCGTGAA | 0.931 |
| *nad1* | 3878 | 1293 | 2 | UCA->UUA | S->L | ATTAAGAAATTGAGTGGTTT | AAGATGTTTTGCAGCAACAG | 0.915 |
| *nad1* | 4649 | 1550 | 2 | CCC->CUC | P->L | AAAGGCCTTTAACTCATCTC | CCTTCCGGGGCTTCATCTCT | 0.929 |
| *nad1* | 4799 | 1600 | 2 | CCU->CUU | P->L | CGTTTAATGCGATTTCGAGC | TTTTGCCTTTCCCTATCAGT | 0.916 |
| *nad1* | 4923 | 1641 | 3 | UCC->UCU | S->S | AGCCCTAGTAGCTGTTATTC | TCTTCCGAGATATATGAATA | 0.961 |
| *nad1* | 5105 | 1702 | 2 | UCU->UUU | S->F | GGTGGAAGAAAAGAATACCT | TCCTTCGCGCCTTGCCCGGG | 0.949 |
| *nad1* | 5421 | 1807 | 3 | UAC->UAU | Y->Y | CCCTTCCCCTTGGATGATTA | CCCTGGGATCAGTAGCTCAT | 0.913 |
| *nad1* | 5609 | 1870 | 2 | UCU->UUU | S->F | ATTTTCTTTTTTTAGTAACT | TTGAATGATTCCTGCCCTAA | 0.93 |
| *nad1* | 5621 | 1874 | 2 | CCU->CUU | P->L | TAGTAACTCTTGAATGATTC | TGCCCTAATGTTGTCTTCGA | 0.919 |
| *rpl10* | 101 | 34 | 2 | UCG->UUG | S->L | CTCATTCCATTCCAGTGGCT | GACCAGTAACCAATGGCGAA | 0.995 |
| *rpl10* | 134 | 45 | 2 | CCA->CUA | P->L | ATGGCGAAAACTCAAAAATC | ATGGTTTCCCGGTAGAACCC | 0.994 |
| *rpl10* | 155 | 52 | 2 | CCA->CUA | P->L | ATGGTTTCCCGGTAGAACCC | ATTTCGCCCAAGTTGTTGCG | 0.975 |
| *rpl10* | 239 | 80 | 2 | UCG->UUG | S->L | TGCAGGTCCCACTTGTATAT | GTATTTGGCCGAAGAAGCAT | 0.994 |
| *rpl10* | 277 | 93 | 1 | CUA->UUA | L->L | CATCGGACAGGTTTGAGTTC | TACCTTCTTGGGACTCCATG | 0.957 |
| *rpl10* | 314 | 105 | 2 | UCA->UUA | S->L | CATGGACCAAGATCTGCTTT | ATTATATGGGCAATACCGAT | 0.979 |
| *rrn18* | 807 | 269 | 3 | GCC->GCU | A->A | GATACCCTGGTAGTCCATGC | GTAAACGATGAGTGTTCGCC | 0.979 |
| *rrn18* | 1312 | 438 | 1 | CAA->UAA | Q->* | TTTCGCTTGTTTAGTAAAGT | AAGTTTTTGGCCTTATCTTG | 0.959 |
| *rrn18* | 1439 | 480 | 2 | UCC->UUC | S->F | TACGCCCCGCCAAAACGGCT | CGAAACAAAAAGGTGCGTGC | 0.939 |
| *rpl16* | 6 | 2 | 3 | CUC->CUU | L->L | NNNNNNNNNNNNNNNATGCT | CTGCGGAAGTATCTACTCGT | 0.938 |
| *rpl16* | 37 | 13 | 1 | CAG->UAG | Q->* | ATCTACTCGTTACGGAATCT | AGGTGTCAAAGTGTGGATTT | 1 |
| *rpl16* | 183 | 61 | 3 | GGC->GGU | G->G | CTTGGTTTTGGAAGATATGG | ACTAAAAGTTGTAGAGCTGG | 0.935 |
| *rpl16* | 185 | 62 | 2 | ACU->AUU | T->I | TGGTTTTGGAAGATATGGCA | TAAAAGTTGTAGAGCTGGTC | 1 |
| *rpl16* | 313 | 105 | 1 | CUC->UUC | L->F | GGAAGATATGGGTAAGAGTT | TCGCGGATCTCCCTATTACC | 0.984 |
| *rpl16* | 402 | 134 | 3 | UCC->UCU | S->S | GGTTGGATTGCTCGTGTGTC | ACGGGACAAATCCCATTTGA | 0.903 |
| *rpl16* | 416 | 139 | 2 | CCA->CUA | P->L | TGTGTCCACGGGACAAATCC | ATTTGAAATGGATGGTGTGA | 0.999 |
| *ccmC* | 76 | 26 | 1 | CGG->UGG | R->W | GGCAAATTCTCATTGGATCT | GGTTGTTCTTAACAGCGATG | 1 |
| *ccmC* | 103 | 35 | 1 | CAU->UAU | H->Y | TCTTAACAGCGATGGCTATT | ATTTAAGTCTTCGGATAGCA | 0.998 |
| *ccmC* | 115 | 39 | 1 | CGG->UGG | R->W | TGGCTATTCATTTAAGTCTT | GGATAGCACCACTAGATCTT | 0.96 |
| *ccmC* | 133 | 45 | 1 | CUU->UUU | L->F | TTCGGATAGCACCACTAGAT | TTCAACAAGGTGGAAATTCT | 0.98 |
| *ccmC* | 161 | 54 | 2 | CCG->CUG | P->L | AGGTGGAAATTCTCGTATTC | GTATATACATGCTCCTGCGG | 0.998 |
| *ccmC* | 179 | 60 | 2 | GCG->GUG | A->V | TCCGTATATACATGCTCCTG | GGCTCGGATGAGTATTCTTA | 0.995 |
| *ccmC* | 184 | 62 | 1 | CGG->UGG | R->W | ATATACATGCTCCTGCGGCT | GGATGAGTATTCTTATTTAT | 0.963 |
| *ccmC* | 281 | 94 | 2 | ACA->AUA | T->I | TCGCTCTTCCGGAACCGGTA | AGAAATTGGTGCTTTTTCTA | 0.903 |
| *ccmC* | 299 | 100 | 2 | UCU->UUU | S->F | TACAGAAATTGGTGCTTTTT | TACATTGTTTACCTTAGTTA | 0.983 |
| *ccmC* | 331 | 111 | 1 | CGG->UGG | R->W | CCTTAGTTACTGGGGGGTTT | GGGGAAGACCTATGTGGGGC | 0.999 |
| *ccmC* | 358 | 120 | 1 | CGG->UGG | R->W | GACCTATGTGGGGCACCTTT | GGGTGTGGGATGCTCGTTTA | 0.998 |
| *ccmC* | 395 | 132 | 2 | UCG->UUG | S->L | TTTAACCTCTGTATTCATCT | GTTCCTTATTTACCTGGGTG | 0.996 |
| *ccmC* | 399 | 133 | 3 | UUC->UUU | F->F | ACCTCTGTATTCATCTCGTT | CTTATTTACCTGGGTGCACT | 0.994 |
| *ccmC* | 400 | 134 | 1 | CUU->UUU | L->F | CCTCTGTATTCATCTCGTTC | TTATTTACCTGGGTGCACTG | 0.999 |
| *ccmC* | 436 | 146 | 1 | CCU->UCU | P->S | CACTGTGTTTTCAAAAGCAT | CTGTCGAACCGGCTCCTATT | 0.993 |
| *ccmC* | 446 | 149 | 2 | CCG->CUG | P->L | TCAAAAGCATCCTGTCGAAC | GGCTCCTATTTCAATCCGTG | 0.999 |
| *ccmC* | 451 | 151 | 1 | CCU->UCU | P->S | AGCATCCTGTCGAACCGGCT | CTATTTCAATCCGTGCTGGA | 0.989 |
| *ccmC* | 458 | 153 | 2 | UCA->UUA | S->L | TGTCGAACCGGCTCCTATTT | AATCCGTGCTGGACCGATCG | 0.979 |
| *ccmC* | 463 | 155 | 1 | CGU->UGU | R->C | AACCGGCTCCTATTTCAATC | GTGCTGGACCGATCGATATA | 0.999 |
| *ccmC* | 467 | 156 | 2 | GCU->GUU | A->V | GGCTCCTATTTCAATCCGTG | TGGACCGATCGATATACCAA | 0.992 |
| *ccmC* | 473 | 158 | 2 | CCG->CUG | P->L | TATTTCAATCCGTGCTGGAC | GATCGATATACCAATAATAA | 0.998 |
| *ccmC* | 521 | 174 | 2 | UCG->UUG | S->L | AGTCAACTGGTGGAATACAT | GCATCAAGCTGGGAGCATTA | 0.989 |
| *ccmC* | 568 | 190 | 1 | CCU->UCU | P->S | TTGGTACATCAATACATGTT | CTATGCCCATTCCAATCTTG | 0.998 |
| *ccmC* | 575 | 192 | 2 | CCC->CUC | P->L | ATCAATACATGTTCCTATGC | CATTCCAATCTTGTCTAACT | 0.998 |
| *ccmC* | 605 | 202 | 2 | UCC->UUC | S->F | CTTGTCTAACTTTGCTAACT | CCCCTTCTCACTACGTATCT | 0.999 |
| *ccmC* | 608 | 203 | 2 | CCC->CUC | P->L | GTCTAACTTTGCTAACTCCC | CTTCTCACTACGTATCTTAC | 0.949 |
| *ccmC* | 614 | 205 | 2 | UCA->UUA | S->L | CTTTGCTAACTCCCCCTTCT | ACTACGTATCTTACTCGTTC | 0.985 |
| *ccmC* | 624 | 208 | 3 | AUC->AUU | I->I | TCCCCCTTCTCACTACGTAT | TTACTCGTTCTGGAAACACG | 0.975 |
| *ccmC* | 650 | 217 | 2 | CCU->CUU | P->L | CGTTCTGGAAACACGTTTTC | TATTCCATCTTTTCTCGAAT | 0.988 |
| *ccmC* | 656 | 219 | 2 | CCA->CUA | P->L | GGAAACACGTTTTCCTATTC | ATCTTTTCTCGAATCCCCCA | 0.986 |
| *rps12* | 71 | 24 | 2 | UCG->UUG | S->L | CACGGACCGTACTCGAGCTT | GGATCAATGTCCCCAGAAGC | 1 |
| *rps12* | 100 | 34 | 1 | CGC->UGC | R->C | GTCCCCAGAAGCAAGGAGTA | GCCCGCGTGTTTCAACGAGA | 0.999 |
| *rps12* | 104 | 35 | 2 | CCG->CUG | P->L | CCAGAAGCAAGGAGTACGCC | GCGTGTTTCAACGAGAACAC | 1 |
| *rps12* | 146 | 49 | 2 | CCC->CUC | P->L | GAAAAAACCAAATTCAGCTC | CCGTAAGATAGCCAAAGTAC | 0.988 |
| *rps12* | 196 | 66 | 1 | CAC->UAC | H->Y | ATCGACATGATATATTTGCT | ACATTCCGGGCGAAGGTCAT | 0.998 |
| *rps12* | 221 | 74 | 2 | UCG->UUG | S->L | TCCGGGCGAAGGTCATAATT | GCAGGAACATTCCATGGTGT | 0.999 |
| *rps12* | 284 | 95 | 2 | UCC->UUC | S->F | AGATTTGCCAGGTGTGAAAT | CCATTGTATTCGAGGAGTCA | 0.999 |
| *ccmB* | 28 | 10 | 1 | CAU->UAU | H->Y | GACTCTTTCTGGAACTATAT | ATAAACAGATCTTCCCCTCC | 0.989 |
| *ccmB* | 43 | 15 | 1 | CCC->UCC | P->S | TATATCATAAACAGATCTTC | CCTCCACACCAATCACGAGT | 0.995 |
| *ccmB* | 71 | 24 | 2 | CCA->CUA | P->L | ACCAATCACGAGTTTTTCTC | ATTCCTCTCGTATATCGTCG | 0.992 |
| *ccmB* | 80 | 27 | 2 | UCG->UUG | S->L | GAGTTTTTCTCCATTCCTCT | GTATATCGTCGTAACACCCT | 0.942 |
| *ccmB* | 87 | 29 | 3 | AUC->AUU | I->I | TCTCCATTCCTCTCGTATAT | GTCGTAACACCCTTAATGCT | 0.979 |
| *ccmB* | 128 | 43 | 2 | UCA->UUA | S->L | AGGTTTTGAAAAAGACTTTT | ATGTCATTCCCATTTAGGCC | 0.988 |
| *ccmB* | 137 | 46 | 2 | UCC->UUC | S->F | AAAAGACTTTTCATGTCATT | CCATTTAGGCCCGATTCGGA | 0.987 |
| *ccmB* | 148 | 50 | 1 | CCG->UCG | P->S | CATGTCATTCCCATTTAGGC | CGATTCGGATCCCCCCGTTG | 0.94 |
| *ccmB* | 149 | 50 | 2 | CCG->CUG | P->L | ATGTCATTCCCATTTAGGCC | GATTCGGATCCCCCCGTTGT | 0.984 |
| *ccmB* | 154 | 52 | 1 | CGG->UGG | R->W | ATTCCCATTTAGGCCCGATT | GGATCCCCCCGTTGTTTCCT | 0.957 |
| *ccmB* | 160 | 54 | 1 | CCC->UCC | P->S | ATTTAGGCCCGATTCGGATC | CCCCGTTGTTTCCTTTTCCT | 0.982 |
| *ccmB* | 164 | 55 | 2 | CCG->CUG | P->L | AGGCCCGATTCGGATCCCCC | GTTGTTTCCTTTTCCTCCCG | 0.977 |
| *ccmB* | 172 | 58 | 1 | CCU->UCU | P->S | TTCGGATCCCCCCGTTGTTT | CTTTTCCTCCCGCACCTTTT | 0.992 |
| *ccmB* | 179 | 60 | 2 | CCU->CUU | P->L | CCCCCCGTTGTTTCCTTTTC | TCCCGCACCTTTTCCTCGAA | 0.966 |
| *ccmB* | 181 | 61 | 1 | CCC->UCC | P->S | CCCCGTTGTTTCCTTTTCCT | CCGCACCTTTTCCTCGAAAT | 0.998 |
| *ccmB* | 193 | 65 | 1 | CCU->UCU | P->S | CTTTTCCTCCCGCACCTTTT | CTCGAAATGATAAAGAAGAT | 0.91 |
| *ccmB* | 194 | 65 | 2 | CCU->CUU | P->L | TTTTCCTCCCGCACCTTTTC | TCGAAATGATAAAGAAGATG | 0.987 |
| *ccmB* | 304 | 102 | 1 | CGU->UGU | R->C | ACCGGATTATTCAAATAAGT | GTGTTTTCCGTGGTTTTCCC | 0.998 |
| *ccmB* | 313 | 105 | 1 | CGU->UGU | R->C | TTCAAATAAGTCGTGTTTTC | GTGGTTTTCCCATCTTACAA | 0.997 |
| *ccmB* | 380 | 127 | 2 | CCA->CUA | P->L | AATGAATCGGTTAACCATTC | ATTAGGGAGCCCGGTATTGA | 0.965 |
| *ccmB* | 392 | 131 | 2 | CCG->CUG | P->L | AACCATTCCATTAGGGAGCC | GGTATTGACTCTTCTGTGTG | 0.999 |
| *ccmB* | 424 | 142 | 1 | CGU->UGU | R->C | TTCTGTGTGGTATTCACTCT | GTTCGGCTCTTGGAATCACA | 0.933 |
| *ccmB* | 428 | 143 | 2 | UCG->UUG | S->L | GTGTGGTATTCACTCTCGTT | GGCTCTTGGAATCACATCCA | 0.991 |
| *ccmB* | 467 | 156 | 2 | UCG->UUG | S->L | CAGCAGTGGTTGGAACAGCT | GCAAAATCCAAGCACTTCAC | 0.997 |
| *ccmB* | 475 | 159 | 1 | CCA->UCA | P->S | GTTGGAACAGCTCGCAAAAT | CAAGCACTTCACCTACTTCA | 0.978 |
| *ccmB* | 476 | 159 | 2 | CCA->CUA | P->L | TTGGAACAGCTCGCAAAATC | AAGCACTTCACCTACTTCAT | 0.978 |
| *ccmB* | 485 | 162 | 2 | UCA->UUA | S->L | CTCGCAAAATCCAAGCACTT | ACCTACTTCATTGCCCCCAA | 0.99 |
| *ccmB* | 494 | 165 | 2 | UCA->UUA | S->L | TCCAAGCACTTCACCTACTT | ATTGCCCCCAACCGTTTCTC | 0.979 |
| *ccmB* | 502 | 168 | 1 | CCA->UCA | P->S | CTTCACCTACTTCATTGCCC | CAACCGTTTCTCGTACCTCT | 0.926 |
| *ccmB* | 503 | 168 | 2 | CCA->CUA | P->L | TTCACCTACTTCATTGCCCC | AACCGTTTCTCGTACCTCTA | 0.992 |
| *ccmB* | 512 | 171 | 2 | UCU->UUU | S->F | TTCATTGCCCCCAACCGTTT | TCGTACCTCTATTGAAACTG | 0.992 |
| *ccmB* | 514 | 172 | 1 | CGU->UGU | R->C | CATTGCCCCCAACCGTTTCT | GTACCTCTATTGAAACTGAA | 0.976 |
| *ccmB* | 548 | 183 | 2 | CCU->CUU | P->L | AACTGAATGGTTTCATGTTC | TTCATCGATTGGTTATTCTT | 0.999 |
| *ccmB* | 551 | 184 | 2 | UCA->UUA | S->L | TGAATGGTTTCATGTTCCTT | ATCGATTGGTTATTCTTCTC | 0.985 |
| *ccmB* | 554 | 185 | 2 | UCG->UUG | S->L | ATGGTTTCATGTTCCTTCAT | GATTGGTTATTCTTCTCTGT | 0.963 |
| *ccmB* | 566 | 189 | 2 | UCU->UUU | S->F | TCCTTCATCGATTGGTTATT | TTCTCTGTTCGTATCTCTTT | 0.997 |
| *ccmB* | 569 | 190 | 2 | UCU->UUU | S->F | TTCATCGATTGGTTATTCTT | TCTGTTCGTATCTCTTTTTC | 0.952 |
| *ccmB* | 596 | 199 | 2 | UCG->UUG | S->L | CGTATCTCTTTTTCCAATTT | GGTCTCGATTAGTTCACAAG | 0.995 |
| *ccmB* | 649 | 217 | 1 | CAU->UAU | H->Y | GACTCTTTCTGGAACTATAT | ATAAACAGATCTTCCCCTCC | 0.989 |
| *ccmB* | 664 | 222 | 1 | CCC->UCC | P->S | TATATCATAAACAGATCTTC | CCTCCACACCAATCACGAGT | 0.995 |
| *ccmB* | 692 | 231 | 2 | CCA->CUA | P->L | ACCAATCACGAGTTTTTCTC | ATTCCTCTCGTATATCGTCG | 0.992 |
| *ccmB* | 701 | 234 | 2 | UCG->UUG | S->L | GAGTTTTTCTCCATTCCTCT | GTATATCGTCGTAACACCCT | 0.942 |
| *ccmB* | 708 | 236 | 3 | AUC->AUU | I->I | TCTCCATTCCTCTCGTATAT | GTCGTAACACCCTTAATGCT | 0.979 |
| *ccmB* | 749 | 250 | 2 | UCA->UUA | S->L | AGGTTTTGAAAAAGACTTTT | ATGTCATTCCCATTTAGGCC | 0.988 |
| *ccmB* | 758 | 253 | 2 | UCC->UUC | S->F | AAAAGACTTTTCATGTCATT | CCATTTAGGCCCGATTCGGA | 0.987 |
| *ccmB* | 769 | 257 | 1 | CCG->UCG | P->S | CATGTCATTCCCATTTAGGC | CGATTCGGATCCCCCCGTTG | 0.94 |
| *ccmB* | 770 | 257 | 2 | CCG->CUG | P->L | ATGTCATTCCCATTTAGGCC | GATTCGGATCCCCCCGTTGT | 0.984 |
| *ccmB* | 775 | 259 | 1 | CGG->UGG | R->W | ATTCCCATTTAGGCCCGATT | GGATCCCCCCGTTGTTTCCT | 0.957 |
| *ccmB* | 781 | 261 | 1 | CCC->UCC | P->S | ATTTAGGCCCGATTCGGATC | CCCCGTTGTTTCCTTTTCCT | 0.982 |
| *ccmB* | 785 | 262 | 2 | CCG->CUG | P->L | AGGCCCGATTCGGATCCCCC | GTTGTTTCCTTTTCCTCCCG | 0.977 |
| *ccmB* | 793 | 265 | 1 | CCU->UCU | P->S | TTCGGATCCCCCCGTTGTTT | CTTTTCCTCCCGCACCTTTT | 0.992 |
| *ccmB* | 800 | 267 | 2 | CCU->CUU | P->L | CCCCCCGTTGTTTCCTTTTC | TCCCGCACCTTTTCCTCGAA | 0.966 |
| *ccmB* | 802 | 268 | 1 | CCC->UCC | P->S | CCCCGTTGTTTCCTTTTCCT | CCGCACCTTTTCCTCGAAAT | 0.998 |
| *ccmB* | 814 | 272 | 1 | CCU->UCU | P->S | CTTTTCCTCCCGCACCTTTT | CTCGAAATGATAAAGAAGAT | 0.91 |
| *ccmB* | 815 | 272 | 2 | CCU->CUU | P->L | TTTTCCTCCCGCACCTTTTC | TCGAAATGATAAAGAAGATG | 0.987 |
| *ccmB* | 925 | 309 | 1 | CGU->UGU | R->C | ACCGGATTATTCAAATAAGT | GTGTTTTCCGTGGTTTTCCC | 0.998 |
| *ccmB* | 934 | 312 | 1 | CGU->UGU | R->C | TTCAAATAAGTCGTGTTTTC | GTGGTTTTCCCATCTTACAA | 0.997 |
| *ccmB* | 1001 | 334 | 2 | CCA->CUA | P->L | AATGAATCGGTTAACCATTC | ATTAGGGAGCCCGGTATTGA | 0.965 |
| *ccmB* | 1013 | 338 | 2 | CCG->CUG | P->L | AACCATTCCATTAGGGAGCC | GGTATTGACTCTTCTGTGTG | 0.999 |
| *ccmB* | 1045 | 349 | 1 | CGU->UGU | R->C | TTCTGTGTGGTATTCACTCT | GTTCGGCTCTTGGAATCACA | 0.933 |
| *ccmB* | 1049 | 350 | 2 | UCG->UUG | S->L | GTGTGGTATTCACTCTCGTT | GGCTCTTGGAATCACATCCA | 0.991 |
| *ccmB* | 1088 | 363 | 2 | UCG->UUG | S->L | CAGCAGTGGTTGGAACAGCT | GCAAAATCCAAGCACTTCAC | 0.997 |
| *ccmB* | 1096 | 366 | 1 | CCA->UCA | P->S | GTTGGAACAGCTCGCAAAAT | CAAGCACTTCACCTACTTCA | 0.978 |
| *ccmB* | 1097 | 366 | 2 | CCA->CUA | P->L | TTGGAACAGCTCGCAAAATC | AAGCACTTCACCTACTTCAT | 0.978 |
| *ccmB* | 1106 | 369 | 2 | UCA->UUA | S->L | CTCGCAAAATCCAAGCACTT | ACCTACTTCATTGCCCCCAA | 0.99 |
| *ccmB* | 1115 | 372 | 2 | UCA->UUA | S->L | TCCAAGCACTTCACCTACTT | ATTGCCCCCAACCGTTTCTC | 0.979 |
| *ccmB* | 1123 | 375 | 1 | CCA->UCA | P->S | CTTCACCTACTTCATTGCCC | CAACCGTTTCTCGTACCTCT | 0.926 |
| *ccmB* | 1124 | 375 | 2 | CCA->CUA | P->L | TTCACCTACTTCATTGCCCC | AACCGTTTCTCGTACCTCTA | 0.992 |
| *ccmB* | 1133 | 378 | 2 | UCU->UUU | S->F | TTCATTGCCCCCAACCGTTT | TCGTACCTCTATTGAAACTG | 0.992 |
| *ccmB* | 1135 | 379 | 1 | CGU->UGU | R->C | CATTGCCCCCAACCGTTTCT | GTACCTCTATTGAAACTGAA | 0.976 |
| *ccmB* | 1169 | 390 | 2 | CCU->CUU | P->L | AACTGAATGGTTTCATGTTC | TTCATCGATTGGTTATTCTT | 0.999 |
| *ccmB* | 1172 | 391 | 2 | UCA->UUA | S->L | TGAATGGTTTCATGTTCCTT | ATCGATTGGTTATTCTTCTC | 0.985 |
| *ccmB* | 1175 | 392 | 2 | UCG->UUG | S->L | ATGGTTTCATGTTCCTTCAT | GATTGGTTATTCTTCTCTGT | 0.963 |
| *ccmB* | 1187 | 396 | 2 | UCU->UUU | S->F | TCCTTCATCGATTGGTTATT | TTCTCTGTTCGTATCTCTTT | 0.997 |
| *ccmB* | 1190 | 397 | 2 | UCU->UUU | S->F | TTCATCGATTGGTTATTCTT | TCTGTTCGTATCTCTTTTTC | 0.952 |
| *ccmB* | 1217 | 406 | 2 | UCG->UUG | S->L | CGTATCTCTTTTTCCAATTT | GGTCTCGATTAGTTCACAAG | 0.995 |
| *rps7* | 116 | 39 | 2 | CCA->CUA | P->L | TTATCAAACTTTTCATCGCC | AGCTCGAACTGAACGCGATG | 0.992 |
| *rps7* | 332 | 111 | 2 | UCA->UUA | S->L | GATAAGCTTAGAGAAATGTT | ATTTGCTGAGATACTGGATG | 0.996 |
| *cox3* | 245 | 82 | 2 | CCU->CUU | P->L | CAGAGTCGTACAATTAGGAC | TCGATATGGTTCTATTCCGT | 0.987 |
| *cox3* | 257 | 86 | 2 | UCU->UUU | S->F | ATTAGGACCTCGATATGGTT | TATTCCGTTCATCGTATCGG | 0.999 |
| *cox3* | 263 | 88 | 2 | CCG->CUG | P->L | ACCTCGATATGGTTCTATTC | GTTCATCGTATCGGAGGTTA | 0.999 |
| *cox3* | 289 | 97 | 1 | CUU->UUU | L->F | TCGTATCGGAGGTTATGTTC | TTTTTGCTCTTTTTCGGGCT | 1 |
| *cox3* | 298 | 100 | 1 | CUU->UUU | L->F | AGGTTATGTTCCTTTTTGCT | TTTTTCGGGCTTCTTCTCAT | 0.998 |
| *cox3* | 304 | 102 | 1 | CGG->UGG | R->W | TGTTCCTTTTTGCTCTTTTT | GGGCTTCTTCTCATTCTTCT | 0.991 |
| *cox3* | 311 | 104 | 2 | UCU->UUU | S->F | TTTTGCTCTTTTTCGGGCTT | TTCTCATTCTTCTTTGGCAC | 0.995 |
| *cox3* | 314 | 105 | 2 | UCU->UUU | S->F | TGCTCTTTTTCGGGCTTCTT | TCATTCTTCTTTGGCACCTA | 0.997 |
| *cox3* | 413 | 138 | 2 | CCU->CUU | P->L | AATCCCTTTTCTTAATACCC | TATTCTCCCTTCATCCGGAG | 0.993 |
| *cox3* | 422 | 141 | 2 | CCU->CUU | P->L | TCTTAATACCCCTATTCTCC | TTCATCCGGAGCTGCCGTAA | 0.993 |
| *cox3* | 512 | 171 | 2 | UCA->UUA | S->L | CGCTTTAGTAGCTACCGTTT | ACTGGCTCTAGTATCCACAG | 1 |
| *cox3* | 527 | 176 | 2 | UCC->UUC | S->F | CGTTTCACTGGCTCTAGTAT | CACAGGCTTTCAAGGAATGG | 1 |
| *cox3* | 602 | 201 | 2 | UCC->UUC | S->F | TATTTATGGTTCTACCTTTT | CTTAGCAACCGGCTTTCATG | 1 |
| *cox3* | 653 | 218 | 2 | UCG->UUG | S->L | GATTCTAGGTACTCTTTTCT | GATCATCTGTGGTATTCGCC | 0.997 |
| *cox3* | 764 | 255 | 2 | CCA->CUA | P->L | AGACGTGGTTTGGTTATTCC | ATTTGTCTCTATCTATTGGT | 1 |
| *cox2* | 38 | 13 | 2 | CCU->CUU | P->L | TCTATTCCCCACAATTGCTC | TTGTGATGCAGCGGAACCAT | 0.975 |
| *cox2* | 71 | 24 | 2 | UCU->UUU | S->F | GGAACCATGGCAATTAGGAT | TCAAGACGCAGCAACACCTA | 1 |
| *cox2* | 161 | 54 | 2 | UCA->UUA | S->L | TCTGATTTTGGTTTTCGTCT | ACGGATCTTAGTTCGCGCTT | 0.991 |
| *cox2* | 163 | 55 | 1 | CGG->UGG | R->W | TGATTTTGGTTTTCGTCTCA | GGATCTTAGTTCGCGCTTTA | 0.986 |
| *cox2* | 253 | 85 | 1 | CGG->UGG | R->W | GAACTACTATCGAGATTCTT | GGACCATATTTCCTAGTATA | 0.997 |
| *cox2* | 278 | 93 | 2 | CCG->CUG | P->L | CATATTTCCTAGTATAATCC | GATGTTCATTGCTATACCAT | 0.997 |
| *cox2* | 558 | 186 | 3 | UCC->UCU | S->S | ACCTATACCCCTAAATGCTC | CAGCATAGGAGCCTATGATT | 0.974 |
| *cox2* | 836 | 279 | 2 | UCU->UUU | S->F | AACTTCTTGTTCTTGCTCCT | TTCTTCGCTTCGGGTACGGA | 0.925 |
| *cox2* | 1378 | 460 | 1 | CGA->UGA | R->* | ACTCACTTTTGACAGTTATA | GATTCCAGAAGATGATCCAG | 0.999 |
| *cox2* | 1396 | 466 | 1 | CAG->UAG | Q->* | TACGATTCCAGAAGATGATC | AGAATTGGGTCAATCACGTT | 1 |
| *cox2* | 1411 | 471 | 1 | CAC->UAC | H->Y | TGATCCAGAATTGGGTCAAT | ACGTTTATTAGAAGTGGACA | 1 |
| *cox2* | 1516 | 506 | 1 | CAG->UAG | Q->* | TAGTTGGGCTGTACCTTCCT | AGGTGTCAAATGTGATGCTG | 1 |
| *cox2* | 1558 | 520 | 1 | CCU->UCU | P->S | ACCTGGTCGTTTAAATCAGA | CTCTATTTCGGTACAACGAG | 0.907 |
| *cox2* | 1567 | 523 | 1 | CGG->UGG | R->W | TTTAAATCAGACCTCTATTT | GGTACAACGAGAAGGAGTTT | 0.999 |
| *cox2* | 1633 | 545 | 1 | CGC->UGC | R->C | TGGAACGAATCATGCCTTTA | GCGTGCGCCCGGAAACATAG | 0.946 |
| *cox2* | 1889 | 630 | 2 | GCC->GUC | A->V | GTGGTTAGTGGCCAATAGCG | CATAGTTGATGGCATTCCTC | 0.913 |
| *cox2* | 2626 | 876 | 1 | CCU->UCU | P->S | CTATCGTCGTAGAAGCTGTT | CTAGGAAAGATTATGGTTCT | 0.999 |
| *cox2* | 2647 | 883 | 1 | CGG->UGG | R->W | CTAGGAAAGATTATGGTTCT | GGGTATCCAATCAATTAATC | 0.999 |
| *sdh4* | 39 | 13 | 3 | CCC->CCU | P->P | AGACGTGGTTTGGTTATTCC | ATTTGTCTCTATCTATTGGT | 1 |
| *cox1* | 196 | 66 | 1 | CCU->UCU | P->S | TTTTAATAACGGCTCACGCT | CTTTAATGATCTTTTTTATG | 1 |
| *cox1* | 197 | 66 | 2 | CCU->CUU | P->L | TTTAATAACGGCTCACGCTC | TTTAATGATCTTTTTTATGG | 0.996 |
| *cox1* | 242 | 81 | 2 | UCU->UUU | S->F | GCCGGCGATGATAGGTGGAT | TGGTAATTGGTCTGTTCCCA | 0.997 |
| *cox1* | 254 | 85 | 2 | UCU->UUU | S->F | AGGTGGATCTGGTAATTGGT | TGTTCCCATTCTGATAGGTG | 1 |
| *cox1* | 452 | 151 | 2 | UCU->UUU | S->F | AGCAGTTGATTTAGCAATTT | TAGTCTTCATCTATCTGGTG | 1 |
| *cox1* | 515 | 172 | 2 | UCC->UUC | S->F | AAATTTTATAACAACTATCT | CAACATGCGTGGACCCGGAA | 1 |
| *cox1* | 551 | 184 | 2 | UCA->UUA | S->L | CGGAATGACTATGCATAGAT | ACCCCTATTTGTGTGGTCCG | 1 |
| *cox1* | 590 | 197 | 2 | CCA->CUA | P->L | CGTTCTAGTGACAGCATTCC | ACTTTTATTATCACTTCCGG | 0.999 |
| *cox1* | 668 | 223 | 2 | UCU->UUU | S->F | AAACTTTAATACAACCTTTT | TGACCCCGCAGGAGGAGGGG | 0.973 |
| *cox1* | 715 | 239 | 1 | CGG->UGG | R->W | TATTATACCAGCATCTCTTT | GGTTCTTCGGTCATCCAGAG | 0.997 |
| *cox1* | 746 | 249 | 2 | CCC->CUC | P->L | TCATCCAGAGGTGTATATTC | CATTCTGCCTGGATTCGGTA | 0.999 |
| *cox1* | 860 | 287 | 2 | UCU->UUU | S->F | CAGTATAGGTGTTCTTGGAT | TCTTGTTCGGGCTCATCATA | 0.998 |
| *cox1* | 868 | 290 | 1 | CGG->UGG | R->W | GTGTTCTTGGATCTCTTGTT | GGGCTCATCATATGTTTACT | 0.992 |
| *cox1* | 1079 | 360 | 2 | CCG->CUG | P->L | AGGACTCACTGGAATAGTCC | GGCAAATTCAGGGCTAGACA | 0.996 |
| *cox1* | 1124 | 375 | 2 | GCG->GUG | A->V | TCTACACGATACTTATTATG | GGTTGCACATTTCCATTATG | 0.998 |
| *cox1* | 1186 | 396 | 1 | CAC->UAC | H->Y | TTGCTTTATTTGCAGGATTT | ACTATTGGGTGGGTAAAATC | 1 |
| *cox1* | 1259 | 420 | 2 | UCU->UUU | S->F | AATCCATTTTTGGATCACTT | TTTCGGGGTTAATCCGACCT | 1 |
| *cox1* | 1274 | 425 | 2 | CCG->CUG | P->L | CACTTCTTTCGGGGTTAATC | GACCTTCTTTCCCATGCATT | 0.998 |
| *cox1* | 1402 | 468 | 1 | CUU->UUU | L->F | ATATATCCGTAGTTGGGATT | TTCGTTTCTTCGTGGTCGTA | 0.998 |
| *cox1* | 1405 | 469 | 1 | CGU->UGU | R->C | TATCCGTAGTTGGGATTCTT | GTTTCTTCGTGGTCGTAACA | 0.996 |
| *cox1* | 1433 | 478 | 2 | UCA->UUA | S->L | CGTGGTCGTAACAATCACTT | AAGCAGTGGAAAGAACAAAA | 0.999 |
| *cox1* | 1499 | 500 | 2 | CCG->CUG | P->L | TGAACAGAATTCAACCACAC | GGAATGGATGATACAAAGTC | 0.996 |
| *cob* | 112 | 38 | 1 | CCG->UCG | P->S | GTTATTGGTGGGGGTTCGGT | CGTTAGCGGGTATTTGCTTA | 0.98 |
| *cob* | 280 | 94 | 1 | CUC->UUC | L->F | CTAATGGGGCAAGTATGTTT | TCATTGTGGTTTACCTTCAT | 1 |
| *cob* | 319 | 107 | 1 | CAU->UAU | H->Y | ATCTTTTTCGTGGTCTATAT | ATGCGAGTTATAGCAGCCCT | 0.993 |
| *cob* | 352 | 118 | 1 | CGG->UGG | R->W | GCAGCCCTAGGGAATTTGTT | GGTGTCTCGGAGTTGTAATA | 1 |
| *cob* | 401 | 134 | 2 | ACA->AUA | T->I | AATGATTGTGACAGCTTTTA | AGGATACGTACCACCTTGGG | 0.998 |
| *cob* | 413 | 138 | 2 | CCA->CUA | P->L | AGCTTTTACAGGATACGTAC | ACCTTGGGGTCAGATGAGCT | 1 |
| *cob* | 562 | 188 | 1 | CAU->UAU | H->Y | ATCGTTTTTTTAGTCTTCAT | ATTTACTCCCCCTTATTTTA | 0.996 |
| *cob* | 574 | 192 | 1 | CUU->UUU | L->F | GTCTTCATCATTTACTCCCC | TTATTTTAGTAGGCGCCAGT | 0.996 |
| *cob* | 674 | 225 | 2 | UCU->UUU | S->F | AGAGATGGATAAAATTGCTT | TTACCCTTATTTTTATGTAA | 1 |
| *cob* | 709 | 237 | 1 | CGG->UGG | R->W | ATGTAAAGGATCTAGTAGGT | GGGTAGCTTCTGCTATCTTT | 0.998 |
| *cob* | 719 | 240 | 2 | UCU->UUU | S->F | TCTAGTAGGTCGGGTAGCTT | TGCTATCTTTTCTTCCATTT | 0.996 |
| *cob* | 731 | 244 | 2 | UCU->UUU | S->F | GGTAGCTTCTGCTATCTTTT | TTCCATTTGGATTTTTTATG | 0.989 |
| *cob* | 802 | 268 | 1 | CCC->UCC | P->S | ATATACCTGCTAATCCGATG | CCACCCCGCCTCATATTGTG | 1 |
| *cob* | 847 | 283 | 1 | CAU->UAU | H->Y | AATGGTATTTCCTACCGATC | ATGCCATTCTTCGTAGTATA | 0.999 |
| *cob* | 902 | 301 | 2 | CCU->CUU | P->L | GGGTGTAGCCGCAATAGCAC | TGTTTTTATATGTCTGTTGG | 0.976 |
| *cob* | 976 | 326 | 1 | CAC->UAC | H->Y | GTTCAAGTTTTCGCCCGATT | ACCAAGGAATATTTTGGTTG | 0.999 |
| *cob* | 1078 | 360 | 1 | CCU->UCU | P->S | TTACTATTGGACAAATTTCT | CTTTAGTTTTCTTCTTGTTC | 0.998 |
| *ccmFN* | 38 | 13 | 2 | CCG->CUG | P->L | GTTTCATTATTCGTTATTTC | GGGTCTTTTCCTTGCATTCA | 0.92 |
| *ccmFN* | 137 | 46 | 2 | UCG->UUG | S->L | TCTTTCTTTCCTTGGTCTTT | GTTCCGTCATATTCCTAATA | 0.981 |
| *ccmFN* | 142 | 48 | 1 | CGU->UGU | R->C | CTTTCCTTGGTCTTTCGTTC | GTCATATTCCTAATAACTTA | 0.987 |
| *ccmFN* | 151 | 51 | 1 | CCU->UCU | P->S | GTCTTTCGTTCCGTCATATT | CTAATAACTTATCCAATTAC | 0.99 |
| *ccmFN* | 248 | 83 | 2 | UCA->UUA | S->L | TCATGAGGGTAGTATTTTAT | ATGGTGTCGGATCCCAAGTT | 0.999 |
| *ccmFN* | 256 | 86 | 1 | CGG->UGG | R->W | GTAGTATTTTATCATGGTGT | GGATCCCAAGTTTTTATGGA | 0.999 |
| *ccmFN* | 263 | 88 | 2 | CCA->CUA | P->L | TTTATCATGGTGTCGGATCC | AAGTTTTTATGGATTTTTTC | 0.996 |
| *ccmFN* | 372 | 124 | 3 | UUC->UUU | F->F | TATTCCTTTGTCTCGAACTT | GTGAAGAACTCTATCCTATC | 0.961 |
| *ccmFN* | 707 | 236 | 2 | CCU->CUU | P->L | CATTGCTTTGTTTTTCTCTC | TTTCCTATCAGCTAGTTCCG | 0.999 |
| *ccmFN* | 716 | 239 | 2 | UCA->UUA | S->L | GTTTTTCTCTCCTTTCCTAT | AGCTAGTTCCGATCCTTTTC | 0.995 |
| *ccmFN* | 776 | 259 | 2 | UCA->UUA | S->L | TACCGAACCGCTTGCAGAAT | AAATCCTGTTCCACAAGATC | 0.981 |
| *ccmFN* | 788 | 263 | 2 | CCA->CUA | P->L | TGCAGAATCAAATCCTGTTC | ACAAGATCCTATATCAGCTA | 0.999 |
| *ccmFN* | 803 | 268 | 2 | UCA->UUA | S->L | TGTTCCACAAGATCCTATAT | AGCTATACATCCTCCTTGCA | 0.991 |
| *ccmFN* | 946 | 316 | 1 | CGC->UGC | R->C | AAAAGAATGGAAGGCTGCTT | GCTCTGCTGGATGCGTCGGA | 0.957 |
| *ccmFN* | 1292 | 431 | 2 | CCA->CUA | P->L | TTTTACTGTGGGCATCTTAC | AGGAAGTTGGTGGGCTCATC | 0.998 |
| *ccmFN* | 1309 | 437 | 1 | CAU->UAU | H->Y | TACCAGGAAGTTGGTGGGCT | ATCATGAATTAGGTCGGGGT | 0.997 |
| *ccmFN* | 1324 | 442 | 1 | CGG->UGG | R->W | GGGCTCATCATGAATTAGGT | GGGGTGGCTGGTGGTTTCGG | 0.999 |
| *ccmFN* | 1342 | 448 | 1 | CGG->UGG | R->W | GTCGGGGTGGCTGGTGGTTT | GGGATCCCGTAGAAAATGCT | 0.999 |
| *ccmFN* | 1375 | 459 | 1 | CGG->UGG | R->W | AAAATGCTTCTTTTATACCT | GGGTATTAGCCACAGCTCGT | 1 |
| *ccmFN* | 1393 | 465 | 1 | CGU->UGU | R->C | CTCGGGTATTAGCCACAGCT | GTATTCATTCAGTAATTCTA | 0.996 |
| *ccmFN* | 1456 | 486 | 1 | CUU->UUU | L->F | TGTTTATTAATATTGTTACT | TTCCATGCTGTGTCTCAGGA | 0.991 |
| *ccmFN* | 1460 | 487 | 2 | CCA->CUA | P->L | TATTAATATTGTTACTCTTC | ATGCTGTGTCTCAGGAACCT | 0.989 |
| *ccmFN* | 1472 | 491 | 2 | UCA->UUA | S->L | TACTCTTCCATGCTGTGTCT | AGGAACCTTTTCAATACGGT | 1 |
| *ccmFN* | 1507 | 503 | 1 | CCC->UCC | P->S | TACGGTCCGGATTGCTAGCT | CCGTTCATAGTTTTGCTACA | 0.999 |
| *matR* | 25 | 9 | 1 | CCC->UCC | P->S | TACTCGAATCCATTTCCGAT | CCGAGTTTCCAGACACATCG | 0.933 |
| *matR* | 175 | 59 | 1 | CCA->UCA | P->S | TCGACCGACATCGACTCATC | CAATCTTTAAGGAAGAGATC | 0.994 |
| *matR* | 308 | 103 | 2 | CCG->CUG | P->L | TGTACTACTATCGGCCCTAC | GGGCAACATCTACCTACACA | 0.973 |
| *matR* | 395 | 132 | 2 | UCG->UUG | S->L | GATTGTTCAGAGAATCAGAT | GGTTCTATTAAGGACAGGTC | 0.973 |
| *matR* | 887 | 296 | 2 | UCA->UUA | S->L | CGCGCGATATGCCGACGACT | ACTACTGGGAATTGTAGGTG | 1 |
| *matR* | 1643 | 548 | 2 | UCC->UUC | S->F | CGGAGACATCGAAAATTGGT | CGCGGGCATCGCGATAAGTC | 0.939 |
| *matR* | 1664 | 555 | 2 | CCU->CUU | P->L | CGCGGGCATCGCGATAAGTC | TCTGTCCTACTACAGGTGCC | 0.999 |
| *matR* | 1684 | 562 | 1 | CGC->UGC | R->C | CTCTGTCCTACTACAGGTGC | GCGACAACCTTTACCAAGTC | 1 |
| *matR* | 1698 | 566 | 3 | UAC->UAU | Y->Y | AGGTGCCGCGACAACCTTTA | CAAGTCCGAACTATTGTCGA | 0.99 |
| *matR* | 1720 | 574 | 1 | CAC->UAC | H->Y | AAGTCCGAACTATTGTCGAC | ACCAGATCCGCTGGTCTGCA | 0.997 |
| *matR* | 1790 | 597 | 2 | CCA->CUA | P->L | CTCGGCGCGGAATATAATCC | AAAGTACTCCAAAGACTCTA | 0.998 |
| *matR* | 1808 | 603 | 2 | UCU->UUU | S->F | CCCAAAGTACTCCAAAGACT | TAATATAGTGAAAAAAGAAG | 0.994 |
| *nad4L* | 2 | 1 | 2 | ACG->AUG | T->M | NNNNNNNNNNNNNNNNNNNA | GGATCCTATCAAATATTTCA | 0.914 |
| *nad4L* | 41 | 14 | 2 | UCU->UUU | S->F | CACATTTTCTATGATCATTT | TATTTTAGGTATTCGGGGAA | 0.994 |
| *nad4L* | 55 | 19 | 1 | CGG->UGG | R->W | TCATTTCTATTTTAGGTATT | GGGGAATCCTCCTTAATAGA | 0.996 |
| *nad4L* | 86 | 29 | 2 | CCU->CUU | P->L | CCTTAATAGACGAAATATTC | TATTATGTTAATGCCAATTG | 0.999 |
| *nad4L* | 100 | 34 | 1 | CCA->UCA | P->S | ATATTCCTATTATGTTAATG | CAATTGAATCAATGTTATTA | 0.999 |
| *nad4L* | 110 | 37 | 2 | UCA->UUA | S->L | TATGTTAATGCCAATTGAAT | AATGTTATTAGCTGTGAATT | 0.999 |
| *nad4L* | 131 | 44 | 2 | UCG->UUG | S->L | AATGTTATTAGCTGTGAATT | GAACTTTTTGGTATTTTCCG | 1 |
| *nad4L* | 158 | 53 | 2 | UCG->UUG | S->L | TTTGGTATTTTCCGTTTCTT | GGATGATATGATGGGTCAAT | 1 |
| *nad4L* | 179 | 60 | 2 | UCA->UUA | S->L | GGATGATATGATGGGTCAAT | ATTTGCTTCATTGGTTCCAA | 1 |
| *nad4L* | 188 | 63 | 2 | UCA->UUA | S->L | GATGGGTCAATCATTTGCTT | ATTGGTTCCAACGGTGGCAG | 0.999 |
| *nad4L* | 196 | 66 | 1 | CCA->UCA | P->S | AATCATTTGCTTCATTGGTT | CAACGGTGGCAGCAGCGGAA | 0.946 |
| *nad4L* | 197 | 66 | 2 | CCA->CUA | P->L | ATCATTTGCTTCATTGGTTC | AACGGTGGCAGCAGCGGAAT | 0.974 |
| *nad4L* | 281 | 94 | 2 | UCU->UUU | S->F | AGGGACTATTGCTGTAGAAT | TATTAATAGCATTCAAGGTT | 0.999 |
| *mttB* | 16 | 6 | 1 | CAU->UAU | H->Y | NNNNNATATCCTATGAATTT | ATTTAGCACCGGAAACTTTT | 0.979 |
| *mttB* | 25 | 9 | 1 | CCG->UCG | P->S | CCTATGAATTTCATTTAGCA | CGGAAACTTTTCTAGGAGAA | 0.991 |
| *mttB* | 26 | 9 | 2 | CCG->CUG | P->L | CTATGAATTTCATTTAGCAC | GGAAACTTTTCTAGGAGAAG | 0.998 |
| *mttB* | 59 | 20 | 2 | UCC->UUC | S->F | AGGAGAAGTTCGAATTCGTT | CGTTCGGGTATTGATCGGTC | 0.927 |
| *mttB* | 64 | 22 | 1 | CGG->UGG | R->W | AAGTTCGAATTCGTTCCGTT | GGGTATTGATCGGTCTTGGT | 0.995 |
| *mttB* | 100 | 34 | 1 | CGU->UGU | R->C | TTGGTTTGACATGGTTTACG | GTTACTGGTTCCCGGAAGAG | 0.999 |
| *mttB* | 112 | 38 | 1 | CCG->UCG | P->S | GGTTTACGCGTTACTGGTTC | CGGAAGAGTTAATATCTCCA | 0.998 |
| *mttB* | 128 | 43 | 2 | UCU->UUU | S->F | GTTCCCGGAAGAGTTAATAT | TCCATTAGCTAAACCCTTTC | 0.996 |
| *mttB* | 131 | 44 | 2 | CCA->CUA | P->L | CCCGGAAGAGTTAATATCTC | ATTAGCTAAACCCTTTCTTA | 0.985 |
| *mttB* | 178 | 60 | 1 | CGU->UGU | R->C | CTTTGGACTCGTATTTTGTT | GTACACAATTAACGGAGGCC | 1 |
| *mttB* | 202 | 68 | 1 | CCG->UCG | P->S | CACAATTAACGGAGGCCTTC | CGACATATATTGCTTCGTCT | 0.994 |
| *mttB* | 262 | 88 | 1 | CAU->UAU | H->Y | TCGTCTTTCCCTTAATAAGT | ATCAAATTTGGTGCTTTTTG | 0.991 |
| *mttB* | 328 | 110 | 1 | CUC->UUC | L->F | GGACGAAATACAATCGATTC | TCCATTTAAGTGGTTCTCGC | 0.984 |
| *mttB* | 331 | 111 | 1 | CAU->UAU | H->Y | CGAAATACAATCGATTCCTC | ATTTAAGTGGTTCTCGCTTC | 0.983 |
| *mttB* | 344 | 115 | 2 | UCU->UUU | S->F | ATTCCTCCATTTAAGTGGTT | TCGCTTCTCCTTGTTCCTGT | 0.933 |
| *mttB* | 346 | 116 | 1 | CGC->UGC | R->C | TCCTCCATTTAAGTGGTTCT | GCTTCTCCTTGTTCCTGTTC | 0.999 |
| *mttB* | 373 | 125 | 1 | CCU->UCU | P->S | CCTTGTTCCTGTTCCTAACT | CTCCACGGGTAGTTCCCAAT | 0.966 |
| *mttB* | 374 | 125 | 2 | CCU->CUU | P->L | CTTGTTCCTGTTCCTAACTC | TCCACGGGTAGTTCCCAATG | 0.987 |
| *mttB* | 407 | 136 | 2 | CCA->CUA | P->L | TCCCAATGTTTGGCACTTTC | ATACTTAGTGGGTGCAACAT | 0.996 |
| *mttB* | 497 | 166 | 2 | UCG->UUG | S->L | TATGTTTACTGCTCGTATTT | GTTCATTCCATCGGTATGCT | 0.97 |
| *mttB* | 505 | 169 | 1 | CCA->UCA | P->S | CTGCTCGTATTTCGTTCATT | CATCGGTATGCTCCCAGGTA | 0.956 |
| *mttB* | 541 | 181 | 1 | CGU->UGU | R->C | AGGTACCTGTAATTGTGATC | GTTTGCCAGAACCAAGGGGT | 0.997 |
| *mttB* | 548 | 183 | 2 | CCA->CUA | P->L | TGTAATTGTGATCCGTTTGC | AGAACCAAGGGGTCTTTCTG | 0.999 |
| *mttB* | 554 | 185 | 2 | CCA->CUA | P->L | TGTGATCCGTTTGCCAGAAC | AAGGGGTCTTTCTGTGGGAA | 0.99 |
| *mttB* | 610 | 204 | 1 | CCG->UCG | P->S | GTCGTTTTTTGGTGGTTTTT | CGCTTATCACAGCAGCTCTT | 0.983 |
| *mttB* | 660 | 220 | 3 | AUC->AUU | I->I | CCCGATATCTGGTGCCAAAT | GTCGCCCGTTTCCTTATTTA | 0.973 |
| *mttB* | 667 | 223 | 1 | CGU->UGU | R->C | TCTGGTGCCAAATCGTCGCC | GTTTCCTTATTTATTATATA | 0.937 |
| *mttB* | 704 | 235 | 2 | UCU->UUU | S->F | TATAATAGAGTTGGCTATCT | TGTGGCATCGATTGTACAAG | 0.998 |
| *mttB* | 713 | 238 | 2 | UCG->UUG | S->L | GTTGGCTATCTCTGTGGCAT | GATTGTACAAGTTCGTGAAG | 0.998 |
| *mttB* | 797 | 266 | 2 | UCU->UUU | S->F | TACCGCCCCCCTTACTCTCT | TATTTTAAAGCCTGCGGGAA | 0.922 |
| *atp4* | 56 | 19 | 2 | ACA->AUA | T->I | GATGCTATTTGCTGCTATTA | ATCTTTTTGTGCATCAAGTT | 0.911 |
| *atp4* | 59 | 20 | 2 | UCU->UUU | S->F | GCTATTTGCTGCTATTACAT | TTTTTGTGCATCAAGTTCGA | 0.999 |
| *atp4* | 89 | 30 | 2 | UCA->UUA | S->L | ATCAAGTTCGAAGAATATCT | AATCTATAATGAAGAAATGA | 0.999 |
| *atp4* | 118 | 40 | 1 | CGU->UGU | R->C | ATGAAGAAATGATAGTAGCT | GTTGTTTTATAGGCTTTATC | 0.974 |
| *atp4* | 215 | 72 | 2 | UCG->UUG | S->L | CCAGGCTATTCAGGAAGAAT | GCAGCAATTCCCCAATCCTA | 0.998 |
| *atp4* | 227 | 76 | 2 | CCC->CUC | P->L | GGAAGAATCGCAGCAATTCC | CAATCCTAACGAAGTAGTTC | 0.999 |
| *atp4* | 248 | 83 | 2 | CCU->CUU | P->L | CAATCCTAACGAAGTAGTTC | TCCGGAATCCAATGAACAAC | 1 |
| *atp4* | 250 | 84 | 1 | CCG->UCG | P->S | ATCCTAACGAAGTAGTTCCT | CGGAATCCAATGAACAACAA | 0.903 |
| *atp4* | 251 | 84 | 2 | CCG->CUG | P->L | TCCTAACGAAGTAGTTCCTC | GGAATCCAATGAACAACAAC | 1 |
| *atp4* | 395 | 132 | 2 | UCA->UUA | S->L | CCGAAACCTAAATGTTAAGT | AGCAACACTTCCAAATGCCA | 0.999 |
| *atp4* | 407 | 136 | 2 | CCA->CUA | P->L | TGTTAAGTCAGCAACACTTC | AAATGCCACTTCTTCCCGTC | 0.993 |
| *atp4* | 416 | 139 | 2 | ACU->AUU | T->I | AGCAACACTTCCAAATGCCA | TTCTTCCCGTCGCATCCGTC | 0.987 |
| *atp1* | 1039 | 347 | 1 | CCC->UCC | P->S | ATATTCCCACCAATGTGATC | CCATTACTGATGGACAAATT | 0.999 |
| *atp1* | 1168 | 390 | 1 | CGC->UGC | R->C | TGAAAGCTATGAAACAAGTT | GCGGGAGTTTAAAACTGGAA | 0.999 |
| *atp1* | 1415 | 472 | 2 | CCA->CUA | P->L | TCAATATGAGAGAGCCATTC | AAGTAGTGTAAAACCAGAAT | 0.99 |
| *atp1* | 1490 | 497 | 2 | CCA->CUA | P->L | TAATGAGAAAATACGAGAAC | AGATGCTTTCTTAAAAAGCG | 0.973 |
| *ccmFC* | 38 | 13 | 2 | UCC->UUC | S->F | CTTTTTCTTTTTCATTACTT | CATGGTCGTGCCTCGTGGCA | 0.998 |
| *ccmFC* | 50 | 17 | 2 | CCU->CUU | P->L | CATTACTTCCATGGTCGTGC | TCGTGGCACGGCAGCACCCG | 1 |
| *ccmFC* | 52 | 18 | 1 | CGU->UGU | R->C | TTACTTCCATGGTCGTGCCT | GTGGCACGGCAGCACCCGTA | 0.984 |
| *ccmFC* | 103 | 35 | 1 | CCC->UCC | P->S | GGTTCGTCAGTAGAGATGTT | CCACGGGTGCCCCTTCTTCC | 0.997 |
| *ccmFC* | 119 | 40 | 2 | UCU->UUU | S->F | TGTTCCCACGGGTGCCCCTT | TTCCAATGGTACTATAATTC | 0.982 |
| *ccmFC* | 122 | 41 | 2 | UCC->UUC | S->F | TCCCACGGGTGCCCCTTCTT | CAATGGTACTATAATTCCTA | 0.999 |
| *ccmFC* | 146 | 49 | 2 | CCU->CUU | P->L | TGGTACTATAATTCCTATTC | TATCCCTTCATTCCCTCTTT | 0.992 |
| *ccmFC* | 151 | 51 | 1 | CCU->UCU | P->S | CTATAATTCCTATTCCTATC | CTTCATTCCCTCTTTTGGTC | 0.993 |
| *ccmFC* | 155 | 52 | 2 | UCA->UUA | S->L | AATTCCTATTCCTATCCCTT | ATTCCCTCTTTTGGTCTATC | 0.941 |
| *ccmFC* | 296 | 99 | 2 | UCA->UUA | S->L | AACTCGAGCTAGAAACGCCT | ATTTCGTTTCGTTCCCCTTC | 0.998 |
| *ccmFC* | 301 | 101 | 1 | CGU->UGU | R->C | GAGCTAGAAACGCCTCATTT | GTTTCGTTCCCCTTCTTCAT | 0.983 |
| *ccmFC* | 313 | 105 | 1 | CUU->UUU | L->F | CCTCATTTCGTTTCGTTCCC | TTCTTCATTTCATTATTCTT | 0.943 |
| *ccmFC* | 382 | 128 | 1 | CGU->UGU | R->C | AATCTTTATGCGGTGTGCTC | GTTTACTATTCTTTCGTACT | 0.984 |
| *ccmFC* | 397 | 133 | 1 | CGU->UGU | R->C | TGCTCCGTTTACTATTCTTT | GTACTCTATTCTCTTTACCA | 0.997 |
| *ccmFC* | 925 | 309 | 1 | CGU->UGU | R->C | AGATGGCTGATTCAATAATT | GTGCATAAGGGTAAGTAACT | 0.903 |
| *ccmFC* | 1798 | 600 | 1 | CUC->UUC | L->F | TCATTTACATGGACCCACTT | TCATTCCATTTGTGGAAATT | 0.996 |
