# Supplementary material for: Characterization and evolutionary insights into complete mitochondrial genome of Sedum sarmentosum within the family Crassulaceae
Source: Front Plant Sci. 2026 Feb 6;17:1710625. doi: 10.3389/fpls.2026.1710625 (PMC12920544; doi:10.3389/fpls.2026.1710625)
Supplement: Supplementary file 8 [file Table8.docx]

**Table S8 | Selection pressures analysis results of the 26 PCGs of *Sedum sarmentosum* versus its relatiives.**

| **Gene** | **S. portulacastrum** | | **F. aubertii** | | **T. tetragonoides** | | **Beta_macrocarpa** | **V. album** | | **Nepenthes_ventricosa_x_Nepenthes_alata** | | |  |
| --- | --- | --- | --- | --- | --- | --- | --- | --- | --- | --- | --- | --- | --- |
| *atp1* | | 0.142481 | | 0.172862 | | 0.151161 | | | 0.156541 | | 0.159472 | 0.151761 | |
| *atp4* | | 0.533987 | | 0.630121 | | 0.619913 | | | 0.526824 | | - | 0.471602 | |
| *atp6* | | 0.542392 | | 0.620227 | | 0.565698 | | | 0.548409 | | 0.212971 | 0.433018 | |
| *atp8* | | 0.629129 | | 0.528476 | | 0.510726 | | | 0.489164 | | - | 0.494533 | |
| *atp9* | | 0.0569551 | | 0.0314788 | | 0.049381 | | | 0.0595458 | | 0.104192 | 0.0725827 | |
| *ccmB* | | 1.16905 | | 0.793584 | | 1.16971 | | | 1.02024 | | 1.08141 | 1.10111 | |
| *ccmC* | | 0.956833 | | 0.930404 | | 0.948535 | | | 0.842844 | | 0.541553 | 1.10135 | |
| *ccmFC* | | 0.636855 | | 0.541019 | | 0.586314 | | | 0.572273 | | - | 0.500652 | |
| *ccmFN* | | 0.62309 | | 0.775494 | | 0.636012 | | | 0.678068 | | 0.235769 | 0.73041 | |
| *cox1* | | 0.190981 | | 0.275461 | | 0.181852 | | | 0.171286 | | 0.107767 | 0.347493 | |
| *cox2* | | 0.642377 | | 0.447061 | | 0.560386 | | | 0.344959 | | 0.242397 | 0.366337 | |
| *cox3* | | 0.525849 | | 0.450061 | | 0.535183 | | | 0.446253 | | 0.312687 | 0.64222 | |
| *nad2* | | 0.56403 | | 0.402301 | | 0.540594 | | | 0.464946 | | - | 0.429162 | |
| *nad3* | | 0.393141 | | 0.239017 | | 0.263876 | | | 0.401611 | | - | 0.360279 | |
| *nad4* | | 0.649936 | | 0.632824 | | 0.560878 | | | 0.70814 | | - | 0.695137 | |
| *nad4L* | | 0.427028 | | 0.387154 | | 0.52632 | | | 0.202125 | | - | 0.325419 | |
| *nad6* | | 0.195145 | | 0.214815 | | 0.203818 | | | 0.227564 | | - | 0.19883 | |
| *nad7* | | 0.838808 | | 0.933014 | | 0.978228 | | | 0.632129 | | - | 0.997832 | |
| *nad9* | | 0.499599 | | 0.38988 | | 0.468778 | | | 0.380456 | | - | 0.446462 | |
| *rpl10* | | - | | - | | - | | | - | | - | - | |
| *rpl16* | | - | | 0.38638 | | - | | | - | | - | - | |
| *rpl5* | | 0.523051 | | 0.319145 | | - | | | 0.444642 | | - | 0.467183 | |
| *rps12* | | 0.460005 | | 0.226664 | | 0.345968 | | | 0.249628 | | 0.25992 | 0.1917 | |
| *rps13* | | - | | 0.419817 | | - | | | 0.492841 | | - | 0.436136 | |
| *rps3* | | 3.04192 | | 0.563637 | | 3.24833 | | | 2.33099 | | - | 1.66012 | |
| *rps7* | | 0.500175 | | 0.421019 | | 0.50272 | | | 0.540733 | | - | 0.39858 | |

| **Gene** | **M. esculenta** | **M. oleifera** | **T. maclurei** | **S. glauca** | **S. plumbizincicola** | **P. lactiflora** | **R. crenulata** | **R. tangutica** | **S. album** |
| --- | --- | --- | --- | --- | --- | --- | --- | --- | --- |
| *atp1* | 0.184064 | 0.194119 | 0.220406 | 0.131307 | 0.157176 | 0.169754 | 0.225368 | 0.22383 | 0.202211 |
| *atp4* | 0.707 | 0.358359 | 0.678849 | 0.549252 | 0.643826 | 0.792448 | 0.399328 | 0.318554 | 0.83186 |
| *atp6* | 0.361338 | 0.491767 | 0.432429 | 0.505686 | 0.12727 | 0.278902 | 0.179608 | 0.443625 | 0.53741 |
| *atp8* | 0.488673 | 0.317991 | 0.562049 | 0.466015 | NA | 0.498539 | 0.49578 | 0.655296 | 0.53803 |
| *atp9* | 0.0213302 | 0.0749778 | 0.0336426 | 0.053686 | 0 | 0.100411 | - | 0 | 0.0770204 |
| *ccmB* | 1.01615 | 0.612622 | 0.846418 | 1.30474 | 0.368242 | 0.508597 | 0.237146 | 0.237146 | 0.717814 |
| *ccmC* | 1.02546 | 1.0052 | 1.23506 | 0.805749 | 0.351571 | 1.10232 | 0.35041 | 0.526239 | 1.70096 |
| *ccmFC* | 0.651367 | 0.650163 | 0.701546 | 0.507075 | 0.791372 | 0.601119 | 0.459175 | 0.417663 | 0.67041 |
| *ccmFN* | 0.649534 | 0.710171 | 0.716738 | 0.597658 | 0.418753 | 0.655409 | - | - | 0.670317 |
| *cox1* | - | 0.182064 | 0.212764 | 0.150327 | 0.0878465 | 0.192808 | 0.024844 | 0.024844 | 0.193606 |
| *cox2* | 0.267638 | 0.58353 | 0.384142 | 0.300082 | 0.277387 | 0.394871 | - | 0.335286 | 0.412492 |
| *cox3* | 0.497627 | 0.584982 | 0.583682 | 0.361922 | 0.649151 | 0.42692 | 0.467549 | 0.467549 | 0.687134 |
| *nad2* | 0.602076 | 0.79911 | 0.598176 | 0.370329 | NA | 0.203875 | 1.21412 | 1.14011 | 0.544978 |
| *nad3* | 1.1577 | 0.306066 | 0.670113 | 0.411155 | NA | 0.161295 | - | 0.304039 | 0.260402 |
| *nad4* | 0.43283 | 0.620723 | 0.55308 | 0.212755 | 0.698045 | 0.453863 | 0.356979 | 0.435769 | 0.687194 |
| *nad4L* | 0.510632 | 0.295453 | 0.143415 | 0.203928 | 0 | 0.140197 | 0 | 0.147846 | 0.302121 |
| *nad6* | 0.286166 | 0.262323 | 0.21885 | - | NA | 0.249443 | 0.30275 | 0.329536 | 0.260511 |
| *nad7* | 1.49147 | 1.02248 | 0.442361 | 0.825499 | 0.359466 | 0.530427 | 0.636595 | 0.636595 | 0.663362 |
| *nad9* | 0.390288 | 0.304353 | 0.382265 | 0.379291 | 0 | 0.361274 | 0 | 0.712028 | 0.526706 |
| *rpl10* | 0.652778 | 0.666358 | - | - | 0.500087 | - | 0.390762 | 0.390762 | 1.63159 |
| *rpl16* | 0.252708 | 0.41393 | - | - | NA | 0.559212 | - | 0.452892 | 0.58379 |
| *rpl5* | 0.716035 | 0.932915 | 0.644876 | 0.52839 | NA | 0.604453 | 0.628314 | 0.628314 | 0.67098 |
| *rps12* | 0.115722 | 0.164723 | 0.206631 | 0.210568 | 0.311344 | 0.160195 | - | 0 | 0.25197 |
| *rps13* | 0.363872 | 0.463762 | - | - | NA | - | 0.0717974 | 0.108309 | - |
| *rps3* | - | 1.44666 | 0.540596 | 2.91017 | 1.07391 | - | - | - | 1.60416 |
| *rps7* | - | 0.355292 | 0.52125 | 0.52647 | 0.312857 | - | - | 0.0767013 | 0.353034 |

| **Gene** | R. palmatum | R. juparensis | P. suffruticosa | M. laxiflora | M. crystallinum | R. rosea | S. ramosissima | T. austromongolica | R. webbianum | M. ussuriense |
| --- | --- | --- | --- | --- | --- | --- | --- | --- | --- | --- |
| *atp1* | 0.188143 | 0.22383 | 0.156635 | 0.15385 | - | 0.237248 | 0.140522 | 0.14558 | 0.188143 | 0.169104 |
| *atp4* | 0.654072 | 0.318554 | 0.792448 | 0.570293 | - | 0.3974 | 0.588193 | 0.593902 | 0.654072 | 1.17905 |
| *atp6* | 0.571138 | 0.129041 | 0.278803 | 0.412854 | - | 0.129041 | 0.50804 | 0.374814 | 0.571138 | 0.528696 |
| *atp8* | 0.422942 | 0.799869 | 0.498539 | 0.543439 | 0.448454 | 0.49578 | 0.431423 | 0.56407 | 0.422942 | 0.300287 |
| *atp9* | 0.0311363 | - | 0.100411 | 0.0351721 | 0.0173824 | 0 | 0.03444 | 0.0248152 | 0.0311363 | - |
| *ccmB* | 0.793584 | 0.237146 | 0.508597 | 0.789449 | 0.900127 | 0.237146 | 1.11725 | 0.767173 | 0.793584 | 0.726334 |
| *ccmC* | 1.01525 | 0.526239 | 0.867448 | 0.970284 | 0.857675 | 0.527686 | 1.04508 | 1.02196 | 0.978641 | 1.04999 |
| *ccmFC* | 0.545587 | 0.420777 | 0.548478 | 0.584766 | - | 0.666416 | 0.509127 | 0.534317 | 0.520203 | 0.472931 |
| *ccmFN* | 0.819368 | 0.618639 | 0.731308 | 0.533069 | - | 0.627215 | 0.683604 | 0.533354 | 0.803838 | 0.632774 |
| *cox1* | 0.272616 | 0.024844 | 0.192808 | 0.26646 | - | 0.024844 | 0.15431 | 0.265712 | 0.277583 | 0.0889229 |
| *cox2* | 0.452234 | 0.206582 | 0.394871 | 0.273069 | 0.6221 | 0.206582 | 0.298921 | 0.302991 | 0.452234 | 0.258067 |
| *cox3* | 0.543998 | 0.467549 | 0.462936 | 0.338719 | 0.330009 | 0.397676 | 0.444499 | 0.373413 | 0.408836 | 0.550665 |
| *nad2* | 0.425195 | 1.21412 | 0.203875 | - | 0.611588 | NA | 0.380705 | 0.537952 | 0.425195 | 0.18682 |
| *nad3* | 0.237959 | 0.611843 | 0.213884 | 0.875885 | - | 0.304039 | 0.367459 | 0.844658 | 0.237959 | 0.224953 |
| *nad4* | 0.586251 | 0.35687 | 0.512493 | 0.47409 | 0.31187 | 0.689955 | 0.611071 | 0.462399 | 0.604015 | 0.322411 |
| *nad4L* | 0.387154 | 0 | 0.140197 | 0.402237 | - | 0 | 0.23853 | 0.402237 | 0.387154 | 0.175549 |
| *nad6* | 0.192449 | 0.329536 | 0.249443 | 0.185528 | 0.19472 | 0.329536 | 0.216502 | 0.178957 | 0.192449 | 0.238122 |
| *nad7* | 0.831119 | 0.636595 | 0.530427 | 0.703996 | - | 0.636595 | 0.817312 | 0.769107 | 0.933014 | 0.583134 |
| *nad9* | 0.446661 | 0.457015 | 0.361274 | 0.502784 | 0.383614 | 0.457015 | 0.403225 | 0.387336 | 0.446661 | 0.466573 |
| *rpl10* | - | 0.390762 | - | - | - | 0.390762 | - | - | - | 0.590745 |
| *rpl16* | 0.353046 | - | 0.550236 | - | - | 0.297299 | - | - | - | - |
| *rpl5* | 0.332617 | 0.628314 | 0.604453 | 0.720454 | 0.394486 | 0.628314 | 0.475166 | 0.69781 | 0.353716 | 0.704281 |
| *rps12* | 0.200574 | 0 | 0.186348 | 0.257992 | - | 0 | 0.199633 | 0.225026 | 0.226664 | 0.213026 |
| *rps13* | 0.434243 | 0.144397 | - | 0.523665 | - | 0.0717974 | 0.48211 | 0.402737 | 0.434243 | 0.460573 |
| *rps3* | 0.579353 | - | - | 0.562753 | - | - | 1.53121 | 0.444897 | 0.590532 | 0.61578 |
| *rps7* | 0.49329 | 0.0767013 | 0.298226 | 0.460839 | 0.322901 | 0.152672 | 0.430725 | 0.453013 | 0.49329 | 0.484377 |
