# Supplementary material for: Characterization and evolutionary insights into complete mitochondrial genome of Sedum sarmentosum within the family Crassulaceae
Source: Front Plant Sci. 2026 Feb 6;17:1710625. doi: 10.3389/fpls.2026.1710625 (PMC12920544; doi:10.3389/fpls.2026.1710625)
Supplement: Supplementary file 9 [file Table9.docx]

**Table S9 | Collinearity analysis results of the mitochondrial genome of *Sedum sarmentosum* and its closely related species.**

| **query** | **database** | **identity (%)** | **alignment** | **mismatch** | **gap** | **q. start** | **q. end** | **d. start** | **d. end** | **e-value** | **score** |
| --- | --- | --- | --- | --- | --- | --- | --- | --- | --- | --- | --- |
| mtChr1 | *NC_070189.1* | 92.088 | 4171 | 172 | 59 | 137756 | 141827 | 140532 | 144643 | 0 | 5729 |
| mtChr1 | *NC_070189.1* | 95.984 | 3212 | 99 | 16 | 35992 | 39190 | 31739 | 34933 | 0 | 5190 |
| mtChr1 | *NC_070189.1* | 92.175 | 2211 | 109 | 25 | 146402 | 148561 | 152746 | 150549 | 0 | 3066 |
| mtChr1 | *NC_070189.1* | 92.185 | 2201 | 98 | 23 | 154577 | 156728 | 3471 | 1296 | 0 | 3044 |
| mtChr1 | *NC_070189.1* | 91.633 | 2247 | 110 | 33 | 123387 | 125592 | 114978 | 112769 | 0 | 3037 |
| mtChr1 | *NC_070189.1* | 96.305 | 1678 | 53 | 4 | 23324 | 24992 | 5361 | 3684 | 0 | 2747 |
| mtChr1 | *NC_070189.1* | 90.923 | 2016 | 137 | 20 | 94423 | 96395 | 88540 | 90552 | 0 | 2667 |
| mtChr1 | *NC_070189.1* | 94.104 | 1679 | 56 | 19 | 102651 | 104312 | 107110 | 108762 | 0 | 2512 |
| mtChr1 | *NC_070189.1* | 95.567 | 1534 | 65 | 3 | 114775 | 116307 | 172657 | 171126 | 0 | 2453 |
| mtChr1 | *NC_070189.1* | 97.445 | 1409 | 24 | 9 | 75404 | 76803 | 95100 | 93695 | 0 | 2392 |
| mtChr1 | *NC_070189.1* | 96.622 | 1421 | 34 | 7 | 48796 | 50207 | 13346 | 11931 | 0 | 2346 |
| mtChr1 | *NC_070189.1* | 96.762 | 1359 | 29 | 6 | 76932 | 78289 | 93566 | 92222 | 0 | 2252 |
| mtChr1 | *NC_070189.1* | 92.192 | 1524 | 72 | 15 | 135270 | 136761 | 137285 | 138793 | 0 | 2111 |
| mtChr1 | *NC_070189.1* | 95.628 | 1258 | 35 | 12 | 121910 | 123156 | 116337 | 115089 | 0 | 2001 |
| mtChr1 | *NC_070189.1* | 89.271 | 1659 | 92 | 34 | 120291 | 121903 | 118035 | 116417 | 0 | 1999 |
| mtChr1 | *NC_070189.1* | 91.054 | 1509 | 55 | 24 | 100693 | 102153 | 105545 | 107021 | 0 | 1965 |
| mtChr1 | *NC_070189.1* | 95.789 | 1211 | 49 | 1 | 46506 | 47714 | 48079 | 49289 | 0 | 1953 |
| mtChr1 | *NC_070189.1* | 93.379 | 1012 | 40 | 13 | 31158 | 32151 | 97343 | 98345 | 0 | 1472 |
| mtChr1 | *NC_070189.1* | 93.083 | 1012 | 40 | 9 | 119297 | 120297 | 119172 | 118180 | 0 | 1454 |
| mtChr1 | *NC_070189.1* | 93.971 | 962 | 42 | 9 | 81753 | 82705 | 65142 | 66096 | 0 | 1441 |
| mtChr1 | *NC_070189.1* | 95.765 | 850 | 36 | 0 | 131255 | 132104 | 124924 | 124075 | 0 | 1371 |
| mtChr1 | *NC_070189.1* | 90.129 | 1084 | 60 | 26 | 41438 | 42480 | 157106 | 158183 | 0 | 1365 |
| mtChr1 | *NC_070189.1* | 91.304 | 1012 | 58 | 15 | 14961 | 15956 | 69863 | 70860 | 0 | 1354 |
| mtChr1 | *NC_070189.1* | 93.096 | 927 | 47 | 6 | 32359 | 33268 | 98561 | 99487 | 0 | 1341 |
| mtChr1 | *NC_070189.1* | 95.119 | 840 | 34 | 3 | 61703 | 62535 | 178917 | 178078 | 0 | 1317 |
| mtChr1 | *NC_070189.1* | 95.528 | 805 | 36 | 0 | 116347 | 117151 | 40857 | 41661 | 0 | 1288 |
| mtChr1 | *NC_070189.1* | 97.312 | 744 | 15 | 4 | 69044 | 69782 | 61389 | 60646 | 0 | 1258 |
| mtChr1 | *NC_070189.1* | 94.937 | 790 | 37 | 3 | 10368 | 11154 | 38052 | 38841 | 0 | 1234 |
| mtChr1 | *NC_070189.1* | 92.627 | 868 | 35 | 10 | 17148 | 18013 | 45471 | 44631 | 0 | 1221 |
| mtChr1 | *NC_070189.1* | 89.76 | 957 | 50 | 23 | 104354 | 105293 | 108945 | 109870 | 0 | 1181 |
| mtChr1 | *NC_070189.1* | 92.692 | 821 | 39 | 12 | 69860 | 70678 | 60340 | 59539 | 0 | 1164 |
| mtChr1 | *NC_070189.1* | 97.028 | 673 | 15 | 2 | 48126 | 48793 | 14051 | 13379 | 0 | 1127 |
| mtChr1 | *NC_070189.1* | 91.607 | 834 | 34 | 5 | 132866 | 133671 | 87196 | 86371 | 0 | 1120 |
| mtChr1 | *NC_070189.1* | 88.506 | 957 | 64 | 27 | 50873 | 51799 | 154671 | 155611 | 0 | 1116 |
| mtChr1 | *NC_070189.1* | 88.988 | 899 | 81 | 12 | 113562 | 114454 | 111597 | 110711 | 0 | 1096 |
| mtChr1 | *NC_070189.1* | 93.683 | 744 | 19 | 9 | 148569 | 149312 | 150457 | 149742 | 0 | 1088 |
| mtChr1 | *NC_070189.1* | 91.86 | 774 | 42 | 11 | 27871 | 28629 | 51386 | 50619 | 0 | 1061 |
| mtChr1 | *NC_070189.1* | 94.659 | 674 | 23 | 5 | 40744 | 41415 | 156381 | 157043 | 0 | 1033 |
| mtChr1 | *NC_070189.1* | 93.676 | 680 | 34 | 2 | 18255 | 18925 | 153091 | 153770 | 0 | 1009 |
| mtChr1 | *NC_070189.1* | 93.676 | 680 | 34 | 2 | 105377 | 106047 | 153091 | 153770 | 0 | 1009 |
| mtChr1 | *NC_070189.1* | 90.079 | 756 | 53 | 9 | 33276 | 34027 | 99544 | 100281 | 0 | 961 |
| mtChr1 | *NC_070189.1* | 92.342 | 666 | 50 | 1 | 88151 | 88816 | 20168 | 19504 | 0 | 946 |
| mtChr1 | *NC_070189.1* | 92.615 | 650 | 29 | 7 | 16482 | 17119 | 72161 | 72803 | 0 | 917 |
| mtChr1 | *NC_070189.1* | 90.506 | 632 | 19 | 13 | 92578 | 93209 | 23809 | 23219 | 0 | 797 |
| mtChr1 | *NC_070189.1* | 93.449 | 519 | 34 | 0 | 87144 | 87662 | 126036 | 125518 | 0 | 771 |
| mtChr1 | *NC_070189.1* | 84.918 | 789 | 59 | 15 | 90969 | 91756 | 39456 | 40185 | 0 | 743 |
| mtChr1 | *PQ580749.1* | 92.477 | 4174 | 188 | 54 | 137756 | 141827 | 588812 | 584663 | 0 | 5853 |
| mtChr1 | *PQ580749.1* | 95.478 | 3140 | 100 | 20 | 36086 | 39197 | 27988 | 31113 | 0 | 4974 |
| mtChr1 | *PQ580749.1* | 93.735 | 2889 | 119 | 21 | 146446 | 149284 | 282244 | 285120 | 0 | 4276 |
| mtChr1 | *PQ580749.1* | 93.109 | 2293 | 105 | 22 | 47868 | 50136 | 560530 | 558267 | 0 | 3310 |
| mtChr1 | *PQ580749.1* | 92.002 | 2213 | 110 | 25 | 154570 | 156728 | 375946 | 373747 | 0 | 3044 |
| mtChr1 | *PQ580749.1* | 93.753 | 1953 | 73 | 19 | 102434 | 104363 | 186924 | 184998 | 0 | 2885 |
| mtChr1 | *PQ580749.1* | 92.026 | 1994 | 113 | 18 | 94424 | 96395 | 110534 | 108565 | 0 | 2760 |
| mtChr1 | *PQ580749.1* | 96.283 | 1668 | 62 | 0 | 23324 | 24991 | 147726 | 146059 | 0 | 2737 |
| mtChr1 | *PQ580749.1* | 95.62 | 1598 | 29 | 14 | 75371 | 76935 | 633881 | 635470 | 0 | 2525 |
| mtChr1 | *PQ580749.1* | 91.204 | 1785 | 82 | 34 | 135223 | 136959 | 591771 | 590014 | 0 | 2357 |
| mtChr1 | *PQ580749.1* | 92.923 | 1625 | 71 | 15 | 32360 | 33942 | 182364 | 180742 | 0 | 2324 |
| mtChr1 | *PQ580749.1* | 97.55 | 1347 | 23 | 4 | 76928 | 78273 | 635594 | 636931 | 0 | 2296 |
| mtChr1 | *PQ580749.1* | 91.788 | 1644 | 81 | 25 | 120291 | 121902 | 437567 | 435946 | 0 | 2239 |
| mtChr1 | *PQ580749.1* | 90.133 | 1723 | 61 | 28 | 100658 | 102308 | 189588 | 187903 | 0 | 2139 |
| mtChr1 | *PQ580749.1* | 91.808 | 1538 | 117 | 6 | 114775 | 116307 | 696035 | 697568 | 0 | 2134 |
| mtChr1 | *PQ580749.1* | 96.659 | 1257 | 32 | 4 | 121900 | 123152 | 435865 | 434615 | 0 | 2080 |
| mtChr1 | *PQ580749.1* | 89.67 | 1607 | 77 | 28 | 123151 | 124693 | 434412 | 432831 | 0 | 1965 |
| mtChr1 | *PQ580749.1* | 95.447 | 1208 | 53 | 1 | 46509 | 47714 | 141700 | 140493 | 0 | 1925 |
| mtChr1 | *PQ580749.1* | 91.529 | 1393 | 64 | 20 | 111693 | 113073 | 24683 | 26033 | 0 | 1869 |
| mtChr1 | *PQ580749.1* | 82.98 | 2074 | 263 | 54 | 91166 | 93184 | 700659 | 698621 | 0 | 1792 |
| mtChr1 | *PQ580749.1* | 90.48 | 1292 | 73 | 20 | 14713 | 15958 | 419357 | 420644 | 0 | 1659 |
| mtChr1 | *PQ580749.1* | 91.97 | 1183 | 56 | 16 | 31137 | 32288 | 183531 | 182357 | 0 | 1622 |
| mtChr1 | *PQ580749.1* | 94.737 | 950 | 35 | 6 | 61703 | 62637 | 302120 | 303069 | 0 | 1463 |
| mtChr1 | *PQ580749.1* | 95.519 | 915 | 40 | 1 | 110632 | 111546 | 23214 | 24127 | 0 | 1461 |
| mtChr1 | *PQ580749.1* | 88.642 | 1259 | 65 | 30 | 68582 | 69782 | 575067 | 573829 | 0 | 1461 |
| mtChr1 | *PQ580749.1* | 88.935 | 1202 | 77 | 19 | 132866 | 134033 | 363189 | 364368 | 0 | 1432 |
| mtChr1 | *PQ580749.1* | 93.487 | 952 | 38 | 8 | 69820 | 70757 | 573631 | 572690 | 0 | 1393 |
| mtChr1 | *PQ580749.1* | 90.524 | 1087 | 48 | 22 | 119251 | 120293 | 438797 | 437722 | 0 | 1386 |
| mtChr1 | *PQ580749.1* | 95.412 | 850 | 39 | 0 | 131255 | 132104 | 13321 | 14170 | 0 | 1354 |
| mtChr1 | *PQ580749.1* | 93.483 | 890 | 43 | 6 | 124716 | 125592 | 432785 | 431898 | 0 | 1308 |
| mtChr1 | *PQ580749.1* | 93.016 | 902 | 45 | 6 | 81819 | 82708 | 217272 | 218167 | 0 | 1301 |
| mtChr1 | *PQ580749.1* | 95.426 | 809 | 33 | 4 | 116347 | 117151 | 68476 | 67668 | 0 | 1286 |
| mtChr1 | *PQ580749.1* | 93.56 | 823 | 27 | 9 | 17174 | 17981 | 508517 | 507706 | 0 | 1203 |
| mtChr1 | *PQ580749.1* | 92.462 | 849 | 38 | 14 | 41598 | 42422 | 289638 | 288792 | 0 | 1190 |
| mtChr1 | *PQ580749.1* | 93.799 | 758 | 47 | 0 | 10393 | 11150 | 67342 | 66585 | 0 | 1140 |
| mtChr1 | *PQ580749.1* | 95.056 | 708 | 33 | 1 | 40744 | 41449 | 290882 | 290175 | 0 | 1112 |
| mtChr1 | *PQ580749.1* | 91.278 | 814 | 64 | 5 | 113562 | 114371 | 127188 | 126378 | 0 | 1103 |
| mtChr1 | *PQ580749.1* | 94.412 | 680 | 29 | 5 | 18255 | 18925 | 709469 | 710148 | 0 | 1037 |
| mtChr1 | *PQ580749.1* | 94.412 | 680 | 29 | 5 | 105377 | 106047 | 709469 | 710148 | 0 | 1037 |
| mtChr1 | *PQ580749.1* | 88.738 | 808 | 49 | 20 | 27861 | 28629 | 138556 | 139360 | 0 | 950 |
| mtChr1 | *PQ580749.1* | 91.667 | 672 | 48 | 5 | 88145 | 88816 | 52964 | 53627 | 0 | 924 |
| mtChr1 | *PQ580749.1* | 92.787 | 610 | 41 | 2 | 84962 | 85568 | 711023 | 710414 | 0 | 880 |
| mtChr1 | *PQ580749.1* | 95.03 | 503 | 21 | 4 | 50946 | 51446 | 218731 | 218231 | 0 | 787 |
| mtChr1 | *PQ580749.1* | 90.878 | 592 | 36 | 6 | 134129 | 134702 | 594023 | 593432 | 0 | 778 |
| mtChr1 | *PQ580749.1* | 88.546 | 681 | 25 | 14 | 16482 | 17123 | 422214 | 422880 | 0 | 776 |
| mtChr1 | *PQ580749.1* | 92.115 | 520 | 23 | 9 | 15945 | 16458 | 421451 | 421958 | 0 | 717 |
| mtChr1 | *PQ580749.1* | 88.246 | 553 | 59 | 6 | 87140 | 87690 | 11813 | 12361 | 0 | 656 |
| mtChr1 | *PQ580749.1* | 89.041 | 511 | 35 | 9 | 134695 | 135196 | 592456 | 591958 | 1.53E-174 | 614 |
| mtChr1 | *PQ580749.1* | 81.903 | 641 | 38 | 25 | 62629 | 63218 | 303280 | 303893 | 3.50E-131 | 470 |
| mtChr1 | *OP588116.1* | 98.669 | 16223 | 136 | 23 | 67635 | 83821 | 194116 | 177938 | 0 | 28688 |
| mtChr1 | *OP588116.1* | 98.392 | 9204 | 57 | 10 | 35868 | 45021 | 123624 | 132786 | 0 | 16092 |
| mtChr1 | *OP588116.1* | 98.678 | 8549 | 78 | 9 | 88981 | 97528 | 22309 | 13795 | 0 | 15128 |
| mtChr1 | *OP588116.1* | 99.264 | 7334 | 45 | 6 | 45345 | 52673 | 132781 | 140110 | 0 | 13236 |
| mtChr1 | *OP588116.1* | 98.626 | 6771 | 43 | 13 | 100383 | 107131 | 66682 | 73424 | 0 | 11943 |
| mtChr1 | *OP588116.1* | 98.553 | 6429 | 62 | 12 | 109973 | 116389 | 112925 | 106516 | 0 | 11328 |
| mtChr1 | *OP588116.1* | 97.943 | 6562 | 71 | 17 | 145130 | 151657 | 84200 | 77669 | 0 | 11311 |
| mtChr1 | *OP588116.1* | 98.988 | 5928 | 45 | 3 | 22851 | 28777 | 168922 | 163009 | 0 | 10600 |
| mtChr1 | *OP588116.1* | 98.513 | 4573 | 47 | 8 | 55159 | 59717 | 7780 | 3215 | 0 | 8048 |
| mtChr1 | *OP588116.1* | 98.559 | 4510 | 42 | 9 | 29534 | 34028 | 116627 | 121128 | 0 | 7947 |
| mtChr1 | *OP588116.1* | 98.502 | 4472 | 46 | 9 | 118799 | 123252 | 150253 | 145785 | 0 | 7867 |
| mtChr1 | *OP588116.1* | 98.399 | 3497 | 28 | 6 | 140404 | 143884 | 89046 | 85562 | 0 | 6122 |
| mtChr1 | *OP588116.1* | 98.483 | 3295 | 36 | 4 | 13832 | 17121 | 157131 | 160416 | 0 | 5795 |
| mtChr1 | *OP588116.1* | 98.529 | 2923 | 40 | 2 | 97628 | 100550 | 194236 | 197155 | 0 | 5156 |
| mtChr1 | *OP588116.1* | 98.709 | 2711 | 24 | 5 | 134695 | 137402 | 94433 | 91731 | 0 | 4802 |
| mtChr1 | *OP588116.1* | 99.166 | 2519 | 18 | 1 | 137396 | 139911 | 91560 | 89042 | 0 | 4532 |
| mtChr1 | *OP588116.1* | 97.143 | 2695 | 41 | 4 | 151654 | 154318 | 35084 | 37772 | 0 | 4518 |
| mtChr1 | *OP588116.1* | 97.868 | 2580 | 26 | 10 | 129658 | 132208 | 104022 | 106601 | 0 | 4433 |
| mtChr1 | *OP588116.1* | 98.272 | 2488 | 30 | 3 | 84043 | 86518 | 177948 | 175462 | 0 | 4344 |
| mtChr1 | *OP588116.1* | 98.365 | 2447 | 19 | 1 | 154303 | 156728 | 49127 | 51573 | 0 | 4277 |
| mtChr1 | *OP588116.1* | 98.307 | 2303 | 30 | 4 | 5015 | 7314 | 58916 | 61212 | 0 | 4028 |
| mtChr1 | *OP588116.1* | 98.342 | 2231 | 29 | 3 | 123810 | 126037 | 145600 | 143375 | 0 | 3908 |
| mtChr1 | *OP588116.1* | 98.616 | 2167 | 29 | 1 | 11694 | 13859 | 154811 | 156977 | 0 | 3834 |
| mtChr1 | *OP588116.1* | 98.094 | 2204 | 18 | 5 | 61028 | 63213 | 2198 | 1 | 0 | 3816 |
| mtChr1 | *OP588116.1* | 97.054 | 2240 | 25 | 7 | 126347 | 128555 | 143357 | 141128 | 0 | 3733 |
| mtChr1 | *OP588116.1* | 98.777 | 1963 | 16 | 4 | 132742 | 134703 | 96593 | 94638 | 0 | 3485 |
| mtChr1 | *OP588116.1* | 98.763 | 1860 | 14 | 2 | 34018 | 35877 | 121398 | 123248 | 0 | 3299 |
| mtChr1 | *OP588116.1* | 98.701 | 1847 | 20 | 1 | 18163 | 20009 | 71582 | 73424 | 0 | 3275 |
| mtChr1 | *OP588116.1* | 99.262 | 1761 | 13 | 0 | 9940 | 11700 | 152750 | 154510 | 0 | 3181 |
| mtChr1 | *OP588116.1* | 98.357 | 1704 | 24 | 4 | 1960 | 3660 | 24530 | 26232 | 0 | 2988 |
| mtChr1 | *OP588116.1* | 98.043 | 1482 | 19 | 3 | 63214 | 64695 | 212159 | 210688 | 0 | 2567 |
| mtChr1 | *OP588116.1* | 97.49 | 1315 | 25 | 3 | 116290 | 117596 | 32436 | 33750 | 0 | 2239 |
| mtChr1 | *OP588116.1* | 99.097 | 1218 | 10 | 1 | 143874 | 145091 | 85399 | 84183 | 0 | 2187 |
| mtChr1 | *OP588116.1* | 96.836 | 1296 | 23 | 6 | 8664 | 9949 | 151303 | 152590 | 0 | 2150 |
| mtChr1 | *OP588116.1* | 99.149 | 1057 | 9 | 0 | 108157 | 109213 | 115847 | 114791 | 0 | 1903 |
| mtChr1 | *OP588116.1* | 99.149 | 1057 | 9 | 0 | 21035 | 22091 | 115847 | 114791 | 0 | 1903 |
| mtChr1 | *OP588116.1* | 99.149 | 1057 | 9 | 0 | 108157 | 109213 | 171844 | 170788 | 0 | 1903 |
| mtChr1 | *OP588116.1* | 99.149 | 1057 | 9 | 0 | 21035 | 22091 | 171844 | 170788 | 0 | 1903 |
| mtChr1 | *OP588116.1* | 98.401 | 1063 | 12 | 2 | 3959 | 5016 | 26854 | 27916 | 0 | 1864 |
| mtChr1 | *OP588116.1* | 98.218 | 1066 | 10 | 1 | 86649 | 87714 | 24546 | 23490 | 0 | 1855 |
| mtChr1 | *OP588116.1* | 97.848 | 1069 | 14 | 4 | 17112 | 18180 | 160657 | 161716 | 0 | 1838 |
| mtChr1 | *OP588116.1* | 99.567 | 923 | 3 | 1 | 88067 | 88989 | 23500 | 22579 | 0 | 1681 |
| mtChr1 | *OP588116.1* | 97.286 | 958 | 7 | 6 | 53856 | 54794 | 9431 | 8474 | 0 | 1607 |
| mtChr1 | *OP588116.1* | 98.649 | 888 | 7 | 1 | 60154 | 61036 | 3225 | 2338 | 0 | 1568 |
| mtChr1 | *OP588116.1* | 98.97 | 874 | 9 | 0 | 29534 | 30407 | 172624 | 173497 | 0 | 1565 |
| mtChr1 | *OP588116.1* | 97.816 | 824 | 4 | 3 | 1044 | 1857 | 52111 | 52930 | 0 | 1410 |
| mtChr1 | *OP588116.1* | 98.25 | 800 | 10 | 1 | 107114 | 107913 | 47243 | 46448 | 0 | 1397 |
| mtChr1 | *OP588116.1* | 98.25 | 800 | 10 | 1 | 19992 | 20791 | 47243 | 46448 | 0 | 1397 |
| mtChr1 | *OP588116.1* | 99.694 | 654 | 2 | 0 | 22851 | 23504 | 112925 | 112272 | 0 | 1197 |
| mtChr1 | *OP588116.1* | 99.694 | 654 | 2 | 0 | 109973 | 110626 | 168922 | 168269 | 0 | 1197 |
| mtChr1 | *OP588116.1* | 99.328 | 595 | 4 | 0 | 109296 | 109890 | 114351 | 113757 | 0 | 1077 |
| mtChr1 | *OP588116.1* | 99.328 | 595 | 4 | 0 | 22174 | 22768 | 114351 | 113757 | 0 | 1077 |
| mtChr1 | *OP588116.1* | 99.328 | 595 | 4 | 0 | 109296 | 109890 | 170348 | 169754 | 0 | 1077 |
| mtChr1 | *OP588116.1* | 99.328 | 595 | 4 | 0 | 22174 | 22768 | 170348 | 169754 | 0 | 1077 |
| mtChr1 | *OP588116.1* | 98.182 | 605 | 9 | 2 | 117830 | 118432 | 28534 | 29138 | 0 | 1055 |
| mtChr1 | *OP588116.1* | 97.575 | 536 | 5 | 1 | 28803 | 29338 | 116103 | 116630 | 0 | 911 |
| mtChr1 | *OP588116.1* | 97.575 | 536 | 5 | 1 | 28803 | 29338 | 172100 | 172627 | 0 | 911 |
| mtChr1 | *OP588116.1* | 97.02 | 537 | 9 | 3 | 1 | 530 | 51574 | 52110 | 0 | 896 |
| mtChr1 | *OP588116.1* | 71.959 | 888 | 203 | 36 | 153336 | 154197 | 62731 | 61864 | 3.88E-56 | 219 |
| mtChr1 | *NC_072122.1* | 96.833 | 4516 | 67 | 21 | 137384 | 141827 | 216347 | 220858 | 0 | 7478 |
| mtChr1 | *NC_072122.1* | 97.74 | 4204 | 46 | 9 | 45873 | 50050 | 192934 | 188754 | 0 | 7191 |
| mtChr1 | *NC_072122.1* | 97.055 | 4245 | 74 | 15 | 118881 | 123090 | 96501 | 92273 | 0 | 7099 |
| mtChr1 | *NC_072122.1* | 97.607 | 3761 | 35 | 13 | 35867 | 39572 | 123282 | 119522 | 0 | 6396 |
| mtChr1 | *NC_072122.1* | 97.875 | 3294 | 46 | 5 | 146405 | 149685 | 104203 | 100921 | 0 | 5674 |
| mtChr1 | *NC_072122.1* | 96.219 | 3253 | 102 | 10 | 113078 | 116319 | 51116 | 54358 | 0 | 5306 |
| mtChr1 | *NC_072122.1* | 97.16 | 3099 | 76 | 7 | 90744 | 93841 | 196064 | 199151 | 0 | 5225 |
| mtChr1 | *NC_072122.1* | 95.156 | 3303 | 59 | 21 | 41555 | 44762 | 172678 | 175974 | 0 | 5120 |
| mtChr1 | *NC_072122.1* | 98.003 | 2905 | 44 | 8 | 31137 | 34027 | 25371 | 28275 | 0 | 5031 |
| mtChr1 | *NC_072122.1* | 97.942 | 2770 | 31 | 5 | 68014 | 70757 | 133628 | 136397 | 0 | 4776 |
| mtChr1 | *NC_072122.1* | 96.381 | 2763 | 34 | 16 | 134695 | 137402 | 213441 | 216192 | 0 | 4488 |
| mtChr1 | *NC_072122.1* | 97.255 | 2295 | 56 | 6 | 94024 | 96314 | 153240 | 155531 | 0 | 3882 |
| mtChr1 | *NC_072122.1* | 97.664 | 2183 | 26 | 2 | 154567 | 156728 | 73128 | 75306 | 0 | 3725 |
| mtChr1 | *NC_072122.1* | 96.303 | 2245 | 45 | 9 | 50209 | 52420 | 35179 | 37418 | 0 | 3651 |
| mtChr1 | *NC_072122.1* | 96.234 | 2018 | 56 | 10 | 17107 | 19106 | 237708 | 239723 | 0 | 3288 |
| mtChr1 | *NC_072122.1* | 97.897 | 1807 | 28 | 4 | 132907 | 134703 | 211294 | 213100 | 0 | 3118 |
| mtChr1 | *NC_072122.1* | 97.48 | 1786 | 41 | 3 | 123789 | 125572 | 92278 | 90495 | 0 | 3046 |
| mtChr1 | *NC_072122.1* | 98.66 | 1717 | 16 | 3 | 102645 | 104360 | 243610 | 241900 | 0 | 3037 |
| mtChr1 | *NC_072122.1* | 97.52 | 1774 | 38 | 4 | 14713 | 16480 | 234888 | 236661 | 0 | 3027 |
| mtChr1 | *NC_072122.1* | 94.023 | 2041 | 49 | 9 | 39594 | 41561 | 170605 | 172645 | 0 | 3025 |
| mtChr1 | *NC_072122.1* | 97.725 | 1758 | 31 | 5 | 23236 | 24989 | 12568 | 14320 | 0 | 3016 |
| mtChr1 | *NC_072122.1* | 96.812 | 1725 | 24 | 8 | 100567 | 102261 | 245327 | 243604 | 0 | 2852 |
| mtChr1 | *NC_072122.1* | 98.414 | 1576 | 23 | 2 | 10075 | 11649 | 148323 | 149897 | 0 | 2771 |
| mtChr1 | *NC_072122.1* | 97.099 | 1620 | 18 | 8 | 75212 | 76803 | 224728 | 226346 | 0 | 2704 |
| mtChr1 | *NC_072122.1* | 97.08 | 1541 | 25 | 3 | 84160 | 85689 | 39216 | 40747 | 0 | 2579 |
| mtChr1 | *NC_072122.1* | 99.496 | 1388 | 7 | 0 | 76911 | 78298 | 226381 | 227768 | 0 | 2525 |
| mtChr1 | *NC_072122.1* | 96.062 | 1473 | 44 | 6 | 71149 | 72609 | 137664 | 139134 | 0 | 2386 |
| mtChr1 | *NC_072122.1* | 97.705 | 1351 | 18 | 7 | 111725 | 113073 | 49345 | 50684 | 0 | 2311 |
| mtChr1 | *NC_072122.1* | 95.291 | 1253 | 33 | 4 | 129658 | 130884 | 182334 | 181082 | 0 | 1964 |
| mtChr1 | *NC_072122.1* | 95.258 | 1202 | 24 | 10 | 80097 | 81274 | 4683 | 5875 | 0 | 1873 |
| mtChr1 | *NC_072122.1* | 98.448 | 1031 | 13 | 2 | 146405 | 147434 | 65171 | 64143 | 0 | 1812 |
| mtChr1 | *NC_072122.1* | 98.794 | 995 | 12 | 0 | 81259 | 82253 | 6424 | 7418 | 0 | 1772 |
| mtChr1 | *NC_072122.1* | 96.164 | 1095 | 18 | 11 | 110622 | 111702 | 47833 | 48917 | 0 | 1768 |
| mtChr1 | *NC_072122.1* | 96.359 | 1071 | 34 | 1 | 78303 | 79368 | 227946 | 229016 | 0 | 1757 |
| mtChr1 | *NC_072122.1* | 95.421 | 1070 | 31 | 4 | 1 | 1053 | 75307 | 76375 | 0 | 1688 |
| mtChr1 | *NC_072122.1* | 97.718 | 964 | 21 | 1 | 131206 | 132168 | 233011 | 233974 | 0 | 1657 |
| mtChr1 | *NC_072122.1* | 93.504 | 1093 | 44 | 2 | 44805 | 45875 | 194048 | 192961 | 0 | 1600 |
| mtChr1 | *NC_072122.1* | 97.439 | 937 | 17 | 4 | 105294 | 106228 | 238792 | 239723 | 0 | 1591 |
| mtChr1 | *NC_072122.1* | 98.372 | 860 | 10 | 1 | 61782 | 62637 | 201688 | 202547 | 0 | 1507 |
| mtChr1 | *NC_072122.1* | 96.095 | 922 | 24 | 3 | 89597 | 90507 | 78203 | 77283 | 0 | 1493 |
| mtChr1 | *NC_072122.1* | 97.917 | 864 | 14 | 4 | 27776 | 28636 | 147607 | 146745 | 0 | 1493 |
| mtChr1 | *NC_072122.1* | 97.235 | 868 | 19 | 4 | 25867 | 26731 | 86112 | 86977 | 0 | 1465 |
| mtChr1 | *NC_072122.1* | 98.464 | 781 | 12 | 0 | 116360 | 117140 | 58358 | 59138 | 0 | 1376 |
| mtChr1 | *NC_072122.1* | 92.292 | 999 | 32 | 14 | 62629 | 63584 | 202764 | 203760 | 0 | 1376 |
| mtChr1 | *NC_072122.1* | 96.305 | 812 | 15 | 5 | 82257 | 83054 | 37614 | 38424 | 0 | 1319 |
| mtChr1 | *NC_072122.1* | 95.981 | 821 | 15 | 2 | 73030 | 73837 | 140091 | 140906 | 0 | 1317 |
| mtChr1 | *NC_072122.1* | 95.968 | 744 | 29 | 1 | 97930 | 98672 | 254817 | 255560 | 0 | 1206 |
| mtChr1 | *NC_072122.1* | 91.514 | 872 | 36 | 7 | 8665 | 9506 | 79578 | 78715 | 0 | 1166 |
| mtChr1 | *NC_072122.1* | 98.171 | 656 | 12 | 0 | 88151 | 88806 | 89102 | 89757 | 0 | 1146 |
| mtChr1 | *NC_072122.1* | 94.631 | 745 | 10 | 2 | 150942 | 151657 | 100290 | 99547 | 0 | 1127 |
| mtChr1 | *NC_072122.1* | 96.45 | 676 | 19 | 4 | 83050 | 83720 | 38449 | 39124 | 0 | 1110 |
| mtChr1 | *NC_072122.1* | 95.809 | 692 | 20 | 3 | 142793 | 143475 | 204612 | 203921 | 0 | 1109 |
| mtChr1 | *NC_072122.1* | 97.504 | 641 | 16 | 0 | 86094 | 86734 | 143841 | 143201 | 0 | 1096 |
| mtChr1 | *NC_072122.1* | 97.072 | 649 | 18 | 1 | 16472 | 17119 | 236826 | 237474 | 0 | 1092 |
| mtChr1 | *NC_072122.1* | 94.752 | 705 | 13 | 6 | 56151 | 56845 | 152035 | 152725 | 0 | 1075 |
| mtChr1 | *NC_072122.1* | 97.208 | 573 | 11 | 2 | 4092 | 4659 | 88532 | 89104 | 0 | 965 |
| mtChr1 | *NC_072122.1* | 98.315 | 534 | 9 | 0 | 14186 | 14719 | 233972 | 234505 | 0 | 937 |
| mtChr1 | *NC_072122.1* | 97.74 | 531 | 12 | 0 | 87180 | 87710 | 230419 | 230949 | 0 | 915 |
| mtChr1 | *NC_072122.1* | 95.219 | 502 | 8 | 3 | 34579 | 35070 | 22946 | 22451 | 0 | 780 |
