# Supplementary material for: Characterization and evolutionary insights into complete mitochondrial genome of Sedum sarmentosum within the family Crassulaceae
Source: Front Plant Sci. 2026 Feb 6;17:1710625. doi: 10.3389/fpls.2026.1710625 (PMC12920544; doi:10.3389/fpls.2026.1710625)
Supplement: Supplementary file 10 [file Table10.docx]

**Table S10 | Changes in the hydrophobicity of amino acids induced by RNA-editing.**

| **Type** | **RNA-editing** | **Number** | **Percentage** |
| --- | --- | --- | --- |
| hydrophilic- hydrophilic | CAC(H) → TAC(Y) | 15 |  |
|  | CAT (H) → TAT (Y) | 19 |  |
|  | CGC (R) → TGC (C) | 11 |  |
|  | CGT (R) → TGT (C) | 28 |  |
|  | total | 73 | 14.72% |
| hydrophilic- hydrophobic | ACA (T) → ATA (I) | 4 |  |
|  | ACT (T) → ATT (I) | 3 |  |
|  | ACT (T) → Met (M) | 2 |  |
|  | CGG (R) → TGG (W) | 36 |  |
|  | TCA (S) → TTA (L) | 49 |  |
|  | TCG (S) → TTG (L) | 39 |  |
|  | TCC (S) → TTC (F) | 23 |  |
|  | TCT (S) → TTT (F) | 43 |  |
|  | total | 199 | 40.12% |
| hydrophobic- hydrophilic | CCA (P) → TCA (S) | 12 |  |
|  | CCC (P) → TCC (S) | 18 |  |
|  | CCG (P) → TCG (S) | 12 |  |
|  | CCT (P) → TCT (S) | 17 |  |
|  | total | 59 | 11.90% |
| hydrophobic- hydrophobic | CCA (P) → CTA (L) | 39 |  |
|  | CCC (P) → CTC (L) | 11 |  |
|  | CCG (P) → CTG (L) | 33 |  |
|  | CCT (P) → CTT (L) | 30 |  |
|  | CTC (L) → TTC (F) | 14 |  |
|  | CTT (L) → TTT (F) | 14 |  |
|  | GCA (A) → GTA (V) | 1 |  |
|  | GCC (A) → GTC (V) | 1 |  |
|  | GCG (A) → GTG (V) | 2 |  |
|  | GCU (A) → GTG (V) | 1 |  |
|  | total | 146 | 29.44% |
|  | CAA (Q) → TAA (*) | 13 |  |
|  | Arg (R) → TGA (*) | 6 |  |
|  | total | 19 | 3.83% |
|  | all | 496 | 100.00% |
