# Supplementary material for: Characterization and evolutionary insights into complete mitochondrial genome of Sedum sarmentosum within the family Crassulaceae
Source: Front Plant Sci. 2026 Feb 6;17:1710625. doi: 10.3389/fpls.2026.1710625 (PMC12920544; doi:10.3389/fpls.2026.1710625)
Supplement: Supplementary file 11 [file Table11.docx]

**Table S11 | Closely related species of *Sedum sarmentosum*.**

| **Order** | **Name** | **NCBI** |
| --- | --- | --- |
| Caryophyllales | *Beta macrocarpa* | NC_015994 |
|  | *Suaeda glauca* | NC_060419 |
|  | *Salicornia ramosissima* | PP974652 |
|  | *Mesembryanthemum crystallinum* | NC_086673 |
|  | *Tetragonia tetragonoides* | MW971440 |
|  | *Sesuvium portulacastrum* | MN683736 |
|  | *Tamarix austromongolica* | PQ165103 |
|  | *Myricaria laxiflora* | NC_086644 |
|  | *Fallopia aubertii* | MW664926 |
|  | *Rheum webbianum* | PQ469560 |
|  | *Rheum palmatum* | NC_082039 |
|  | *Nepenthes ventricosa x Nepenthes alata* | NC_039531 |
| Saxifragales | *Paeonia suffruticosa* | NC_084129 |
|  | *Paeonia lactiflora* | NC_070189 |
|  | *Myriophyllum ussuriense* | PQ580749 |
|  | *Sedum plumbizincicola* | NC_069572 |
|  | *Rhodiola tangutica* | NC_072122 |
|  | *Rhodiola rosea* | PP024540 |
|  | *Rhodiola juparensis* | NC_082108 |
|  | *Rhodiola crenulata* | NC_070303 |
| Santalales | *Malania oleifera* | NC_053625 |
|  | *Viscum album* | NC_029039 |
|  | *Tolypanthus maclurei* | NC_056836 |
|  | *Santalum album* | NC_081498 |
| Rosids | *Manihot esculenta* | NC_045136 |
|  |  |  |
